# Application of Genome Wide Association and Genomic Prediction for Improvement of Cacao Productivity and Resistance to Black and Frosty Pod Diseases

**DOI:** 10.3389/fpls.2017.01905

**Published:** 2017-11-14

**Authors:** J. Alberto Romero Navarro, Wilbert Phillips-Mora, Adriana Arciniegas-Leal, Allan Mata-Quirós, Niina Haiminen, Guiliana Mustiga, Donald Livingstone III, Harm van Bakel, David N. Kuhn, Laxmi Parida, Andrew Kasarskis, Juan C. Motamayor

**Affiliations:** ^1^Mars Incorporated, Miami, FL, United States; ^2^Tropical Agricultural Research and Higher Education Center, Turrialba, Costa Rica; ^3^IBM Thomas J. Watson Research Center, New York, NY, United States; ^4^Icahn School of Medicine at Mount Sinai, Icahn Institute of Genomics and Multiscale Biology, New York, NY, United States; ^5^Subtropical Horticulture Research Station, United States Department of Agriculture-Agricultural Research Service, Miami, FL, United States

**Keywords:** *Theobroma cacao*, tree crops, breeding, disease resistance, genomic selection, linkage disequilibrium, differential expression

## Abstract

Chocolate is a highly valued and palatable confectionery product. Chocolate is primarily made from the processed seeds of the tree species *Theobroma cacao*. Cacao cultivation is highly relevant for small-holder farmers throughout the tropics, yet its productivity remains limited by low yields and widespread pathogens. A panel of 148 improved cacao clones was assembled based on productivity and disease resistance, and phenotypic single-tree replicated clonal evaluation was performed for 8 years. Using high-density markers, the diversity of clones was expressed relative to 10 known ancestral cacao populations, and significant effects of ancestry were observed in productivity and disease resistance. Genome-wide association (GWA) was performed, and six markers were significantly associated with frosty pod disease resistance. In addition, genomic selection was performed, and consistent with the observed extensive linkage disequilibrium, high predictive ability was observed at low marker densities for all traits. Finally, quantitative trait locus mapping and differential expression analysis of two cultivars with contrasting disease phenotypes were performed to identify genes underlying frosty pod disease resistance, identifying a significant quantitative trait locus and 35 differentially expressed genes using two independent differential expression analyses. These results indicate that in breeding populations of heterozygous and recently admixed individuals, mapping approaches can be used for low complexity traits like pod color cacao, or in other species single gene disease resistance, however genomic selection for quantitative traits remains highly effective relative to mapping. Our results can help guide the breeding process for sustainable improved cacao productivity.

## Introduction

The perennial tree *Theobroma cacao* is an important crop for tropical small-holder farmers. Cacao's fermented and dried seeds are used as raw material for a variety of products, particularly chocolate. Worldwide, low yields and high disease pressure are two major challenges that limit cacao production, resulting in increased deforestation and increased use of chemical pesticides, which has raised concerns regarding practices required for sustainable crop production (Clough et al., 2009, 2011). Although genetic markers have been used in many crops as tools to successfully increase the efficiency of breeding efforts, guide the use of germplasm resources (Tanksley and McCouch, 1997), and enhance traits such as disease resistance (St Clair, 2010), such efforts remain limited in cacao and other tropical perennial species.

Among cacao diseases, two that require careful consideration due to their prevalence and severity are frosty pod and black pod. Frosty pod is caused by the clonal pathogen *Moniliophthora roreri* (Díaz-Valderrama and Aime, 2016); this parasitic fungus is a sister species of *Moniliophthora perniciosa*, the fungus responsible for another devastating disease called witches' broom (Aime and Phillips-Mora, 2005). Under the right environmental conditions, frosty pod can destroy entire cacao plantations, and in addition to infecting cacao, frosty pod also affects all species of the closely related *Herrania* and *Theobroma* genera. Currently, frosty pod remains limited to areas in Central and South America, as well as some Caribbean islands, such as Jamaica; however, the range of reported cases has been expanding in recent decades, and its high virulence makes it a potential threat to cocoa production (Phillips-Mora and Wilkinson, 2007). Black pod in cacao can be caused by several species of *Phytophthora*, and losses due to black pod have been estimated to account for up to 25% of the expected global crop (Evans, 2007). In contrast to the geographically restricted range of frosty pod, black pod disease is ubiquitous (Despréaux, 2004), with *Phytophthora palmivora* being present in all cacao-growing regions (Brasier and Griffin, 1979). Unlike *M. roreri*, the presence of sexual structures (Vujičić, 1971) have shown that *P. palmivora* can undergo sexual reproduction, however similar to most *Phytophthora* species the majority of spores produced by *P. palmivora* are asexual (Judelson and Blanco, 2005). Although both diseases can be controlled to a certain extent through labor-intensive sanitation combined with optimal crop management practices(Evans, 2007), the use of clonal cultivars with genetic disease resistance can provide a cost- and labor-efficient approach for farmers to manage both diseases, and multiple QTL have been mapped for disease resistance in cacao (Brown et al., 2007; Lanaud et al., 2009).

Two complementary methods for the use of high-density genetic markers in breeding programs are genome-wide association (GWA) and genomic prediction. GWA allows for the identification of markers in linkage disequilibrium (LD) with polymorphisms that cause phenotypic changes and can be used for quantitative trait dissection in diverse populations (Hirschhorn and Daly, 2005). Significant markers can then be used in marker-assisted selection programs to more efficiently integrate favorable alleles into a germplasm pool, and GWA has yielded significant markers associated with multiple agronomic and morphological traits in cacao (Marcano et al., 2007, 2009; Motamayor et al., 2013; da Silva et al., 2016). Generally, GWA requires large number of individuals to tap into ancestral recombination for identifying variants associated with the phenotypes of interest, and although GWA is highly effective with low-complexity phenotypes, traits with many underlying genetic causal polymorphisms can be increasingly harder to identify, and complications such as false positives limit the application of this method. Unlike GWA, genomic prediction comprises a family of methods that rely on the association between phenotypes and genotypes on a training population to predict the trait value on a related set of individuals based only on genetic markers (Meuwissen et al., 2001). Genomic prediction does not rely on the direct identification of causal polymorphisms and can be highly effective for performing selection on polygenic traits or traits with significant genotype-by-environment interaction. Although both GWA and genomic prediction are widely used in plant and animal breeding programs, their application in cacao remains limited.

Here, we compared GWA and genomic prediction to identify associated markers and develop predictive models for frosty and black pod diseases, as well as yield traits on a population of 148 improved cacao clones. We collected phenotypic data from a multi-year evaluation trial with multiple individuals per clone and with data collection on a single-tree basis. We corroborated the pedigree relationships using markers and tested the effect of the rootstock on the yield. For GWA, we used pod color, a monogenic trait with a known candidate gene, as the positive control and compared the association results with the quantitative traits. The significant association with pod color shows the potential to identify the genetic basis of low-complexity traits on the studied cacao population. For frosty pod disease, significant regions were identified. Using genomic prediction, we estimated cross-validated prediction accuracies and evaluated the effect of marker density, observing good and stable prediction accuracies at low marker densities. We also performed differential expression analysis to narrow down genes with potential differences in response to frosty pod inoculation using pods from two parents with contrasting disease phenotypes from the clonal population. Differential expression was associated with cultivar rather than inoculation; however, some differentially expressed genes were identified near SNPs that were significantly associated with disease resistance, including a homolog of a putative disease resistance gene. Finally, we compared the association results with the quantitative trait loci mapping results from a family comprising parents from the clonal population evaluated at a different geographic location and observed a single significant quantitative trait locus.

## Results

### Diversity and structure

The major goals of this study were (1) to select individuals with improved productivity and resistance to frosty and black pod diseases and (2) to compare the performance of GWA and genomic prediction to characterize the genetic basis and to develop predictive models for those same phenotypes among high-yield cacao selections. A sample of 148 clones was assembled and evaluated in a multi-year trial with 25 plants per clone (Methods). The sample contained 35 representative individuals from the germplasm collection and 121 superior clones, representing crosses between 29 diverse parents (Methods, Supplementary Tables 1, 2). The 121 superior clones were chosen among closely related selections in order to maximize allele replication, which in turn placed most segregating alleles in high minor allele frequency (MAF) (Supplementary Figure 1), where there is the highest statistical power for association (Korte and Farlow, 2013). In total, the 121 improved clones contained 19 full-sib families, and the parents UF 273 and UF 712 were the most frequent, with eight and six families, respectively. At the time of the crosses, UF 273 was assumed to be single clone, however later analyses revealed that two very closely related genotypes were labeled under a single clone name. In addition to full sibs, significant relatedness was present in the form of half-sib relationships, with POUND 7, CATIE 1000, CC 137, TREE 81 and PA 169 being common to at least two families each.

In cacao, the presence of a complex genetic cross-incompatibility system, the difficulty of making crosses, and downstream human error have led to inconsistencies between the alleged parent-offspring relationships in breeding programs. Therefore, we first assessed the distribution of genetic diversity across the 148 clones and compared the pedigree of clones with their clustering based on markers. In general, good agreement was observed between the pedigrees and clustering of clones (Supplementary Figure 2), as only eight clones had potential parentage errors with a correct maternal parent but a potential error in the identity of the pollen donor.

**Figure 1:**
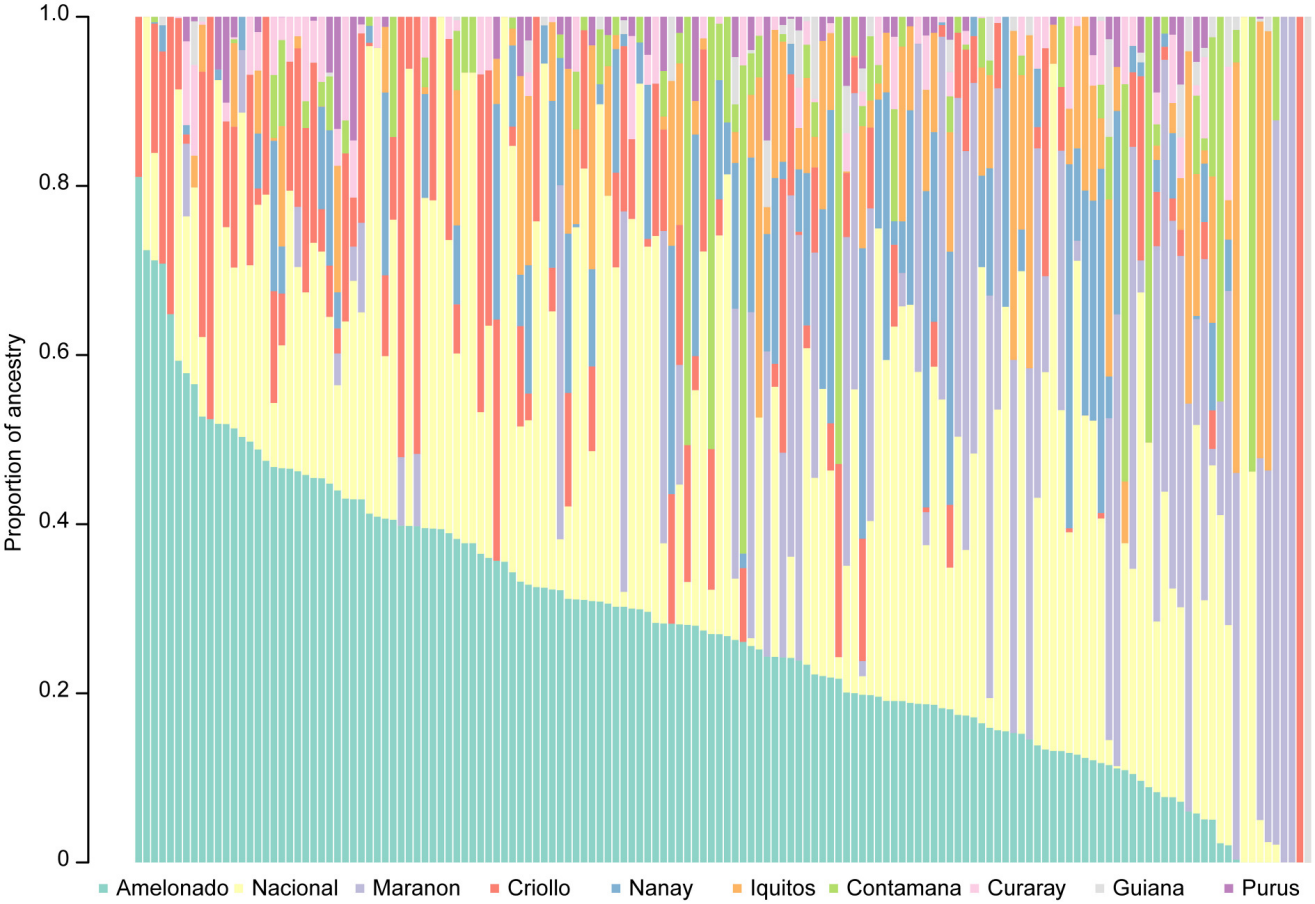
Proportion ancestry relative to the 10 genetic groups described by Motamayor et al. (2008) for all clones included in the trial.

Understanding the representation of diverse ancestral groups within the breeding germplasm is useful for breeding efforts. The selected clones were therefore characterized relative to cacao ancestral populations (Methods). The most represented genetic groups in the trial were Amelonado and Nacional, with an average proportion ancestry across clones of 0.27 (Figure 1, Supplementary Table 2). In addition to being highly represented among the selections, Nacional ancestry was significantly associated with the proportion of pods displaying symptoms for both frosty and black pod diseases, with resistance to the former and susceptibility to the latter (Supplementary Figure 3). Meanwhile, Marañon ancestry showed a significant negative correlation with the proportion of black and frosty pod infections (Supplementary Figure 4). The proportion of Nanay ancestry was positively correlated with yield (Supplementary Figure 5) whereas the proportion of Contamana was negatively correlated with yield (Supplementary Figure 6). The proportion of Criollo ancestry displayed a significant negative correlation with yield and healthy pod count (Supplementary Figure 7). No significant correlation was observed between the pod index and any ancestral population (adjusted *p*-value > 0.05).

**Figure 2:**
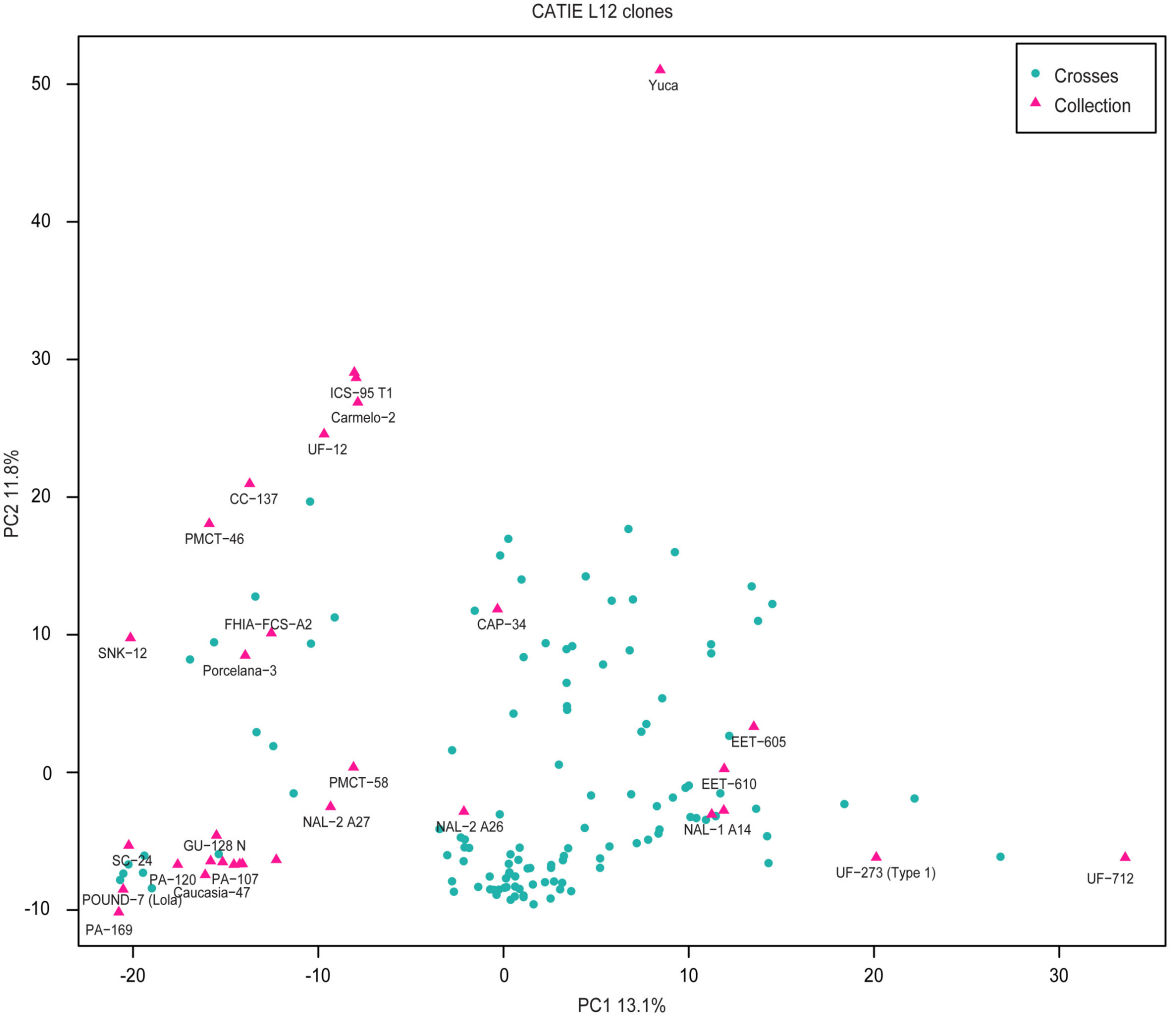
Principal component analysis of the genetic markers for all clones. Pink triangles represent clones from the germplasm collection. Blue dots correspond to the crosses.

Principal Component Analysis (Methods, Figure 2) was used to further characterize population structure. The first principal component, explaining 13.1% of the variance, separates the UF clones, which have a significant proportion of Nacional ancestry, from PA 169 and POUND 7, which represent Upper Amazon collections. The second component, explaining 11.8% of the variance, discriminates between the YUCA clone, a representative of the domesticated Criollo population, and clones of Amazonian origin. Most clones are positioned intermediately, consistent with their pedigree.

**Figure 3:**
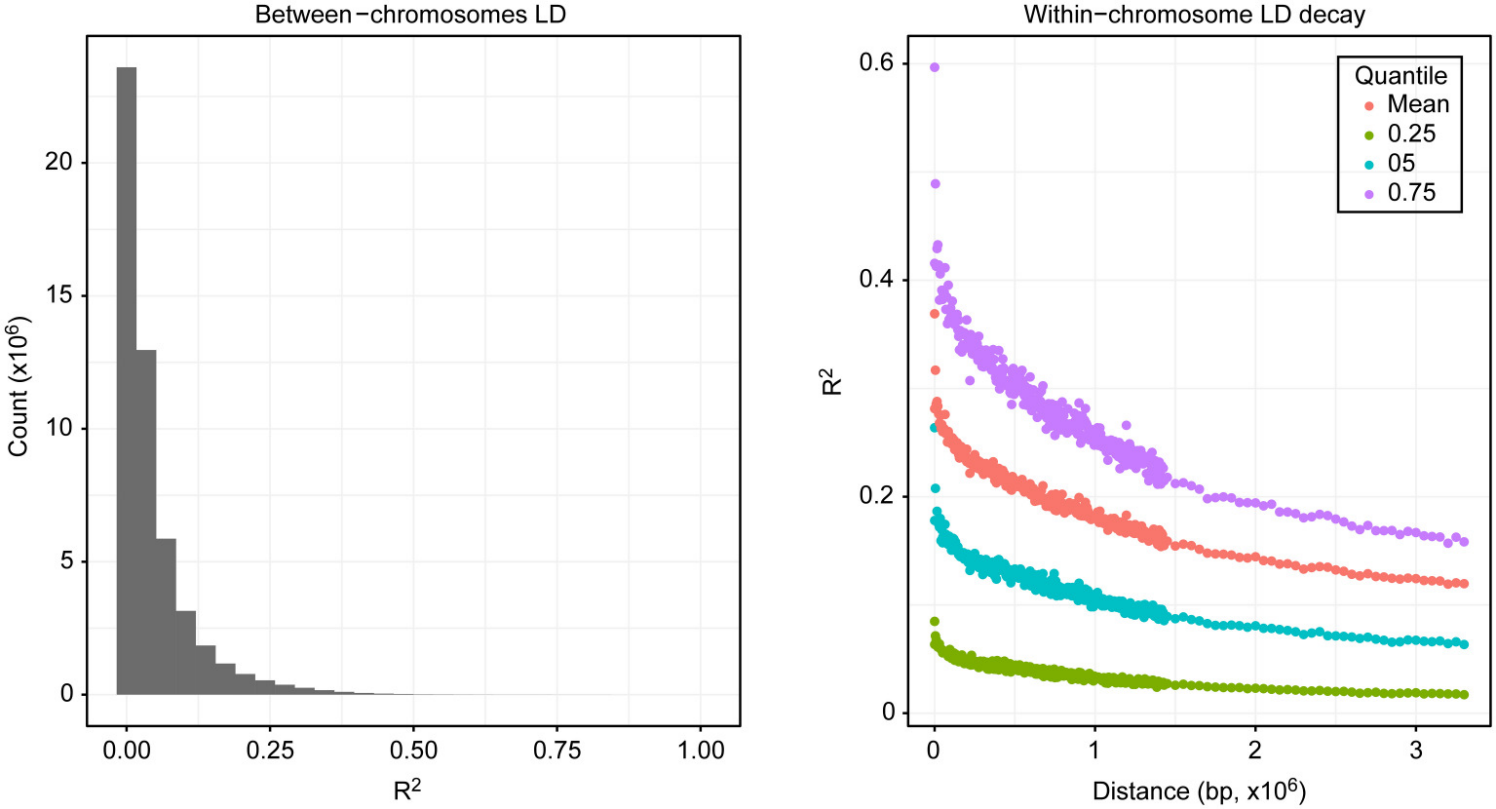
Linkage disequilibrium. The left side shows the distribution of the counts of correlation values for markers at different chromosomes, and the right side shows the correlation of markers within chromosomes relative to their distance.

In addition to the large-scale distribution of diversity, the correlation between markers (*R*^2^) was estimated both between and within chromosomes. Genetic correlations can exist between markers in different chromosomes, especially after recent admixture. In our population, most of the markers across chromosomes segregated independently; however, 0.13 of all pairwise comparisons between markers across chromosomes displayed *R*^2^ values > 0.1 (Figure 3). Extensive LD of markers within chromosomes was also observed, with average *R*^2^ values remaining >0.1 at a distance of 3.3 Mb (Figure 3).

### Phenotypic selection

A major objective of this work was to identify clones that combine high productivity with low incidence of black and frosty pod disease. The back-transformed adjusted clone means were estimated for all traits using mixed linear models (Methods, Supplementary Table 3). For yield, the heritability was 0.55, and the average yield per clone was 0.43 tons^*^ha^−1^yr^−1^. The top 10% of clones had yields >0.746 tons^*^ha^−1^yr^−1^, and the highest-yielding clone, CATIE R92, had an estimated yield of 1.39 tons^*^ha^−1^yr^−1^, 3.3 times the average. Sixty percent of the top-yielding clones had UF 273 as one of their parents. In addition to yield *per se*, pod index (number of pods needed to produce a kilogram of dried beans) is an important production trait, and heritability was 0.44 for the pod index, with a mean of 29 pods per kilogram of fermented dry beans. The top clones based on yield had good pod index values, with a mean of 22, a minimum of 15 and a maximum of 31 pods per kilogram of fermented dry beans.

To understand the potential of selection for clones with genetic resistance to black and frosty pod diseases, it was important to establish the heritability of all traits (Table 1). For frosty pod, we observed high disease pressure and high heritability for the transformed and back-transformed proportion of pods with disease symptoms (0.79 and 0.73, respectively). In contrast, black pod disease pressure was lower and the transformed count and proportion of pods with disease had higher heritability (0.55 and 0.52, respectively) than the non-transformed values (Supplementary Figure 8). Of the top 10% of clones with the lowest frosty pod disease proportion, only CATIE R5, CATIE R6, and CATIE R58 also ranked among the highest yielding clones. For black pod, the equivalent set contained CATIE R6 and CATIE R73. CATIE R5, and CATIE R6 are full-sibs, products of the cross between UF 273 and PA 169, while CATIE R58 is the product of CC 137 with UF 273; CATIE R73 is a highly black pod-resistant clone derived from crossing PA 169 and ARF 22, in turn a selection from the cross UF 613 and POUND 7.

**Table 1:** Heritability estimates and corresponding genomic selection prediction accuracies using all markers.

**Trait**	**Statistic**	**Transformation**	**Heritability (A)**	**Prediction accuracy**
FrostyPod	Proportion	Square root	0.73	0.67
FrostyPod	Proportion	Back-transformed	0.79	0.61
FrostyPod	Count	Square root	0.72	0.54
BlackPod	Proportion	Square root	0.52	0.51
BlackPod	Count	Square root	0.55	0.50
Yield	(HealthyPods / PodIndex)^*^1111	No transformation	0.55	0.48
HealthyPods	Count	Back-transformed	0.60	0.48
BlackPod	Proportion	Back-transformed	0.36	0.46
FrostyPod	Count	Back-transformed	0.67	0.46
HealthyPods	Count	Square root	0.62	0.44
BlackPod	Count	Back-transformed	0.35	0.43
PodIndex	Fruits per kg of dry beans	No transformation	0.44	0.37

In addition to productivity and disease resistance, it was of interest characterizing the effect of diversity within rootstocks on the productivity of the scion. Significant rootstock effects have been documented in many other tree crops (Warschefsky et al., 2016); however, the effect of rootstock choice in cacao remains largely uncharacterized. To generate rootstock seedlings in our trial, we obtained open-pollinated pods from SPA 9, IMC 67, EET 400, UF 613, and PA 121. The final rootstock/scion combination in the field is determined by the seedlings available during the collection of grafting material and afterwards, once grafting is performed, by survival. We observed a significant effect of rootstock on scion yield (*p*-value < 2e−16, Supplementary Table 4); relative to SPA 9, PA 121, and IMC 67 had a negative effect on the number of healthy pods, while EET 400 and UF 613 had a small positive effect on the total pod count.

### Genome-wide association and genomic prediction

**Figure 4:**
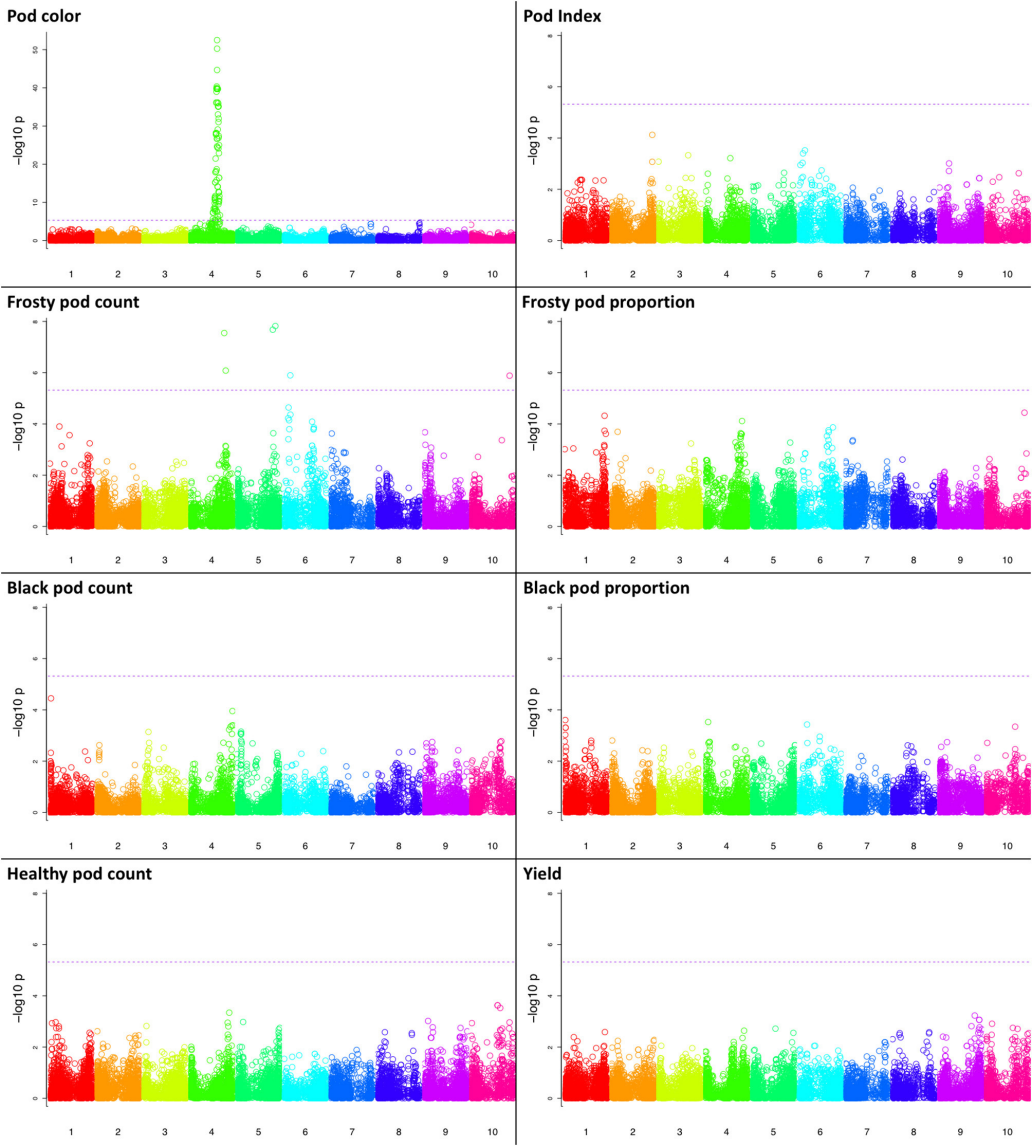
Manhattan plots of the genome wide association results for all traits. Along the x-axis are the genome positions (using the Matina genome assembly as reference) for the cacao chromosomes, with the chromosomes being color-coded. The y-axis corresponds to the *p*-value from the association model. The horizontal dotted line corresponds to the Bonferroni threshold for statistical significance.

GWA was performed for all traits (Methods), and we found a significant association of markers with two traits, pod color and frosty pod count, at the 0.05 significance threshold (Figure 4). For pod color, the most significant hit (*p*-value = 1e−52) was located adjacent to the *TcMYB113* gene on chromosome 4 at position 20,879,148, (coordinates relative to the Matina 1.6 assembly (Motamayor et al., 2013), which was shown to be significantly associated with pod color in an independent cacao population (Motamayor et al., 2013). In addition to pod color, six sites displayed significant associations with frosty pod at a 0.05 Bonferroni threshold; the positions, *p*-values, and annotations of the nearest genes are presented in Table 2.

**Table 2:** Top markers significantly associated with Frosty Pod disease, with nearest gene and corresponding annotation.

**Gene id**	**Chromosome**	**Start**	**End**	**Description**
TCM_020057	4	26415167	26437666	Trithorax-like protein 2 isoform 2
TCM_020262	4	27544908	27549168	Phosphate transporter 1,5
TCM_025566	5	33500077	33504228	Gamma-tocopherol methyltransferase
TCM_025986	5	35879522	35883596	Uncharacterized protein
TCM_027463	6	4238338	4241401	Kinase superfamily protein
TCM_045155	10	22681774	22690630	Uncharacterized protein

**Figure 5:**
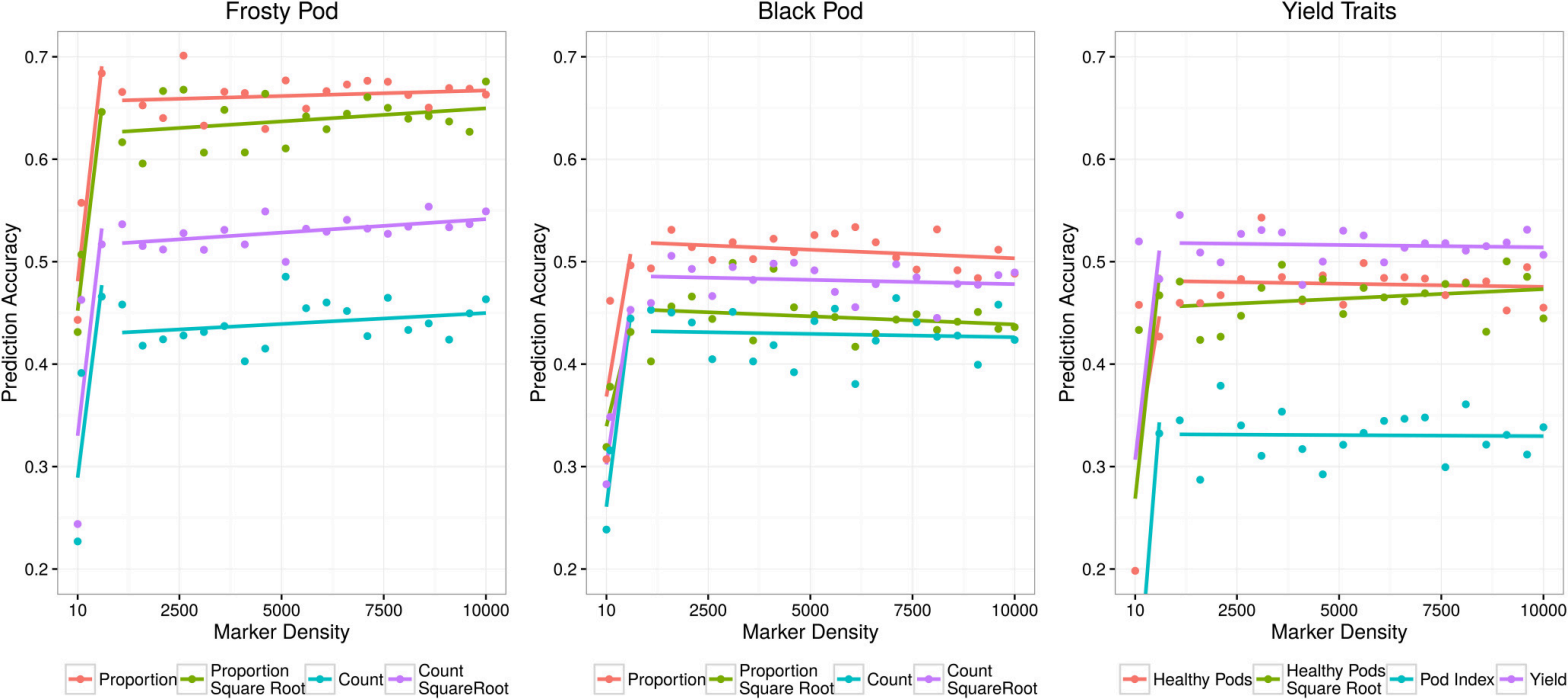
Genomic selection predictive abilities relative to the number of markers used to estimate each additive relationship matrix.

In genomic selection, breeding values can be predicted using markers due to their LD with causative polymorphisms. Given the high LD observed in the study population, we were interested in establishing the effect of marker density on predictive ability. We observed consistent predictive abilities within traits across marker densities, with modest increases relative to increased numbers of SNPs after 100 markers (Figure 5). The trait with the highest observed predictive ability was the proportion of frosty pod infection, with accuracies of 0.60 with 90 markers and 0.67 using all markers. As expected, most traits displayed predictive abilities slightly lower than the heritability estimates except for black pod; this effect is probably a result of low phenotypic variance due to low natural disease pressure in our experiment. Yield, a highly polygenic trait, displayed good predictive ability with all markers (0.48), while pod index displayed the lowest accuracy (0.3) at full marker density.

### Differential expression during early frosty pod infection

**Figure 6:**
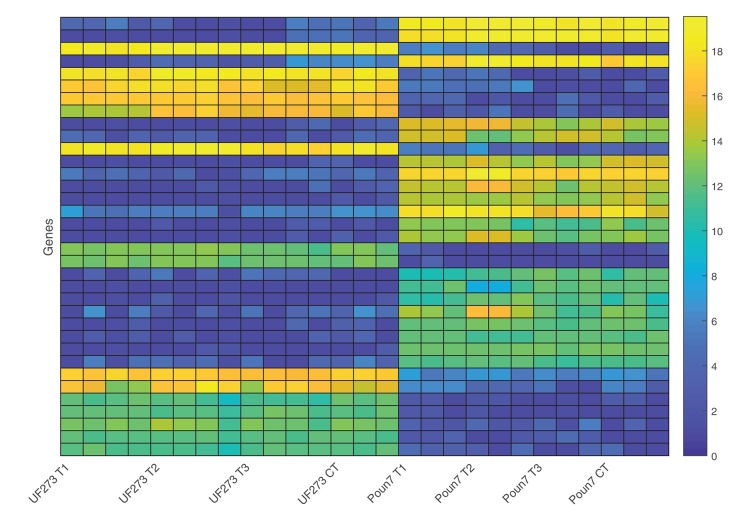
Visualization of expression magnitude using RoDEO for the 35 gene models with significant differential expression (RoDEO DE score > 10) among cultivars. Blue corresponds to low read counts, and yellow corresponds to high read counts for the genes, shown in order of decreasing DE. The UF 273 Type 1 and POUND 7 expression for these genes is clearly and consistently different. UF 273 Type 1: 4 frosty pod samples per time-point and three controls (one per timepoint). POUND 7: 3 frosty pod samples per time-point and three controls (one per time-point).

Differential gene expression can be used to detect genes with divergent expression relative to specific conditions, and several methods have been developed to statistically model and detect the genes with significant differences (Rapaport et al., 2013). A case-controlled study was conducted to investigate host-pathogen interaction through differential gene expression during the early stage of frosty pod infection. Clones UF273 Type 1 and POUND 7 were selected due to their divergent phenotypes for frosty pod; UF 273 Type 1 is resistant, and POUND 7 is highly susceptible. In order to detect differences in expression relative to the clone and inoculation treatment, two independent statistical approaches were performed, the first using RoDEO (Haiminen et al., 2014) and the second using voom/limma (Law et al., 2014; Ritchie et al., 2015) (Methods). Using both approaches, the only statistically significant expression differences detected were relative to clone, and no significant differences were detected relative to the inoculation treatment in either clone. The more conservative RoDEO analysis identified in the contrast between UF273 Type 1 and POUND 7 a total of 35 genes with differential expression scores greater than the highest observed score between biological replicates (Figure 6, Table 3); four genes were annotated as potentially involved in disease resistance. Using voom/limma, a total of 1,166 genes displayed significant differential expression between clones, with 566 and 600 genes up and down regulated in POUND 7 compared to UF273 Type 1, respectively (Supplementary Table 2). There were a total of 26 genes annotated as putative disease resistance loci in the voom/limma analysis, with 12 showing more than 20-fold differences in expression between UF and POUND 7. Overall, eight are upregulated and 18 are down-regulated in POUND 7 compared to UF. Good agreement was observed between the two approaches, with all of the 35 genes displaying significant differences using RoDEO also being significantly differentially expressed using voom/limma. When compared with the genome wide association results, 5 out of the 1,166 significantly differentially expressed genes are within 100 kilobases of significant genome wide significant association markers. The genes correspond to three uncharacterized proteins, as well as two proteins annotated respectively as a GroES-like zinc-binding dehydrogenase family protein and a Zinc-binding alcohol dehydrogenase family protein.

**Table 3:** Genes with significant cultivar-specific differential expression and corresponding annotations from the best alignments with other organisms.

**Gene name**	**Chr**	**Location**	**DE score**	**Match**	**Description**	**Organism**
Thecc1EG019813	4	25120947–25129801	18	HCBT2_DIACA	Anthranilate N-benzoyltransferase protein 2 OS = *Dianthus caryophyllus* GN = HCBT2 PE = 1 SV = 1	*Dianthus caryophyllus*
Thecc1EG020723	4	29906032–29907375	18	SOT17_ARATH	Sulfotransferase 17 OS = *Arabidopsis thaliana* GN = SOT17 PE = 1 SV = 1	*Arabidopsis thaliana*
Thecc1EG002057	1	11328347–11331355	−18	UBQ10_ARATH	Polyubiquitin 10 OS = *Arabidopsis thaliana* GN = UBQ10 PE = 1 SV = 2	*Arabidopsis thaliana*
Thecc1EG020810	4	30345815–30350891	17	CSLG2_ARATH	Cellulose synthase-like protein G2 OS = *Arabidopsis thaliana* GN = CSLG2 PE = 2 SV = 1	*Arabidopsis thaliana*
Thecc1EG026478	5	38501208–38513961	−17	DRL28_ARATH	Probable disease resistance protein At4g27220 OS = *Arabidopsis thaliana* GN = At4g27220 PE = 2 SV = 1	*Arabidopsis thaliana*
Thecc1EG029151	6	20964525–20965258	−17			
Thecc1EG006050	2	488650–492388	−16	Y3720_ARATH	UPF0481 protein At3g47200 OS = *Arabidopsis thaliana* GN = At3g47200 PE = 1 SV = 1	*Arabidopsis thaliana*
Thecc1EG018892	4	18789665–18793793	−15			
Thecc1EG024669	5	27713404–27714408	14			
Thecc1EG027121	6	1572161–1587220	14	TMVRN_NICGU	TMV resistance protein N OS = *Nicotiana glutinosa* GN = N PE = 1 SV = 1	*Nicotiana glutinosa*
Thecc1EG032160	7	9235234-9238567	−14	SOT16_ARATH	Sulfotransferase 16 OS = Arabidopsis thaliana GN = SOT16 PE = 1 SV = 1	*Arabidopsis thaliana*
Thecc1EG025134	5	30863215–30867012	13	AB31G_ARATH	ABC transporter G family member 31 OS = *Arabidopsis thaliana* GN = ABCG31 PE = 2 SV = 1	*Arabidopsis thaliana*
Thecc1EG029278	6	21682167–21684147	13			
Thecc1EG041240	9	36379797–36380643	13			
Thecc1EG045413	10r	24362994–24365283	13	R13L1_ARATH	Putative disease resistance RPP13-like protein 1 OS = *Arabidopsis thaliana* GN = RPPL1 PE = 2 SV = 1	*Arabidopsis thaliana*
Thecc1EG005676	1	37755066–37774390	12	Y3720_ARATH	UPF0481 protein At3g47200 OS = *Arabidopsis thaliana* GN = At3g47200 PE = 1 SV = 1	*Arabidopsis thaliana*
Thecc1EG005767	1	38160911–38163790	12			
Thecc1EG022565	5	5759068-5763625	12			
Thecc1EG001776	1	9401811–9404427	−12	C3H17_ORYSJ	Zinc finger CCCH domain-containing protein 17 OS = *Oryza sativa* subsp. *japonica* GN = Os02g0677700 PE = 2 SV = 2	*Oryza sativa*
Thecc1EG004338	1	31045794–31060808	−12			
Thecc1EG002064	1	11370876–11374204	11	PP402_ARATH	Putative pentatricopeptide repeat-containing protein At5g36300 OS = *Arabidopsis thaliana* GN = At5g36300 PE = 3 SV = 3	*Arabidopsis thaliana*
Thecc1EG009468	2	24224546–24226782	11	IAA32_ARATH	Auxin-responsive protein IAA32 OS = *Arabidopsis thaliana* GN = IAA32 PE = 2 SV = 2	*Arabidopsis thaliana*
Thecc1EG022647	5	6242029–6245268	11	INV1_DAUCA	Beta-fructofuranosidase, insoluble isoenzyme 1 OS = Daucus carota GN = INV1 PE = 1 SV = 1	*Daucus carota*
Thecc1EG031336	7	4278700–4286681	11			
Thecc1EG032589	7	12424993–12536706	11	Y3720_ARATH	UPF0481 protein At3g47200 OS = *Arabidopsis thaliana* GN = At3g47200 PE = 1 SV = 1	*Arabidopsis thaliana*
Thecc1EG038207	9	7718784–7720274	11			
Thecc1EG041903	9	40038459–40042732	11			
Thecc1EG042842	10r	2542591–2545439	11	FB129_ARATH	F-box protein At2g39490 OS = *Arabidopsis thaliana* GN = At2g39490 PE = 2 SV = 1	*Arabidopsis thaliana*
Thecc1EG010342	2	32772646–32795239	−11			
Thecc1EG017618	4	5375443–5379161	−11	GSTF7_ARATH	Glutathione S-transferase OS = *Arabidopsis thaliana* GN = At3g03190 PE = 2 SV = 1	*Arabidopsis thaliana*
Thecc1EG018865	4	18582593–18583434	−11			
Thecc1EG026484	5	38556578–38577058	−11	DRL27_ARATH	Disease-resistance protein At4g27190 OS = *Arabidopsis thaliana* GN = At4g27190 PE = 2 SV = 1	*Arabidopsis thaliana*
Thecc1EG027619	6	5484357–5507748	−11			
Thecc1EG029210	6	21336072–21338716	−11			
Thecc1EG040374	9	29618688–29638471	−11	PUB11_ARATH	U-box domain-containing protein 11 OS = *Arabidopsis thaliana* GN = PUB11 PE = 2 SV = 2	*Arabidopsis thaliana*

*The DE score represents the change in direction from UF 273 Type 1 to POUND 7*.

### Quantitative trait locus mapping

**Figure 7:**
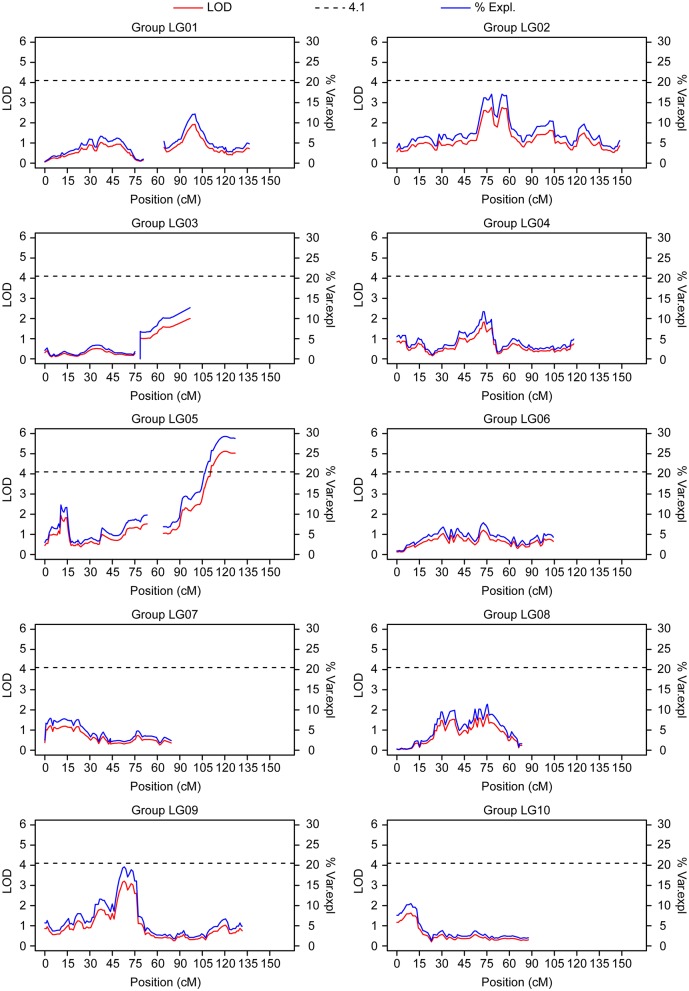
QTL mapping results from the population in La Montaña (POUND 7 × UF 273 Type 2) for frosty pod. Linkage groups correspond directly to chromosome number. A significant QTL is present on chromosome 5.

Perennial plants offer a good experimental setup for collecting multi-year information on the same individuals and independently analyzing the resulting phenotypic values. Here, we re-evaluated a previously analyzed population that was QTL mapped using frosty pod inoculation data (Brown et al., 2007) under natural field disease incidence. The population corresponded to the cross between the POUND 7 cultivar and two closely related clones, UF273 Type 1 and UF273 Type 2. Although UF273 was previously thought to be a single clone, individuals identified as UF273 correspond to two closely related clones, which we denote as UF273 Type 1 and UF273 Type 2. Both clones display a slight difference in their resistance to frosty pod disease, with UF273 Type 1 being more resistant. The mapping populations were analyzed independently (Methods) for the percentage of pods with frosty pod disease symptoms. For the POUND 7 × UF273 Type 2 population, the average percentage of frosty pod infection was only 0.56%; however, a significant association was observed at a single locus on chromosome 5, explaining 28.8% of the variation (Figure 7). For the POUND 7 × UF273 Type 1 population, the average percentage of frosty pod infection was even lower, 0.3%, and no significant association was observed.

## Discussion

The use of genetic markers to increase breeding program efficiency remains limited in tropical tree crops. In addition to increasing yield, integrating disease resistance alleles remains a high priority for many crop species. In cacao, black pod and frosty pod disease are major obstacles to sustainable production. Although individuals with resistance to either black or frosty pod disease have been identified, efforts to consolidate both types of disease resistance and improved productivity into a single genetic pool remain limited. Here, we performed replicated evaluation of 148 superior cacao clones to identify individuals with high yield and resistance to frosty and black pod diseases. The individuals in the clonal trial had varying degrees of relatedness, with 29 diverse individuals from the germplasm collection and 119 crosses, including 19 full-sib families and several half-sib relationships. In general, the pedigrees and phylogenetic clustering of clones were in good agreement; only eight clones displayed a potential incorrectly labeled father.

### Phenotypic effect of ancestry

Using high-density markers, the clones and their phenotypic distributions were characterized relative to the proportion of their ancestry derived from cacao genetic groups. Amelonado and Nacional were the most represented genetic groups in the trial, which in the case of Nacional is consistent with the frequent use of UF 273 Type 1, UF 273 Type 2 and UF 712 as common parents, which have Nacional proportions of 0.46, 0.59, and 1, respectively. The negative correlation between the proportion of Nacional ancestry and the proportion of frosty pod infection is consistent with previous observations of sympatry between the Co-Wes genetic group of *M. roreri* (Phillips-Mora et al., 2007) isolated in western Colombia, central Ecuador, and Central America and the cacao Nacional genetic group (Motamayor et al., 2008). Natural selection may have enriched Nacional cacao subpopulation with resistance alleles against *M. roreri*. Contrary to frosty pod resistance, we observed a positive correlation between Nacional ancestry and the proportion of black pod infection, consistent with the susceptibility of both UF parents to black pod disease. In addition, the negative correlation between Marañon ancestry and the proportion of black pod disease is consistent with previous reports of high proportions of resistant and moderately resistant clones from that population (Iwaro et al., 2006). In terms of productivity, a significant positive correlation between yield and ancestry was observed for Nanay ancestry, with most of the high-yield clones corresponding to crosses between POUND 7 and UF 273. Criollo ancestry, which is generally associated with superior confectionary properties, displayed a negative correlation with healthy pod count and yield. These results highlight the importance of recombining disease resistance, superior processing qualities, and high productivity by leveraging the unique characteristics of cacao genetic groups.

### Phenotypic selection

An important goal of this work was to identify clones with a low incidence of black pod and frosty pod diseases. For frosty pod, we observed high disease pressure and high heritability for the proportion of pods with disease symptoms (0.79 and 0.73) at the La Lola farm, located at sea level in Costa Rica. In the QTL mapping population, located in the same country at the La Montana site (600 m above sea level), disease pressure was significantly lower, and susceptible controls showed only sporadic symptoms of disease during the trial. Interestingly, although both sites are in relative proximity, the greatest environmental difference is altitude, which could affect the distribution of the pathogen. For black pod, evaluated at La Lola, the disease pressure was lower, and the transformed count and proportion of pods with disease showed lower heritability than the proportion of pods with frosty pod.

When considering the fitted values for both diseases and yield, of the top 10% of the clones with the lowest frosty pod disease proportion, only CATIE R5, CATIE R6, and CATIE R58 were also among the highest yielding clones. For black pod, the equivalent set included clones CATIE R6 and CATIE R73. The highest-yielding clone was CATIE R92; however, CATIE R6 ranked among the top 10% for yield, as well as for frosty pod and black pod disease resistance, despite having a slightly lower yield than CATIE R92. Both clones have annotated UF 273 as a common parent; CATIE R 92 and CATIE R6 have POUND 7 and PA 169 as the other parent respectively. Clones UF 273 Type 1 and UF 273 Type 2 are very closely related, they belong to the UF series developed by the United Fruit company and both are moderately susceptible to black pod but resistant to frosty pod disease. Clone POUND 7 was collected by F. J. Pound in 1942 along the Nanay river, a tributary of the Amazon River in Peru, and it complements the disease traits of UF 273 clones, displaying resistance to black pod and susceptibility to frosty pod. Finally, clone PA 169 was collected in Peru in the Parinari district of the Loreto province, at Rio Marañon. Similar to UF 273 Type 1, this clone displays resistance to frosty pod. These results confirm the limited number of allele donors used in breeding for genetic resistance to frosty and black pod diseases.

### Rootstock effect

In addition to productivity and disease resistance, we characterized the effect of rootstock choice on the productivity of the scion. Significant rootstock effects have been documented in many other tree crops (Warschefsky et al., 2016). Although some studies have investigated the rootstock effect in cacao (Yin, 2004), the effect of rootstock choice on yield remains largely uncharacterized in cacao plantations worldwide. To generate rootstock seedlings in our trial, we obtained open pollinated pods from clones SPA 9, IMC 67, EET 400, UF 613, and PA 121. Using the productivity data, we observed a significant effect of rootstock on scion yield, with PA 121 and IMC 67 having a negative effect on the total number of healthy pods and EET 400 and UF 613 having a small positive effect on total pod count relative to SPA 9. Although compared with previous work (Yin, 2004) that controlled both maternal and paternal contributions, we were only able to test the maternal effect of seedling rootstock. Despite this, both studies show a significant effect of rootstock on productivity. A better understanding of rootstock effects and a potentially higher homogeneity of clonal productivity may be achieved by implementing and testing the performance of asexually propagated rootstock.

### Genetic mapping, genomic prediction, and differential expression

The GWA revealed significant associations for frosty pod and pod color. For pod color, the most significant marker was located at the TcMYB113 gene on chromosome 4, consistent with previous work (Motamayor et al., 2013). This result shows the potential for detecting low-complexity traits with high resolution in breeding populations of similar characteristics. Furthermore, the markers consistently associated with pod color can be used directly in marker assisted selection. In contrast to pod color, and unlike other single resistance genes, both frosty and black disease traits exhibited quantitative trait variation. From the GWA, six sites were significantly associated only with frosty pod disease at a false discovery rate threshold of 0.05 and located in chromosomes 4,5,6, and 10. The significantly associated markers do not overlap with previously reported QTL (Brown et al., 2007; Lanaud et al., 2009), which could be due to different alleles segregating in our population of study and previous analyses. The lack of association for the other traits analyzed could be due to multiple factors. In the case of yield, its highly quantitative nature would require a much higher number of individuals in order to detect significant association. In the case of the diseases, under the conditions of natural disease pressure black pod related traits displayed low levels of phenotypic variance and low estimates of heritability relative to frosty pod, which given the small sample size could have affected the statistical power to detect significant association. Finally, in a sample with high levels of recent admixture between individuals, the observed significant effect of ancestry on phenotypic distribution may have further affected the ability to detect association, with GWA models accounting for relatedness and structure also potentially accounting for phenotypic variance. In the linkage mapping population, a single QTL was observed on chromosome 5 for frosty pod disease, overlapping with the GWA results. Despite generally exhibiting higher statistical power, linkage mapping has lower resolution, limited by the very recent recombination of a mapping population. In our case, low phenotypic variance for the linkage mapping population produced by low disease incidence at the experimental site may have affected our power to detect all QTL underlying frosty pod disease resistance.

In contrast to the mapping results, we observed consistent prediction accuracies within traits across marker densities in the genomic selection and modest increases in accuracy with increases in the number of SNPs. This effect is probably due to the high LD in the samples due to limited recombination and admixture during recent breeding efforts. LD has been explored in other cacao populations, and although diversity panels with higher ancestral recombination may be better suited for GWAS (Stack et al., 2015), recently admixed populations may be better suited for genomic prediction, with much more effective selection for quantitative traits. In our analyses, the trait with the highest observed prediction accuracy was the proportion of frosty pod infection, a quantitative trait in our population, reaching prediction accuracies of 0.60 with 90 markers and 0.67 with all markers. As expected, most traits displayed prediction accuracies slightly lower than the heritability estimates except for black pod, which displayed accuracies higher than our heritability estimate; this effect is probably a product of low phenotypic variance due to low natural disease pressure in our experiment. Yield, a high-complexity phenotype, displayed a good prediction accuracy of 0.48 using all markers. The lowest prediction accuracy, 0.37, was observed for pod index. These results indicate that in breeding populations of heterozygous and recently related individuals, mapping approaches can be used for low complexity traits like pod color cacao, or in other species single gene disease resistance, however genomic selection for quantitative traits remains highly effective relative to mapping underlying QTL. In other words, for traits with high complexity incorporating all markers into a single random effect in a linear mixed model provides a powerful tool for accurately predicting phenotype and therefore performing selection, even in the absence of significant association at each individual causative loci. In crops like cacao, which displays significant difficulty in crossing due partly to its complex self-incompatibility, and which also contains large numbers of off-types in breeding collections globally, genomic selection also has the advantage of better representing relatedness without assumption of true parentage.

In addition to genome wide association, differential expression analysis can help unveil loci involved in response to experimental treatments. Two of the parents in the clonal trial showing contrasting disease resistance for frosty and black pod diseases, UF 273 Type 1 and POUND 7, were used for differential expression analysis in pod inoculation experiments. Two different statistical approaches were implemented and showed consistent patterns of differential expression, and for both methods, the differentially expressed genes were only significant for the cultivar effect and not the inoculation treatment. The lack of significantly differentially expressed genes in response to inoculation was probably due to sampling of tissues occurring too early during the pathogen cycle. Additional experiments with later sampling during fungal penetration may help elucidate the underlying resistance response. Nevertheless, across two analyses pipelines 35 genes showed consistent differential expression in pods between UF 273 and POUND 7, and of those four were annotated as potentially involved in disease resistance. Of the differentially expressed genes identified by EdgeR/Bioconductor, five were contained within 100 kb of genome wide association results: three unknown function protein, and two proteins annotated as GroES-like zinc-binding dehydrogenase family protein and Zinc-binding alcohol dehydrogenase family protein. Together, all the differentially expressed genes are good candidates for follow up studies that could help understand some of the key differences between clones UF 273 and POUND 7.

Understanding the genetic basis of improved productivity and disease resistance can advance the progress of selection in breeding programs. By performing multi-year evaluations on a set of diverse clones with differential expression analysis, we identified markers and models that can be immediately applied to predict disease and yield traits in related cacao germplasms. Notably, the plants in our study had relatively low yields due to implementation of traditional farm management and the young age of the plants compared with high-intensity plantation systems, suggesting that the full potential of the clones was not completely characterized. From a genetic perspective, the presence of several full-sib relationships in the pedigree of the clones translates to lower resolution of associations, as LD among full- and half-sibs is high compared with other association populations. Despite these limitations, the relatively high genomic selection predictive ability for all traits shows that selection using a low number of markers can help accelerate the cacao breeding cycle compared with linkage mapping followed by marker-assisted introgression. Some recommendations for a genomic-assisted breeding program derived from this work would be to conduct a multi-location evaluation of improved material to quantify genotype-by-environment effects and obtain better disease resistance information from locations with the most disease pressure. Careful recording of the rootstock maternal source or the use of asexually propagated rootstocks may also aid in understanding the effect of the interaction between rootstock and scion on productivity. Evaluation in high-intensity agricultural systems would also help characterize the full production potential of new clones. Additional analyses of expression at later stages of fungal infection would enable the identification of genes responsible for disease resistance. The novel use of GWA and genomic selection in this study highlights the significant opportunities for the potential application of genomics in tropical crops for sustainable improvement of agricultural production.

## Materials and methods

### Population of clonal cultivars

A total of 148 individuals were selected due to their superior performance. Selection was performed based on previous yield and disease resistance data (Phillips et al., 2009) on frosty pod and black pod diseases. The sample contained 35 individuals from the germplasm collection and 121 selections from crosses representing 29 diverse parents (Supplementary Table 1). The most common parent (75 times) of the selected individuals was UF 273 Type 1, a clone frequently used in cacao breeding programs for its resistance to frosty pod. The individuals were clonally propagated, with 25 clones derived for each of superior individual. Clones were propagated via grafting using open-pollinated seedlings as the rootstock. The maternal sources of the seedlings used as rootstock were selected for resistance to *Ceratocystis* wilt due to the biological spread of *Ceratocystis* wilt disease to rootstocks through attacks on their trunks by Ambrosia beetles of the genus *Xyleborus* (Engelbrecht et al., 2007). The five maternal sources of seeds used as rootstock were SPA 9, IMC 67, EET 400, UF 613, and PA 121. Phenotypic data was collected for 8 years.

### Field evaluation conditions

Yield evaluation was conducted at a location with a high endemic prevalence of black pod and frosty pod diseases. The trial was established by the CATIE Cacao Breeding Program in June–July 2006 in a 4-hectare area at the La Lola farm. This farm is on the Atlantic Coast of Costa Rica, located 40 meters above sea level at 10° 06′ N latitude and 83° 23′ W longitude. Most of the farm's soil (69%) consists of silty clay, and the remainder comprises 21% coarse sand and 10% sandy clay. The evaluation was performed in an agroforestry system with natural rainfall, application of 150 g of granular fertilizer formula 18-5-15 every 3 months, and manual pruning performed with a broad blade once or twice a year. The plants were planted in a completely randomized design, with 2.5 m spacing between plants. Temporal shade was provided by banana plants (*Musa* sp.) planted with 5-m spacing, and permanent shade was provided by the legume *Gliricidia sepium* at 7.5-m spacing. Border cacao plants were set up around the field, within the field around two rivers, and around a road contained within the field. Plants that died during the experiment were replaced with new grafts to provide an even canopy structure; however, these individuals were excluded from the analyses.

### Genotypic data

For genotyping, the parallel objectives were to characterize the presence of off-types in the field and to generate high-density markers for the true-to-type individuals. Off-types are a widespread problem in cacao breeding programs due to human sample error and biological phenomena such as rootstock escape. For each clone, two leaves were collected from three mature plants on the field. DNA was extracted from all leaves using a ZR-96 Plant/Seed DNA Kit (Zymo Research) and genotyped using Fluidigm. Using a majority rule, the most frequent clonal type was selected for high-density genotyping. To generate a large number of markers for likely true-to-type individuals, the most frequent clonal type was genotyped using a high-density SNP microarray (Livingstone, et al., in review). Three leaves from each tree were collected again, and genotyping was performed by LGC Genomics using Kompetitive Allele Specific PCR (KASP™) (http://www.lgcgenomics.com). After quality control (>80% sites), 3,733 individual plants contained a genotypic profile for 90 SNP markers. Using the 15 k SNP Chip as a reference, a 6.88% off-type rate was observed, with 90% of clones having three or fewer off-type plants. Off-type plants were removed from the analyses.

### Phenotypic data

Phenotypic data, in the form of pod counts, were collected monthly for each tree from May 2007 to April 2015 for a total of 394,126 pods counted. During the experiment, differences in plant phenology were observed according to their position relative to the two rivers; thus, three blocks representing three micro-environments were defined for the analysis. The phenotypic information collected included the number of mature pods, the number of healthy pods, and the number of pods with symptoms of black pod or frosty pod infection. To provide a better fit to a normal distribution, a square-root transformation was performed for the number of healthy pods, as well as for those with black or frosty pod disease symptoms. In addition, the proportion of diseased pods relative to the total was estimated as a derived statistic for disease resistance. Additional data on pod color and pod index were obtained from a previous characterization of the evaluated clones (Pérez Zú-iga, 2009). Pod color is controlled by a single large effect locus, making it a good positive control for association analyses; pod index is defined as the number of pods required to produce 1 Kg of fermented dry bean weight and therefore represents an important target for selection in breeding programs. In addition, the pod index was used to estimate the yield per hectare as the number of healthy pods per year divided by the pod index, times 1,111 plants per hectare, which corresponds to the typical cacao planting density.

The data for all years was analyzed together for the true-to-type plants using a mixed linear model. The model used in the data analysis was:

$$y_{ijk}=\mu +B_{i}+Y_{j}+B_{i}*Y_{j}+C_{k}+\epsilon_{ijk}$$

where *y*_*ijk*_ is the number or proportion of pods (healthy, with black pod or frosty pod disease symptoms) produced per tree per year, μ is the overall mean, *B*_*i*_ is the random effect of blocks, *Y*_*j*_ is the random effect of year, *B*_*i*_ * *Y*_*j*_ is the random block-by-year interaction, *C*_*k*_ is the random clone effect, and ϵ_*ijk*_ is the residual error, with $\epsilon_{ijk}\sim N\left(0,\sigma_{\epsilon }^{2}\right)$. For the analyses, only phenotypic data collected from 2008 onward were included.

In addition to the covariates above, data were available for 61% of the plants to match each scion with the maternal source of the corresponding rootstock. Therefore, an analysis of variance table was generated (Supplementary Table 4) for a linear model fitted to test the significance of the rootstock effect on productivity. The fitted model was:

$$y_{ij}=\mu +B_{i}+R_{j}+\epsilon_{ij}$$

where *y*_*ij*_ is the number of healthy pods, *B*_*i*_ is the main effect of block, *R*_*j*_ is the main effect of the rootstock and ϵ_*ij*_ is the residual error, with $\epsilon_{ijk}\sim N\left(0,\sigma_{\epsilon }^{2}\right)$.

### Structure analysis

The proportion of membership to each of the 10 cacao genetic groups was estimated using Admixture software (Alexander et al., 2009). A supervised admixture analysis was performed using individuals with over 85 proportion ancestry from the cacao ancestral groups (Motamayor et al., 2008). The resulting proportions of ancestry were used in an analysis of variance to establish the effect of ancestral population on phenotypic variation. The model was fitted using the fitted values for yield, number of healthy pods, proportion of pods with black or frosty pod disease, and pod index as the response variables. The explanatory variables were the proportions of ancestry for all individuals. Significance from the *F*-test was established at a 0.05 threshold after applying the Bonferroni adjustment for multiple testing and the regression lines for each explanatory variable are provided in Supplementary Figures 4–7, Supplementary Table 6.

### Genome-wide association and genomic selection

To identify the loci underlying the phenotypes, GWA was performed using a linear mixed model (Zhang et al., 2010) using TASSEL version 5 (Bradbury et al., 2007). In the model, markers were filtered for (MAF) > 0.05 yielding a total of 10,432 SNPs. The additive relationship matrix was estimated using the scaled method (Endelman and Jannink, 2012) as implemented in TASSEL. A mixed linear model was fitted for each phenotype using the BLUPs as the response variables, the membership coefficients as the fixed effects, and the additive relationship matrix as a random effect. Principal Component Analysis was performed using TASSEL with the same filtered marker set.

For genomic selection, the prediction accuracies were tested for a range of increasing marker densities. Genomic selection was performed with the entire marker set and with random subsampling of the genotypes to estimate the kinship matrices. The lowest marker density was 10 sites (1 on each chromosome), and higher densities consisted of random SNPs in increments of 500 up to 10,000 SNPs. Prediction accuracy was estimated by performing 5-fold cross validation 50 times with GAPIT software, using the method proposed by VanRaden to estimate kinship matrices. To estimate the upper limit of the prediction accuracies, heritability was estimated as the proportion of variance explained by relatedness (Speed et al., 2012) using LDAK software (Speed and Balding, 2014), and the relatedness matrix was estimated from all markers with MAF > 0.05.

### Differential expression during early frosty pod infection

Comparing divergent expression between genes relative to specific treatments can help unveil the molecular mechanism underlying the response to treatment. Based on laboratory conidial germination observations, cacao pod samples were taken at 8,24,48 h after inoculation to capture the differentially expressed genes corresponding to the initial interaction between the plant and the germinating fungal conidia. For each clone at each time-point, one control and three frosty pod inoculation biological replicates were collected. In addition, two technical replicates were collected at each time-point for one biological replicate of frosty pod inoculation.

A total of 27 pod samples were sequenced. The polyA fraction was isolated from total RNA, which was used to prepare a coding RNA-Seq library. Each library was uniquely barcoded with 7-mer oligonucleotides. Five barcoded RNA-Seq libraries were pooled in one lane of a HiSeq 2500 flow cell for 100-bp paired-end sequencing. RNA-Seq reads from each sample were mapped to the cacao Matina v1.1 reference genome (Motamayor et al., 2013) and transcriptome using Bowtie (Langmead et al., 2009) and TopHat (Trapnell et al., 2009) and assembled into gene- and transcription-level summaries using htseq-count (Anders et al., 2015). Mapped data were subjected to quality control with FastQC (http://www.bioinformatics.babraham.ac.uk/projects/fastqc/) and RNA-SeQC (DeLuca et al., 2012) to ensure sufficient library diversity and transcript coverage, as well as to assess potential contamination with ribosomal RNAs. The read counts mapped on the 45,600 cacao gene models vary from 2,630,962 to 37,694,247 counts per sample (Supplementary Figure 9). Between 20,217 and 25,927 genes had no reads mapped to them in a given sample.

The analysis using RoDEO software (Haiminen et al., 2014) was performed as follows. First, given that the three smaller samples have considerably different sequencing depths, careful scaling was needed to achieve comparable read counts between samples. Read counts were processed for differential expression analysis using RoDEO software (Haiminen et al., 2014), and samples with highly variable sequencing depths were scaled for comparison. RoDEO is robust in the presence of noise and limited or missing replicates, and in this case, samples with very different numbers of mapped reads. To establish thresholds for differential expression, the RoDEO differential expression scores of the technical and biological replicates were compared. MRoDEO DE scores up to 7 and 10 were observed in the technical and biological replicates, respectively (Supplementary Figure 10) but inoculation had no significant effect on gene expression.

The second method of differential expression analysis using EdgeR/Bioconductor was performed as follows. Raw fragment (i.e., paired-end read) counts were used as input for gene expression differential analysis with the Bioconductor Limma package (Ritchie et al., 2015) after multiple filtering steps to select for genes with expression levels above 1 FPKM (fragments per kb per million reads) in at least 50% of samples, and to remove genes with <50 total reads across all samples or of <200 nucleotides in length. Normalization factors were computed on the filtered data matrix using the weighted trimmed mean of M-values (TMM) method (Robinson and Oshlack, 2010), followed by voom mean-variance transformation (Law et al., 2014) in preparation for Limma linear modeling. Baseline differences between POUND 7 and UF 273 Type 1 were estimated between cultivar controls using general linearized model with separate parameters for time and cultivar effects. For the baseline differences between time points, expression differences between any two time points for each set of cultivar controls was assessed. Finally, differences in response to infection in POUND 7 and UF 273 were performed using contrast between M. roreri and mock infection for each time point and cultivar. eBayes adjusted *P*-values were corrected for multiple testing using the Benjamin-Hochberg (BH) method and used to select genes with significant expression differences (*q* < 0.01 Supplementary Table 5).

### Mapping quantitative trait loci

The mapping populations consist of the previously reported (Brown et al., 2007) crosses of POUND 7 × UF 273 Type 1 and POUND 7 × UF 273 Type 2, with 180 individuals and 68 individuals, respectively. The trials were established at the CATIE Turrialba experimental farm on the Atlantic coast of Matina, Costa Rica (lat. 10°06′N, 83°23′W, 40 m.a.s.l) in 1998. Eleven years of data were collected for the number of healthy and Monilia-infected pods per individual. Both populations were genotyped with an Illumina Infinium 6K chip (Livingstone et al., 2015). Non-informative markers were removed, and the remaining markers were used to construct a genetic map using JoinMap 4.1 with the maximum likelihood estimation (MLE) method, and 2,705 segregating markers were retained for genetic mapping. The proportion of Monilia-infected pods to total pods harvested was calculated per year and averaged across all years. Quantitative-trait loci for the yearly and averaged proportions of Monilia-infected pod phenotypes were mapped with MapQTL 6 (interval-mapping). A significant threshold for the test statistic (LOD) was determined by using resampling 1,000 times without replacement of the phenotype values while holding genome-wide marker data fixed in order to test the null hypothesis of no QTL. After each iteration, the maximum test statistic is recorded and a frequency distribution from the iterations is obtained (Churchill and Doerge, 1994). The value of the LOD score at which the relative cumulative count is 0.95 (*p*-value = 0.05) was 4.2 for the trait analyzed therefore LOD score values >4.2 were considered significant for frosty pod percentages.

## Author contributions

JR analyzed data and wrote the manuscript. WP-M conceived the experiment, analyzed data and wrote the manuscript. AA-L collected and analyzed data. AM-Q collected and analyzed data. NH analyzed data and wrote the manuscript. GM analyzed data and wrote the manuscript. DL generated, analyzed data and wrote the manuscript. HvB analyzed data and wrote the manuscript. DK conceived the experiment, generated data and wrote the manuscript. LP conceived the experiment, analyzed data. AK conceived the experiment, generated data and wrote the manuscript. JM conceived the experiment, analyzed data and wrote the manuscript

### Conflict of interest statement

The authors declare that the research was conducted in the absence of any commercial or financial relationships that could be construed as a potential conflict of interest.
