# Supplementary material for: Application of Genome Wide Association and Genomic Prediction for Improvement of Cacao Productivity and Resistance to Black and Frosty Pod Diseases
Source: Front Plant Sci. 2017 Nov 14;8:1905. doi: 10.3389/fpls.2017.01905 (PMC5694496; doi:10.3389/fpls.2017.01905)
Supplement: Supplementary file 1 [file DataSheet1.docx]

Supplementary Material

Application of genome wide association and genomic prediction for improvement of cacao productivity and resistance to black and frosty pod diseases

**J. Alberto Romero Navarro^1^, Wilbert Phillips-Mora^2^, Adriana Arciniegas-Leal^2^, Allan Mata-Quirós^2^, Niina Haiminen^3^, Guiliana Mustiga^1^, Donald Livingstone III^1^, Harm Van bakel^4^, David N Kuhn^5^, Laxmi Parida^3^, Andrew Kasarskis^4^, Juan Carlos Motamayor^1^***

^1^ Mars Incorporated, Miami, FL, USA

^2^ Tropical Agricultural Research and Higher Education Center, Turrialba, Costa Rica

^3^IBM T J Watson Research Center, Yorktown Heights, New York, USA

^4^Icahn Institute of Genomics and Multiscale Biology, Icahn School of Medicine at Mount Sinai, New York, NY, USA

^5^United States Department of Agriculture-Agriculture Research Service, Subtropical Horticulture Research Station, Miami, FL 33158, USA

*** Correspondence:**Juan Carlos Motamayor
juan.motamayor@effem.com

# Supplementary Data

Supplementary Material should be uploaded separately on submission. Please include any supplementary data, figures and/or tables.

Supplementary material is not typeset so please ensure that all information is clearly presented, the appropriate caption is included in the file and not in the manuscript, and that the style conforms to the rest of the article.

# Supplementary Tables

**Supplementary Table 1**. Phenotypic characteristics and origin of clones used in the experiment as parents or for the evaluation.

| **Clone** | **Clone use** | **Black Pod**  **Resistance** | **Frosty Pod**  **Resistance** | **Yield** | **Collection/Country** |
| --- | --- | --- | --- | --- | --- |
| ARF 22 | Parent | HIGH | LOW | MEDIUM | breeding clone CATIE/ Costa Rica |
| ARF 37 | Parent | HIGH | LOW | MEDIUM | breeding clone CATIE/ Costa Rica |
| CATIE 1000 | Parent | HIGH | LOW | LOW | breeding clone CATIE/ Costa Rica |
| SCA 6 | Parent | HIGH | LOW | LOW | wild clone/ Peru |
| CCN 51 Type 2 | Parent | LOW | MEDIUM | HIGH | Offtype CCN 51 |
| EEG 25 | Parent | LOW | LOW | UNKNOWN | farmer selection/ Brazil |
| EEG 27 | Parent | LOW | UNKNOWN | UNKNOWN | farmer selection/Brazil |
| EET 75 | Parent | LOW | HIGH | LOW | breeding clone INIAP/ Ecuador |
| ICS 1 | Parent | LOW | LOW | MEDIUM | breeding clone Imperial College/ Trinidad |
| PA 121 | Parent | LOW | UNKNOWN | LOW | wild clone/ Peru |
| PA 16 | Parent | LOW | UNKNOWN | UNKNOWN | wild clone/ Peru |
| SIC 813 | Parent | LOW | UNKNOWN | UNKNOWN | breeding clone CEPLAC/ Brazil |
| UF 668 | Parent | LOW | LOW | MEDIUM | breeding clone United Fruit Company/ Costa Rica |
| ARF 6 | Parent | MEDIUM | MEDIUM | MEDIUM | breeding clone Hershey/ Belize |
| CC 124 | Parent | MEDIUM | UNKNOWN | UNKNOWN | open pollinated clone CATIE/ Costa Rica |
| CC 252 | Parent | MEDIUM | MEDIUM | LOW | breeding clone CATIE/ Costa Rica |
| EET 95 | Parent | MEDIUM | MEDIUM | MEDIUM | breeding clone INIAP/ Ecuador |
| ICS 8 | Parent | MEDIUM | UNKNOWN | UNKNOWN | breeding clone Imperial College/ Trinidad |
| MA 13 | Parent | MEDIUM | LOW | UNKNOWN | farmer selection/ Brazil |
| SIC 433 | Parent | MEDIUM | UNKNOWN | UNKNOWN | breeding clone CEPLAC / Brazil |
| TREE 81 | Parent | MEDIUM | MEDIUM | MEDIUM | breeding clone CATIE/ Costa Rica |
| CAUCASIA 34 | Evaluation | UNKNOWN | MEDIUM | MEDIUM | breeding clone Compañía Nacional de Chocolates/ Colombia |
| CAUCASIA 37 | Evaluation | UNKNOWN | MEDIUM | MEDIUM | breeding clone Compañía Nacional de Chocolates/ Colombia |
| CAUCASIA 39 | Evaluation | UNKNOWN | MEDIUM | MEDIUM | breeding clone Compañía Nacional de Chocolates/ Colombia |
| CAUCASIA 43 | Evaluation | UNKNOWN | MEDIUM | MEDIUM | breeding clone Compañía Nacional de Chocolates/ Colombia |
| EET 605 | Evaluation | UNKNOWN | MEDIUM | MEDIUM | breeding clone INIAP/ Ecuador |
| EET 610 | Evaluation | UNKNOWN | MEDIUM | MEDIUM | breeding clone INIAP/ Ecuador |
| FHIA FCS A2 | Evaluation | UNKNOWN | LOW | LOW | breeding clone FHIA/ Honduras |
| ICS 43RED | Evaluation/  Parent | UNKNOWN | MEDIUM | LOW | Offtype/ Costa Rica |
| PA 120 | Evaluation | UNKNOWN | MEDIUM | LOW | wild clone/ Peru |
| PMCT 46 | Evaluation | HIGH | MEDIUM | LOW | farmer selection/ Nicaragua |
| POUND 7 | Evaluation/  Parent | HIGH | LOW | MEDIUM | wild clone/ Peru |
| SNK 12 | Evaluation | HIGH | MEDIUM | LOW | breeding clone IRAD/ Cameroom |
| CAP 34 | Evaluation | LOW | LOW | MEDIUM | breeding clone Ecuador/INIAP |
| CARMELO 2 | Evaluation | LOW | LOW | MEDIUM | breeding clone Private / Mexico |
| CAUCASIA 47 | Evaluation | LOW | MEDIUM | MEDIUM | breeding clone Compañía Nacional de Chocolates/ Colombia |
| NAL 1 A13 | Evaluation | LOW | HIGH | LOW | seed progeny/ Ecuador |
| NAL 2 A26 | Evaluation | LOW | MEDIUM | LOW | seed progeny/ Ecuador |
| PA 107 | Evaluation | LOW | MEDIUM | LOW | wild clone/ Peru |
| PA 169 | Evaluation/  Parent | LOW | HIGH | LOW | wild clone,/Peru |
| PMCT 58 | Evaluation | LOW | MEDIUM | MEDIUM | breeding clone CATIE/ Costa Rica |
| PORCELANA 3 | Evaluation | LOW | MEDIUM | LOW | field clone/ Venezuela |
| UF 12 | Evaluation | LOW | MEDIUM | MEDIUM | breeding clone United Fruit Company/ Costa Rica |
| UF 712 | Evaluation/  Parent | LOW | HIGH | LOW | breeding clone United Fruit Company/ Costa Rica |
| CC 137 | Evaluation/  Parent | MEDIUM | MEDIUM | MEDIUM | breeding clone CATIE/ Costa Rica |
| GU 128 N | Evaluation | MEDIUM | MEDIUM | MEDIUM | wild clone/ French Guiana |
| HY 2714184 | Evaluation | MEDIUM | MEDIUM | MEDIUM | breeding clone USDA-TRAS/ Puerto Rico |
| ICS 95 T1 | Evaluation/  Parent | MEDIUM | MEDIUM | MEDIUM | breeding clone Imperial College/ Trinidad |
| NAL 1 A14 | Evaluation | MEDIUM | MEDIUM | LOW | seed progeny/ Ecuador |
| NAL 2 A27 | Evaluation | MEDIUM | MEDIUM | MEDIUM | seed progeny/ Ecuador |
| SC 24 | Evaluation | MEDIUM | MEDIUM | LOW | wild clone/ Brazil |
| UF 273 | Evaluation/  Parent | MEDIUM | HIGH | MEDIUM | breeding clone CATIE/ Costa Rica |
| YUCA | Evaluation | no | LOW | LOW | wild clone/ Mexico |

**Supplementary Table 2**. Clones included in the trial, and corresponding pedigree and proportion of ancestry relative to the 10 cacao ancestral subpopulations. Subpopulation names are abbreviated and correspond to Marañon, Nacional, Amelonado, Criollo, Contamana, Nanay, Guiana, Curaray, Iquitos, and Purus

| **Clone** | **Pedigree** | **Mar.** | **Nac.** | **Ame.** | **Cri.** | **Cont.** | **Nan.** | **Gui.** | **Cur.** | **Iqu.** | **Pur.** |
| --- | --- | --- | --- | --- | --- | --- | --- | --- | --- | --- | --- |
| CAP 34 | CAP 34 | 0 | 0.31 | 0.47 | 0.21 | 0 | 0 | 0 | 0 | 0 | 0 |
| CATIE R10 | UF 273 x CC 137 | 0.07 | 0.24 | 0.46 | 0.19 | 0 | 0 | 0 | 0.02 | 0.01 | 0 |
| CATIE R101 | Pound 7 x UF 273 | 0 | 0.41 | 0.12 | 0 | 0 | 0.27 | 0 | 0 | 0.21 | 0 |
| CATIE R102 | Pound 7 x UF 273 | 0 | 0.33 | 0.32 | 0.05 | 0 | 0.2 | 0 | 0.01 | 0.09 | 0 |
| CATIE R103 | Pound 7 x UF 273 | 0 | 0.5 | 0.16 | 0 | 0 | 0.34 | 0 | 0 | 0 | 0 |
| CATIE R104 | Pound 7 x UF 273 | 0 | 0.17 | 0.18 | 0.07 | 0.04 | 0.3 | 0.04 | 0.03 | 0.14 | 0.03 |
| CATIE R105 | EET 95 x UF 273 | 0.04 | 0.26 | 0.43 | 0.06 | 0 | 0 | 0 | 0.07 | 0 | 0.15 |
| CATIE R106 | UF 668 x UF 273 | 0 | 0.09 | 0.53 | 0.31 | 0 | 0 | 0.01 | 0 | 0.06 | 0 |
| CATIE R107 | UF 668 x UF 273 | 0 | 0.17 | 0.36 | 0.4 | 0 | 0 | 0 | 0.07 | 0 | 0 |
| CATIE R108 | UF 273 x PA 16 | 0.27 | 0.23 | 0.22 | 0.1 | 0.04 | 0 | 0.1 | 0 | 0.04 | 0 |
| CATIE R109 | UF 273 x PA 16 | 0.41 | 0.29 | 0.14 | 0.03 | 0 | 0.07 | 0 | 0.06 | 0 | 0 |
| CATIE R11 | UF 273 x CC 137 | 0 | 0.19 | 0.51 | 0.17 | 0 | 0 | 0 | 0 | 0.1 | 0.02 |
| CATIE R110 | UF 273 x PA 16 | 0.4 | 0.33 | 0.17 | 0.08 | 0.02 | 0 | 0 | 0 | 0 | 0 |
| CATIE R111 | UF 273 x PA 16 | 0.4 | 0.26 | 0.05 | 0.04 | 0.01 | 0.07 | 0.01 | 0.1 | 0.02 | 0.04 |
| CATIE R112 | UF 273 x PA 16 | 0.31 | 0.4 | 0.13 | 0.08 | 0.08 | 0 | 0 | 0 | 0 | 0 |
| CATIE R113 | UF 273 xCATIE 1000 | 0 | 0.27 | 0.45 | 0.05 | 0.03 | 0.12 | 0 | 0.08 | 0 | 0 |
| CATIE R114 | UF 273 x MA 13 | 0 | 0.29 | 0.49 | 0.02 | 0 | 0.06 | 0 | 0.05 | 0.07 | 0.02 |
| CATIE R116 | UF 273 x MA 13 | 0.04 | 0.12 | 0.44 | 0.03 | 0 | 0.04 | 0 | 0.04 | 0.15 | 0.13 |
| CATIE R117 | UF 273 x EEG 25 | 0.07 | 0.38 | 0.5 | 0 | 0 | 0.04 | 0 | 0 | 0 | 0 |
| CATIE R118 | UF 273 x EEG 25 | 0.09 | 0.19 | 0.58 | 0.01 | 0 | 0.01 | 0 | 0.1 | 0 | 0.03 |
| CATIE R119 | UF 273 x ICS 1 | 0 | 0.28 | 0.45 | 0.21 | 0 | 0 | 0 | 0.05 | 0 | 0 |
| CATIE R12 | UF 273 x CC 137 | 0.11 | 0.22 | 0.43 | 0.22 | 0 | 0 | 0 | 0.01 | 0 | 0.01 |
| CATIE R120 | UF 273 x ICS 1 | 0 | 0.21 | 0.43 | 0.2 | 0.04 | 0 | 0 | 0.12 | 0 | 0 |
| CATIE R121 | UF 273 x ICS 1 | 0.44 | 0.31 | 0.17 | 0.06 | 0 | 0.02 | 0 | 0 | 0 | 0 |
| CATIE R122 | UF 273 x ICS 1 | 0 | 0.22 | 0.46 | 0.19 | 0.03 | 0 | 0 | 0.1 | 0 | 0 |
| CATIE R124 | UF 273 x PA 169 | 0.43 | 0.37 | 0.18 | 0.01 | 0 | 0 | 0 | 0.01 | 0 | 0 |
| CATIE R125 | UF 273 x PA 169 | 0.47 | 0.2 | 0.17 | 0.12 | 0.01 | 0 | 0 | 0.02 | 0 | 0.02 |
| CATIE R126 | UF 273 x PA 169 | 0.39 | 0.15 | 0.2 | 0.08 | 0 | 0 | 0.06 | 0.05 | 0 | 0.08 |
| CATIE R127 | UF 273 x PA 169 | 0.37 | 0.09 | 0.28 | 0.12 | 0.05 | 0 | 0 | 0 | 0.02 | 0.06 |
| CATIE R128 | UF 273 x PA 169 | 0.42 | 0.23 | 0.07 | 0.03 | 0 | 0 | 0.06 | 0.05 | 0.06 | 0.08 |
| CATIE R129 | UF 273 x PA 169 | 0.39 | 0.39 | 0.19 | 0 | 0 | 0 | 0 | 0.03 | 0 | 0 |
| CATIE R13 | UF 273 x Catie 1000 | 0 | 0.39 | 0.4 | 0 | 0.04 | 0.12 | 0 | 0.05 | 0.01 | 0 |
| CATIE R130 | UF 273 x PA 169 | 0.4 | 0.26 | 0.02 | 0 | 0 | 0.06 | 0.06 | 0.16 | 0.05 | 0 |
| CATIE R131 | UF 273 x PA 169 | 0.35 | 0.36 | 0.2 | 0.04 | 0.05 | 0 | 0 | 0 | 0 | 0 |
| CATIE R132 | UF 273 x PA 169 | 0.37 | 0.21 | 0.2 | 0.1 | 0.08 | 0 | 0.02 | 0 | 0.03 | 0 |
| CATIE R133 | UF 273 x PA 169 | 0.51 | 0.36 | 0.08 | 0.02 | 0.02 | 0.02 | 0 | 0 | 0 | 0 |
| CATIE R134 | UF 273 x PA 121 | 0.38 | 0.38 | 0.16 | 0.08 | 0 | 0 | 0 | 0.01 | 0 | 0 |
| CATIE R135 | UF 273 x SIC 433 | 0 | 0.32 | 0.59 | 0.08 | 0 | 0 | 0 | 0 | 0 | 0 |
| CATIE R136 | UF 273 x EEG 27 | 0 | 0.41 | 0.52 | 0 | 0 | 0.01 | 0 | 0 | 0 | 0.06 |
| CATIE R137 | UF 273 x ICS 8 | 0 | 0.23 | 0.52 | 0.12 | 0 | 0 | 0 | 0.02 | 0 | 0.1 |
| CATIE R138 | UF 273 x SIC 813 | 0 | 0.23 | 0.57 | 0 | 0 | 0 | 0.05 | 0.11 | 0.04 | 0.01 |
| CATIE R14 | UF 273 x Catie 1000 | 0 | 0.5 | 0.34 | 0.02 | 0.01 | 0.1 | 0 | 0 | 0.02 | 0 |
| CATIE R15 | UF 273 x Pound 7 | 0 | 0.24 | 0.22 | 0.06 | 0 | 0.37 | 0 | 0.01 | 0.09 | 0.01 |
| CATIE R17 | UF 273 x Pound 7 | 0.04 | 0.19 | 0.19 | 0.01 | 0 | 0.37 | 0 | 0.06 | 0.12 | 0.02 |
| CATIE R18 | UF 273 x Pound 7 | 0 | 0.4 | 0.12 | 0 | 0 | 0.26 | 0 | 0.04 | 0.14 | 0.05 |
| CATIE R19 | UF 273 x Tree 81 | 0 | 0.4 | 0.19 | 0.05 | 0 | 0.22 | 0 | 0 | 0.12 | 0.02 |
| CATIE R20 | UF 273 x Tree 81 | 0 | 0.22 | 0.38 | 0.06 | 0.07 | 0.09 | 0 | 0.01 | 0.16 | 0 |
| CATIE R21 | UF 273 x Tree 81 | 0.14 | 0.17 | 0.28 | 0.17 | 0.04 | 0 | 0 | 0 | 0.19 | 0.02 |
| CATIE R22 | UF 273 x Tree 81 | 0 | 0.37 | 0.23 | 0.03 | 0.04 | 0.18 | 0.01 | 0.03 | 0.11 | 0 |
| CATIE R23 | UF 273 x CCN 51 Type 2 | 0 | 0.46 | 0.28 | 0.18 | 0 | 0 | 0 | 0.08 | 0 | 0 |
| CATIE R24 | UF 273 x SCA 6 | 0 | 0.41 | 0.09 | 0 | 0.5 | 0 | 0 | 0 | 0 | 0 |
| CATIE R25 | UF 712 x UF 273 | 0 | 0.81 | 0.13 | 0 | 0 | 0 | 0 | 0 | 0.05 | 0.01 |
| CATIE R26 | UF 712 x CC 137 | 0 | 0.45 | 0.27 | 0.24 | 0 | 0 | 0 | 0.03 | 0.01 | 0 |
| CATIE R27 | UF 712 x CC 137 | 0 | 0.62 | 0.32 | 0 | 0 | 0.05 | 0.01 | 0 | 0 | 0 |
| CATIE R28 | UF 712 x CC 137 | 0 | 0.51 | 0.31 | 0.16 | 0.02 | 0 | 0 | 0 | 0 | 0 |
| CATIE R29 | UF 712 x CC 137 | 0 | 0.54 | 0.4 | 0.06 | 0 | 0 | 0 | 0 | 0 | 0 |
| CATIE R3 | UF 273 x MA 13 | 0 | 0.44 | 0.31 | 0 | 0.07 | 0 | 0 | 0.06 | 0.11 | 0 |
| CATIE R30 | UF 712 x CC 137 | 0 | 0.56 | 0.38 | 0 | 0.07 | 0 | 0 | 0 | 0 | 0 |
| CATIE R31 | UF 712 x CC 137 | 0 | 0.55 | 0.27 | 0 | 0.12 | 0.06 | 0 | 0 | 0 | 0 |
| CATIE R32 | UF 712 x CC 124 | 0 | 0.62 | 0.3 | 0 | 0.03 | 0.05 | 0 | 0 | 0 | 0 |
| CATIE R33 | UF 712 x CC 124 | 0 | 0.64 | 0.36 | 0 | 0 | 0 | 0 | 0 | 0 | 0 |
| CATIE R34 | UF 712 x CC 124 | 0 | 0.59 | 0.31 | 0 | 0.06 | 0.02 | 0.01 | 0 | 0 | 0 |
| CATIE R35 | UF 712 x CC 124 | 0 | 0.56 | 0.38 | 0 | 0.07 | 0 | 0 | 0 | 0 | 0 |
| CATIE R36 | UF 712 x CC 124 | 0 | 0.55 | 0.41 | 0 | 0.01 | 0.02 | 0 | 0 | 0 | 0 |
| CATIE R37 | UF 712 x Catie 1000 | 0 | 0.55 | 0.15 | 0 | 0.02 | 0.05 | 0 | 0.05 | 0.18 | 0 |
| CATIE R38 | UF 712 x Tree 81 | 0.02 | 0.58 | 0.13 | 0 | 0 | 0.11 | 0 | 0.01 | 0.14 | 0 |
| CATIE R39 | UF 712 x Tree 81 | 0.02 | 0.42 | 0.05 | 0.05 | 0.16 | 0.1 | 0.02 | 0 | 0.17 | 0 |
| CATIE R4 | UF 273 x Pa 169 | 0.44 | 0.2 | 0.08 | 0.06 | 0.03 | 0.04 | 0 | 0.04 | 0.02 | 0.09 |
| CATIE R40 | UF 712 x Tree 81 | 0 | 0.55 | 0.2 | 0 | 0.02 | 0.15 | 0 | 0.06 | 0.02 | 0 |
| CATIE R41 | UF 712 x Tree 81 | 0.04 | 0.47 | 0.19 | 0 | 0.04 | 0.06 | 0 | 0 | 0.21 | 0 |
| CATIE R42 | CC 252 x Pound 7 | 0 | 0.19 | 0.41 | 0.1 | 0 | 0.22 | 0 | 0 | 0.08 | 0.01 |
| CATIE R43 | CC 252 x Pound 7 | 0.02 | 0.02 | 0.2 | 0.14 | 0.02 | 0.34 | 0.03 | 0.02 | 0.14 | 0.06 |
| CATIE R44 | CC 252 x SCA 6 | 0 | 0 | 0.26 | 0.09 | 0.58 | 0.02 | 0 | 0.06 | 0 | 0 |
| CATIE R47 | ICS 95 x UF 273 | 0 | 0.43 | 0.33 | 0.24 | 0 | 0 | 0 | 0 | 0 | 0 |
| CATIE R48 | ICS 95 x UF 712 | 0.11 | 0.45 | 0.13 | 0.27 | 0 | 0 | 0 | 0.04 | 0 | 0 |
| CATIE R49 | ICS 95 x Pound 7 | 0 | 0 | 0.28 | 0.15 | 0 | 0.29 | 0.08 | 0 | 0.19 | 0 |
| CATIE R5 | UF 273 x Pa 169 | 0.5 | 0.24 | 0.1 | 0.09 | 0 | 0.03 | 0 | 0.03 | 0 | 0 |
| CATIE R50 | EET 75 x CC 252 | 0 | 0.35 | 0.41 | 0.1 | 0.14 | 0 | 0 | 0 | 0 | 0 |
| CATIE R52 | EET 75 x CC 137 | 0 | 0.39 | 0.39 | 0.22 | 0 | 0 | 0 | 0 | 0 | 0 |
| CATIE R54 | EET 75 x Catie1000 | 0 | 0.4 | 0.3 | 0.11 | 0.02 | 0.15 | 0 | 0 | 0.02 | 0 |
| CATIE R56 | EET 75 x Catie1000 | 0 | 0.47 | 0.27 | 0.04 | 0.07 | 0.14 | 0 | 0.01 | 0 | 0 |
| CATIE R57 | CC 137 x UF 273 | 0 | 0.33 | 0.47 | 0.15 | 0 | 0 | 0 | 0.05 | 0 | 0 |
| CATIE R58 | CC 137 x UF 273 | 0 | 0.21 | 0.5 | 0.23 | 0 | 0 | 0 | 0.07 | 0 | 0 |
| CATIE R6 | UF 273 x Pa 169 | 0.43 | 0.25 | 0.08 | 0.03 | 0 | 0.08 | 0 | 0.08 | 0.04 | 0.02 |
| CATIE R60 | CC 137 x SCA 6 | 0 | 0.05 | 0.28 | 0.16 | 0.51 | 0 | 0 | 0 | 0 | 0 |
| CATIE R61 | CC 137 x SCA 6 | 0 | 0.03 | 0.22 | 0.23 | 0.53 | 0 | 0 | 0 | 0 | 0 |
| CATIE R62 | Catie 1000 x CC 137 | 0 | 0.08 | 0.47 | 0.13 | 0.07 | 0.18 | 0 | 0.07 | 0 | 0 |
| CATIE R63 | Catie 1000 x CC 137 | 0 | 0.2 | 0.45 | 0.06 | 0.06 | 0.16 | 0 | 0 | 0 | 0.07 |
| CATIE R64 | CC 137 x SCA 6 | 0 | 0.05 | 0.27 | 0.17 | 0.51 | 0 | 0 | 0 | 0 | 0 |
| CATIE R65 | Tree 81 x ICS 95 | 0.24 | 0 | 0.24 | 0.32 | 0.01 | 0.02 | 0.01 | 0 | 0.14 | 0.01 |
| CATIE R66 | SCA 6 x UF 712 | 0 | 0.46 | 0 | 0 | 0.54 | 0 | 0 | 0 | 0 | 0 |
| CATIE R68 | ARF 22 x ARF 6 | 0 | 0 | 0.36 | 0.29 | 0 | 0.25 | 0 | 0 | 0.05 | 0.05 |
| CATIE R7 | UF 712 x CATIE 1000 | 0 | 0.54 | 0.16 | 0 | 0.06 | 0.11 | 0 | 0 | 0.13 | 0 |
| CATIE R70 | ARF 22 x UF 273 | 0 | 0.43 | 0.3 | 0.01 | 0 | 0.18 | 0 | 0.04 | 0 | 0.04 |
| CATIE R71 | CCN 51 Type 2 x PA 169 | 0.43 | 0.12 | 0.24 | 0.14 | 0 | 0.04 | 0.03 | 0 | 0 | 0 |
| CATIE R72 | PA 169 x ARF 6 | 0.45 | 0.02 | 0.3 | 0.2 | 0 | 0.01 | 0.02 | 0 | 0 | 0 |
| CATIE R73 | PA 169 x ARF 22 | 0.53 | 0 | 0.11 | 0 | 0 | 0.24 | 0 | 0 | 0.05 | 0.06 |
| CATIE R75 | PA 169 x ARF 22 | 0.32 | 0.07 | 0.26 | 0 | 0.03 | 0.17 | 0.06 | 0 | 0.04 | 0.05 |
| CATIE R76 | PA 169 x ARF 22 | 0.36 | 0 | 0.24 | 0 | 0 | 0.14 | 0.08 | 0 | 0.03 | 0.15 |
| CATIE R77 | PA 169 x ARF 22 | 0.48 | 0.04 | 0.16 | 0 | 0.02 | 0.15 | 0.05 | 0 | 0.11 | 0 |
| CATIE R78 | PA 169 x ARF 22 | 0.39 | 0.01 | 0.26 | 0 | 0.05 | 0.18 | 0 | 0 | 0.07 | 0.04 |
| CATIE R79 | PA 169 x ARF 22 | 0.42 | 0.06 | 0.32 | 0 | 0.03 | 0.15 | 0 | 0 | 0 | 0.02 |
| CATIE R8 | UF 273 x CC 137 | 0 | 0.35 | 0.39 | 0.24 | 0 | 0 | 0 | 0.03 | 0 | 0 |
| CATIE R80 | PA 169 x ARF 37 | 0.38 | 0.03 | 0.12 | 0 | 0.07 | 0.05 | 0.12 | 0 | 0.21 | 0.03 |
| CATIE R82 | UF 712 x ARF 37 | 0 | 0.48 | 0.31 | 0 | 0.04 | 0 | 0 | 0 | 0.15 | 0.02 |
| CATIE R85 | UF 712 x ARF 37 | 0.13 | 0.46 | 0.06 | 0 | 0.09 | 0 | 0.03 | 0.01 | 0.17 | 0.05 |
| CATIE R86 | UF 712 x ICS 43 | 0 | 0.44 | 0.19 | 0.1 | 0.13 | 0.03 | 0 | 0.09 | 0 | 0.02 |
| CATIE R87 | UF 712 x ICS 43 | 0.04 | 0.58 | 0.1 | 0.22 | 0 | 0.02 | 0.01 | 0 | 0 | 0.04 |
| CATIE R89 | Pound 7 x UF 273 | 0 | 0.32 | 0.24 | 0.03 | 0 | 0.22 | 0 | 0.02 | 0.18 | 0 |
| CATIE R9 | UF 273 x CC 137 | 0 | 0.27 | 0.36 | 0.3 | 0 | 0 | 0 | 0.06 | 0 | 0 |
| CATIE R90 | Pound 7 x UF 273 | 0 | 0.26 | 0.13 | 0 | 0 | 0.43 | 0 | 0.11 | 0.07 | 0 |
| CATIE R91 | Pound 7 x UF 273 | 0 | 0.11 | 0.31 | 0.13 | 0 | 0.19 | 0 | 0 | 0.19 | 0.06 |
| CATIE R92 | Pound 7 x UF 273 | 0 | 0.34 | 0.22 | 0 | 0 | 0.21 | 0 | 0 | 0.2 | 0.03 |
| CATIE R93 | Pound 7 x UF 273 | 0 | 0.28 | 0.28 | 0.04 | 0 | 0.26 | 0 | 0 | 0.04 | 0.1 |
| CATIE R94 | Pound 7 x UF 273 | 0 | 0.18 | 0.33 | 0.12 | 0 | 0.06 | 0 | 0 | 0.23 | 0.07 |
| CATIE R95 | Pound 7 x UF 273 | 0 | 0.47 | 0.19 | 0 | 0 | 0.23 | 0 | 0 | 0.1 | 0.01 |
| CATIE R96 | Pound 7 x UF 273 | 0 | 0.19 | 0.33 | 0.03 | 0 | 0.15 | 0.04 | 0.03 | 0.2 | 0.03 |
| CATIE R97 | Pound 7 x UF 273 | 0 | 0.18 | 0.31 | 0.1 | 0 | 0.11 | 0 | 0 | 0.26 | 0.03 |
| CATIE R98 | Pound 7 x UF 273 | 0 | 0.29 | 0.12 | 0.01 | 0.04 | 0.41 | 0 | 0.08 | 0.06 | 0 |
| CATIE R99 | Pound 7 x UF 273 | 0 | 0.4 | 0.19 | 0 | 0 | 0.32 | 0 | 0 | 0.07 | 0.02 |
| Caucasia 34 | Caucasia 34 | 0.44 | 0 | 0.15 | 0 | 0 | 0 | 0.02 | 0 | 0.39 | 0 |
| Caucasia 37 | Caucasia 37 | 0.43 | 0.05 | 0 | 0 | 0 | 0 | 0 | 0 | 0.52 | 0 |
| Caucasia 39 | Caucasia 39 | 0.48 | 0 | 0.06 | 0 | 0 | 0 | 0.04 | 0 | 0.42 | 0 |
| Caucasia 43 | Caucasia 43 | 0.44 | 0.02 | 0 | 0 | 0 | 0 | 0.02 | 0 | 0.52 | 0 |
| Caucasia 47 | Caucasia 47 | 0.44 | 0 | 0.15 | 0 | 0 | 0 | 0.02 | 0 | 0.39 | 0 |
| CC 137 | CC 137 | 0 | 0 | 0.65 | 0.35 | 0 | 0 | 0 | 0 | 0 | 0 |
| EET 605 | EET 605 | 0.13 | 0.39 | 0.02 | 0 | 0.45 | 0 | 0 | 0 | 0 | 0 |
| EET 610 | EET 610 | 0 | 0.27 | 0.11 | 0 | 0.47 | 0 | 0 | 0.08 | 0.07 | 0 |
| GU 128 N | GU 128 N | 0 | 0 | 0 | 0 | 0 | 0 | 1 | 0 | 0 | 0 |
| HY 2714184 | HY 2714184 | 0.46 | 0 | 0 | 0 | 0.04 | 0 | 0.02 | 0 | 0.49 | 0 |
| ICS 43red | ICS 43red | 0.09 | 0 | 0.4 | 0.52 | 0 | 0 | 0 | 0 | 0 | 0 |
| ICS 95 T1 | ICS 95 T1 | 0.08 | 0 | 0.4 | 0.52 | 0 | 0 | 0 | 0 | 0 | 0 |
| NAL 1 A13 | NAL 1 A13 | 0 | 0.55 | 0.41 | 0 | 0 | 0 | 0 | 0.04 | 0 | 0 |
| NAL 1 A14 | NAL 1 A14 | 0 | 0.61 | 0.39 | 0 | 0 | 0 | 0 | 0 | 0 | 0 |
| NAL 2 A26 | NAL 2 A26 | 0 | 0.27 | 0.25 | 0 | 0.05 | 0 | 0 | 0.02 | 0.4 | 0 |
| NAL 2 A27 | NAL 2 A27 | 0 | 0.28 | 0.72 | 0 | 0 | 0 | 0 | 0 | 0 | 0 |
| PA 107 | PA 107 | 0.86 | 0.02 | 0 | 0 | 0.12 | 0 | 0 | 0 | 0 | 0 |
| PA 120 | PA 120 | 1 | 0 | 0 | 0 | 0 | 0 | 0 | 0 | 0 | 0 |
| PA 169 | PA 169 | 1 | 0 | 0 | 0 | 0 | 0 | 0 | 0 | 0 | 0 |
| PMCT 46 | PMCT 46 | 0 | 0 | 0.71 | 0.25 | 0 | 0.03 | 0.01 | 0 | 0 | 0 |
| PMCT 58 | PMCT 58 | 0 | 0.15 | 0.47 | 0.06 | 0.1 | 0.06 | 0 | 0.03 | 0.14 | 0 |
| Porcelana 3 | Porcelana 3 | 0 | 0.13 | 0.71 | 0.15 | 0.01 | 0 | 0 | 0 | 0 | 0 |
| SC 24 | SC 24 | 0.5 | 0 | 0.24 | 0 | 0 | 0.07 | 0.07 | 0.05 | 0.05 | 0.02 |
| SNK 12 | SNK 12 | 0 | 0 | 0.81 | 0.19 | 0 | 0 | 0 | 0 | 0 | 0 |
| UF 12 | UF 12 | 0 | 0 | 0.52 | 0.48 | 0 | 0 | 0 | 0 | 0 | 0 |
| UF 273 Type 1 | UF 273 Type 1 | 0 | 0.46 | 0.3 | 0.09 | 0 | 0 | 0 | 0.12 | 0 | 0.02 |
| UF 273 Type 2 | UF 273 Type 2 | 0.00 | 0.59 | 0.28 | 0.05 | 0.02 | 0.00 | 0.00 | 0.06 | 0.00 | 0.01 |
| UF 712 | UF 712 | 0 | 1 | 0 | 0 | 0 | 0 | 0 | 0 | 0 | 0 |
| Yuca | Yuca | 0 | 0 | 0 | 1 | 0 | 0 | 0 | 0 | 0 | 0 |

**Supplementary Table 3**. Fitted yearly phenotypic values (BLUPs) for all evaluated clones.

| **Clone** | **Yield**  **(kg/ha*year)** | **Healthy**  **Pods** | **Infected**  **Frosty Pod** | **Proportion**  **Infected**  **Frosty Pod** | **Infected**  **Black Pod** | **Proportion**  **Infected**  **Black Pod** | **Pod**  **Index** |
| --- | --- | --- | --- | --- | --- | --- | --- |
| CATIE R92 | 1395.93 | 26.38 | 1.42 | 0.06 | 1.04 | 0.04 | 21 |
| CATIE R104 | 945.12 | 17.01 | 1.86 | 0.12 | 0.14 | 0.01 | 20 |
| CATIE R78 | 905.70 | 22.01 | 2.47 | 0.11 | 0.07 | 0.00 | 27 |
| CATIE R58 | 881.16 | 15.07 | 0.31 | 0.03 | 0.15 | 0.03 | 19 |
| CATIE R52 | 880.06 | 15.84 | 2.60 | 0.16 | 0.30 | 0.03 | 20 |
| CATIE R91 | 828.96 | 15.67 | 0.70 | 0.06 | 0.05 | 0.01 | 21 |
| CATIE R73 | 818.25 | 22.83 | 2.60 | 0.09 | 0.01 | 0.00 | 31 |
| CATIE R6 | 815.24 | 18.34 | 0.12 | 0.01 | 0.01 | 0.00 | 25 |
| CATIE R41 | 808.18 | 10.91 | 2.04 | 0.17 | 0.21 | 0.03 | 15 |
| CATIE R95 | 808.05 | 16.73 | 5.30 | 0.22 | 0.47 | 0.04 | 23 |
| CATIE R13 | 805.22 | 13.77 | 1.75 | 0.13 | 0.07 | 0.01 | 19 |
| CATIE R5 | 789.89 | 12.80 | 0.21 | 0.02 | 0.04 | 0.00 | 18 |
| CATIE R77 | 783.27 | 16.21 | 1.49 | 0.10 | 0.04 | 0.00 | 23 |
| CATIE R4 | 766.28 | 17.93 | 0.70 | 0.05 | 0.14 | 0.02 | 26 |
| CATIE R49 | 753.37 | 15.59 | 3.36 | 0.19 | 0.33 | 0.04 | 23 |
| CATIE R133 | 743.02 | 16.05 | 0.44 | 0.05 | 0.07 | 0.01 | 24 |
| CATIE R121 | 698.31 | 19.48 | 0.44 | 0.04 | 0.03 | 0.00 | 31 |
| CATIE R75 | 692.96 | 21.83 | 0.74 | 0.05 | 0.02 | 0.00 | 35 |
| CATIE R30 | 691.92 | 15.57 | 2.00 | 0.10 | 0.19 | 0.02 | 25 |
| CATIE R89 | 689.84 | 14.90 | 0.33 | 0.04 | 0.06 | 0.01 | 24 |
| CATIE R37 | 683.90 | 12.93 | 0.57 | 0.06 | 0.09 | 0.01 | 21 |
| CATIE R76 | 666.64 | 16.20 | 5.33 | 0.22 | 0.16 | 0.01 | 27 |
| CATIE R71 | 657.75 | 14.21 | 6.58 | 0.31 | 0.04 | 0.01 | 24 |
| CATIE R98 | 657.51 | 10.65 | 2.68 | 0.21 | 0.09 | 0.01 | 18 |
| CATIE R20 | 646.86 | 11.64 | 2.48 | 0.18 | 0.14 | 0.02 | 20 |
| Caucasia 47 | 646.82 | 13.39 | 0.75 | 0.07 | 0.21 | 0.02 | 23 |
| CATIE R7 | 643.75 | 14.48 | 1.18 | 0.10 | 0.46 | 0.03 | 25 |
| CATIE R101 | 643.05 | 7.52 | 1.30 | 0.17 | 0.02 | 0.00 | 13 |
| CATIE R18 | 636.24 | 12.60 | 1.00 | 0.06 | 0.49 | 0.04 | 22 |
| CATIE R35 | 633.29 | 13.68 | 1.59 | 0.10 | 0.39 | 0.05 | 24 |
| CATIE R28 | 631.90 | 10.24 | 3.23 | 0.23 | 0.36 | 0.04 | 18 |
| CATIE R19 | 620.30 | 10.61 | 1.11 | 0.12 | 0.11 | 0.01 | 19 |
| CATIE R65 | 616.28 | 11.09 | 4.91 | 0.29 | 0.07 | 0.01 | 20 |
| CATIE R22 | 610.93 | 9.90 | 0.99 | 0.09 | 0.21 | 0.02 | 18 |
| CATIE R130 | 591.49 | 16.50 | 0.82 | 0.06 | 0.13 | 0.01 | 31 |
| CATIE R93 | 580.78 | 12.54 | 5.48 | 0.27 | 0.54 | 0.05 | 24 |
| CATIE R70 | 579.89 | 15.66 | 0.95 | 0.07 | 0.07 | 0.01 | 30 |
| CATIE R90 | 579.33 | 13.03 | 1.07 | 0.10 | 0.41 | 0.04 | 25 |
| CATIE R34 | 575.91 | 9.33 | 0.71 | 0.10 | 0.26 | 0.05 | 18 |
| CATIE R42 | 573.18 | 8.25 | 12.52 | 0.53 | 0.03 | 0.00 | 16 |
| CATIE R40 | 567.77 | 5.62 | 1.07 | 0.19 | 0.23 | 0.05 | 11 |
| CATIE R15 | 559.05 | 12.08 | 1.60 | 0.13 | 0.11 | 0.02 | 24 |
| CATIE R38 | 555.28 | 7.50 | 0.84 | 0.08 | 0.07 | 0.01 | 15 |
| Caucasia 34 | 542.17 | 14.64 | 0.86 | 0.06 | 0.17 | 0.02 | 30 |
| CATIE R24 | 539.02 | 13.10 | 1.12 | 0.11 | 0.31 | 0.04 | 27 |
| CATIE R79 | 537.88 | 15.01 | 1.25 | 0.11 | 0.04 | 0.01 | 31 |
| CATIE R56 | 537.59 | 18.87 | 1.92 | 0.10 | 0.53 | 0.04 | 39 |
| CATIE R12 | 536.08 | 11.58 | 1.13 | 0.09 | 0.09 | 0.01 | 24 |
| CATIE R102 | 534.88 | 10.59 | 0.87 | 0.09 | 0.11 | 0.02 | 22 |
| CATIE R114 | 517.17 | 16.76 | 1.03 | 0.07 | 0.20 | 0.01 | 36 |
| CATIE R128 | 513.32 | 12.47 | 0.41 | 0.05 | 0.02 | 0.00 | 27 |
| CATIE R32 | 507.66 | 9.59 | 0.79 | 0.07 | 0.40 | 0.05 | 21 |
| CATIE R23 | 507.42 | 10.96 | 2.30 | 0.15 | 0.58 | 0.03 | 24 |
| CATIE R109 | 502.28 | 14.47 | 0.98 | 0.09 | 0.34 | 0.03 | 32 |
| CATIE R11 | 500.21 | 17.56 | 1.96 | 0.12 | 0.04 | 0.01 | 39 |
| CATIE R17 | 495.75 | 17.40 | 1.26 | 0.07 | 0.44 | 0.04 | 39 |
| CATIE R47 | 494.55 | 12.02 | 2.04 | 0.17 | 0.25 | 0.03 | 27 |
| CATIE R27 | 486.44 | 13.13 | 1.40 | 0.11 | 0.42 | 0.04 | 30 |
| PA 169 | 480.56 | 8.22 | 0.27 | 0.03 | 0.01 | 0.00 | 19 |
| CATIE R99 | 477.19 | 11.60 | 3.81 | 0.24 | 1.32 | 0.10 | 27 |
| CATIE R125 | 474.62 | 11.96 | 0.77 | 0.09 | 0.04 | 0.01 | 28 |
| CATIE R124 | 472.43 | 18.28 | 0.33 | 0.03 | 0.05 | 0.01 | 43 |
| CATIE R117 | 468.45 | 8.43 | 1.27 | 0.16 | 0.05 | 0.01 | 20 |
| CATIE R97 | 464.39 | 11.28 | 2.58 | 0.17 | 0.10 | 0.01 | 27 |
| CATIE R9 | 461.37 | 9.97 | 1.23 | 0.12 | 0.07 | 0.01 | 24 |
| CATIE R138 | 452.23 | 8.95 | 1.24 | 0.18 | 0.05 | 0.01 | 22 |
| CATIE R112 | 447.49 | 10.87 | 0.55 | 0.08 | 0.11 | 0.01 | 27 |
| CATIE R122 | 442.56 | 13.14 | 1.22 | 0.09 | 0.19 | 0.02 | 33 |
| CATIE R36 | 423.53 | 11.05 | 0.54 | 0.08 | 0.18 | 0.03 | 29 |
| CATIE R94 | 417.48 | 8.27 | 1.06 | 0.14 | 0.05 | 0.01 | 22 |
| CATIE R137 | 414.86 | 10.83 | 0.38 | 0.05 | 0.06 | 0.01 | 29 |
| CATIE R111 | 411.88 | 9.27 | 0.47 | 0.06 | 0.32 | 0.08 | 25 |
| CATIE R21 | 411.23 | 7.77 | 1.86 | 0.16 | 0.11 | 0.02 | 21 |
| CATIE R135 | 410.24 | 9.97 | 0.88 | 0.11 | 0.02 | 0.00 | 27 |
| Caucasia 37 | 407.70 | 14.68 | 1.68 | 0.11 | 0.09 | 0.01 | 40 |
| CATIE R50 | 404.60 | 12.74 | 2.95 | 0.16 | 0.29 | 0.03 | 35 |
| CATIE R64 | 402.69 | 17.40 | 1.84 | 0.11 | 0.04 | 0.00 | 48 |
| CATIE R57 | 397.31 | 15.73 | 2.49 | 0.13 | 0.21 | 0.02 | 44 |
| CATIE R62 | 393.43 | 9.56 | 0.88 | 0.10 | 0.20 | 0.02 | 27 |
| Caucasia 39 | 373.81 | 12.11 | 0.71 | 0.10 | 0.11 | 0.02 | 36 |
| GU 128 N | 364.52 | 9.51 | 3.25 | 0.24 | 0.12 | 0.02 | 29 |
| CATIE R131 | 363.53 | 14.07 | 0.32 | 0.04 | 0.03 | 0.01 | 43 |
| CC 137 | 358.82 | 9.37 | 1.62 | 0.17 | 0.06 | 0.01 | 29 |
| CATIE R110 | 356.26 | 14.43 | 1.03 | 0.10 | 0.22 | 0.02 | 45 |
| CATIE R14 | 350.81 | 11.37 | 1.00 | 0.11 | 0.05 | 0.01 | 36 |
| CATIE R96 | 346.71 | 9.99 | 1.24 | 0.14 | 0.03 | 0.01 | 32 |
| CATIE R44 | 327.03 | 10.60 | 2.20 | 0.20 | 0.15 | 0.01 | 36 |
| Caucasia 43 | 326.69 | 14.41 | 1.97 | 0.14 | 0.11 | 0.01 | 49 |
| CATIE R60 | 325.97 | 9.97 | 1.19 | 0.14 | 0.16 | 0.04 | 34 |
| CATIE R33 | 325.37 | 8.49 | 0.86 | 0.12 | 0.17 | 0.04 | 29 |
| CATIE R82 | 320.80 | 9.82 | 1.34 | 0.10 | 0.23 | 0.04 | 34 |
| CATIE R108 | 314.97 | 7.94 | 0.69 | 0.09 | 0.08 | 0.02 | 28 |
| CATIE R116 | 313.47 | 13.54 | 0.72 | 0.06 | 0.06 | 0.01 | 48 |
| CATIE R3 | 307.90 | 9.42 | 0.62 | 0.10 | 0.08 | 0.02 | 34 |
| CATIE R120 | 305.91 | 8.26 | 1.56 | 0.19 | 0.07 | 0.01 | 30 |
| CATIE R29 | 305.21 | 5.49 | 0.82 | 0.16 | 0.13 | 0.04 | 20 |
| CATIE R85 | 291.20 | 6.81 | 0.38 | 0.07 | 0.12 | 0.02 | 26 |
| UF 273 Type 1 | 291.07 | 9.69 | 0.64 | 0.10 | 0.17 | 0.03 | 37 |
| CATIE R72 | 282.15 | 7.87 | 0.79 | 0.12 | 0.01 | 0.00 | 31 |
| CATIE R25 | 281.57 | 6.34 | 0.33 | 0.08 | 0.11 | 0.04 | 25 |
| PA 107 | 279.72 | 5.79 | 1.66 | 0.21 | 0.05 | 0.01 | 23 |
| CATIE R26 | 278.92 | 5.27 | 0.41 | 0.14 | 0.02 | 0.01 | 21 |
| NAL 2 A26 | 276.43 | 5.97 | 0.98 | 0.19 | 0.13 | 0.03 | 24 |
| CATIE R54 | 274.07 | 8.39 | 9.60 | 0.42 | 0.27 | 0.03 | 34 |
| CATIE R105 | 271.69 | 7.34 | 0.53 | 0.07 | 0.03 | 0.01 | 30 |
| CATIE R10 | 269.89 | 8.26 | 0.81 | 0.09 | 0.26 | 0.03 | 34 |
| CATIE R61 | 261.25 | 4.94 | 0.34 | 0.08 | 0.01 | 0.00 | 21 |
| NAL 2 A27 | 256.51 | 12.47 | 2.36 | 0.17 | 0.02 | 0.00 | 54 |
| CATIE R68 | 256.26 | 4.84 | 1.00 | 0.20 | 0.02 | 0.01 | 21 |
| CATIE R134 | 254.75 | 6.65 | 0.27 | 0.06 | 0.01 | 0.00 | 29 |
| CATIE R43 | 250.70 | 5.64 | 3.75 | 0.36 | 0.11 | 0.02 | 25 |
| CATIE R39 | 244.14 | 4.61 | 0.60 | 0.14 | 0.04 | 0.01 | 21 |
| NAL 1 A13 | 233.48 | 4.83 | 0.35 | 0.09 | 0.12 | 0.06 | 23 |
| CATIE R103 | 232.61 | 14.03 | 1.12 | 0.09 | 0.27 | 0.03 | 67 |
| CATIE R113 | 231.95 | 15.66 | 1.55 | 0.11 | 0.19 | 0.02 | 75 |
| CATIE R118 | 229.97 | 4.76 | 0.49 | 0.17 | 0.03 | 0.01 | 23 |
| CAP 34 | 229.97 | 4.97 | 2.12 | 0.27 | 0.30 | 0.04 | 24 |
| PMCT 58 | 224.03 | 11.69 | 1.64 | 0.13 | 0.26 | 0.02 | 58 |
| CATIE R136 | 219.35 | 9.08 | 0.39 | 0.06 | 0.08 | 0.02 | 46 |
| CATIE R66 | 216.44 | 7.40 | 0.84 | 0.14 | 0.04 | 0.01 | 38 |
| CATIE R119 | 197.00 | 6.74 | 0.37 | 0.07 | 0.01 | 0.00 | 38 |
| CATIE R8 | 196.87 | 6.02 | 0.60 | 0.09 | 0.07 | 0.01 | 34 |
| ICS 95 T1 | 187.73 | 3.72 | 1.11 | 0.26 | 0.20 | 0.05 | 22 |
| EET 605 | 185.78 | 4.35 | 0.63 | 0.15 | 0.08 | 0.03 | 26 |
| CATIE R106 | 181.54 | 7.52 | 0.92 | 0.11 | 0.06 | 0.01 | 46 |
| ICS 43red | 176.50 | 5.88 | 1.57 | 0.23 | 0.24 | 0.03 | 37 |
| CATIE R63 | 172.91 | 7.78 | 1.11 | 0.15 | 0.14 | 0.02 | 50 |
| SC 24 | 165.44 | 3.57 | 0.76 | 0.17 | 0.01 | 0.00 | 24 |
| CATIE R48 | 163.17 | 3.67 | 0.75 | 0.17 | 0.02 | 0.01 | 25 |
| CATIE R107 | 144.87 | 6.91 | 2.42 | 0.22 | 0.16 | 0.02 | 53 |
| UF 712 | 128.97 | 2.44 | 0.16 | 0.03 | 0.07 | 0.02 | 21 |
| CATIE R86 | 128.78 | 2.67 | 0.39 | 0.19 | 0.04 | 0.02 | 23 |
| CATIE R87 | 128.04 | 4.38 | 0.60 | 0.13 | 0.05 | 0.01 | 38 |
| UF 12 | 120.78 | 4.78 | 2.72 | 0.33 | 0.15 | 0.02 | 44 |
| EET 610 | 115.10 | 3.31 | 0.18 | 0.06 | 0.01 | 0.00 | 32 |
| CATIE R80 | 114.22 | 3.39 | 0.28 | 0.09 | 0.01 | 0.00 | 33 |
| CATIE R31 | 107.26 | 3.76 | 0.21 | 0.09 | 0.02 | 0.01 | 39 |
| HY 2714184 | 104.52 | 5.27 | 0.51 | 0.10 | 0.06 | 0.02 | 56 |
| SNK 12 | 74.42 | 2.21 | 0.86 | 0.25 | 0.05 | 0.01 | 33 |
| Porcelana 3 | 70.70 | 2.10 | 1.38 | 0.47 | 0.02 | 0.01 | 33 |
| PMCT 46 | 66.98 | 1.39 | 0.33 | 0.19 | 0.01 | 0.01 | 23 |
| CATIE R132 | 63.93 | 2.24 | 0.34 | 0.18 | 0.01 | 0.01 | 39 |
| CATIE R126 | 51.22 | 1.11 | 0.05 | 0.05 | 0.00 | 0.00 | 24 |
| NAL 1 A14 | 51.21 | 1.24 | 0.26 | 0.16 | 0.05 | 0.02 | 27 |
| PA 120 | 28.44 | 0.56 | 0.14 | 0.12 | 0.00 | 0.00 | 22 |
| CATIE R129 | 27.24 | 0.59 | 0.03 | 0.01 | 0.00 | 0.00 | 24 |
| CATIE R127 | 16.03 | 0.45 | 0.03 | 0.03 | 0.01 | 0.01 | 31 |
| Yuca | 11.30 | 0.24 | 0.06 | 0.16 | 0.00 | 0.00 | 24 |

**Supplementary table 4.** Analysis of variance. The model fits the number of pods per tree, the effect of position in the block, and the effect of the rootstock. For the rootstock, the estimates are in reference to SPA 9. All rootstocks had a significant effect on pod number, with PA 121 having the largest negative effect and lowest p-value

Model: Total Pods ~ block + rootstock + ε

Analysis of Variance Table

|  | Df | Sum Sq | Mean Sq | F value | Pr(>F) |
| --- | --- | --- | --- | --- | --- |
| Block | 2 | 636 | 317.93 | 51.034 | < 2.2e-16 *** |
| Rootstock | 4 | 2431 | 607.69 | 97.546 | < 2.2e-16 *** |
| Residuals | 215909 | 1345076 | 6.23 |  |  |

|  | Estimate | Std. Error | t value | Pr(>|t|) |
| --- | --- | --- | --- | --- |
| (Intercept) | 1.12752 | 0.01273 | 88.538 | < 2e-16 *** |
| Block2 | 0.15953 | 0.01759 | 9.070 | < 2e-16 *** |
| Block3 | 0.11025 | 0.01236 | 8.923 | < 2e-16 *** |
| Rootstock_IMC 67 | -0.08672 | 0.01350 | -6.423 | 1.34e-10 *** |
| Rootstock_EET 400 | 0.05815 | 0.01689 | 3.443 | 0.000576 *** |
| Rootstock_UF 613 | 0.04203 | 0.01844 | 2.279 | 0.022684 * |
| Rootstock_PA 121 | -0.31862 | 0.01931 | -16.498 | < 2e-16 *** |

**Supplementary table 5.** Differential expressed genes annotated as protein coding, identified using the Bioconductor Limma package [(Ritchie et al. 2015)](https://paperpile.com/c/Q460iz/4syrb). eBayes adjusted P-values were corrected for multiple testing using the Benjamin-Hochberg (BH) method and used to select genes with significant expression differences (q < 0.01).

| **GeneID** | **Symbol** | **logFC** | **AveExpr** | **t** | **adj.P.Val** | **B** |
| --- | --- | --- | --- | --- | --- | --- |
| Thecc1EG014463 | Uncharacterized protein | 3.70 | 8.90 | 40.86 | 8.37E-04 | 8.05 |
| Thecc1EG042590 | S-adenosyl-L-methionine-dependent methyltransferases superfamily protein | 3.17 | 6.17 | 37.96 | 8.37E-04 | 7.71 |
| Thecc1EG033952 | Cytochrome P450 | 4.28 | 3.39 | 33.66 | 8.37E-04 | 7.04 |
| Thecc1EG030052 | Uncharacterized protein | -5.16 | 3.18 | -33.35 | 8.37E-04 | 6.84 |
| Thecc1EG046209 | 3R-linalool synthase,putative | -4.88 | 3.39 | -30.85 | 8.37E-04 | 6.61 |
| Thecc1EG012538 | Ankyrin repeat family protein,putative | -2.86 | 3.95 | -29.21 | 8.37E-04 | 6.47 |
| Thecc1EG040641 | Prephenate dehydrogenase,putative | 2.23 | 5.36 | 29.16 | 8.37E-04 | 6.41 |
| Thecc1EG044758 | Sulfotransferase 2A,putative | 2.18 | 5.02 | 28.26 | 8.37E-04 | 6.26 |
| Thecc1EG020810 | Cellulose synthase like G2,putative | 7.91 | 2.56 | 32.41 | 8.37E-04 | 6.25 |
| Thecc1EG032794 | Cytochrome P450 | 4.86 | 2.91 | 28.16 | 8.37E-04 | 6.18 |
| Thecc1EG001215 | Gamma-glutamyl transpeptidase 1 | -5.23 | 1.81 | -29.93 | 8.37E-04 | 6.14 |
| Thecc1EG005418 | Cytochrome P450 87A3 | 2.40 | 8.23 | 27.64 | 8.37E-04 | 6.12 |
| Thecc1EG025554 | GroES-like zinc-binding dehydrogenase family protein | -2.21 | 7.83 | -27.61 | 8.37E-04 | 6.11 |
| Thecc1EG016005 | Nitrate transporter 1.1 | -5.08 | 3.28 | -27.05 | 8.37E-04 | 6.04 |
| Thecc1EG010342 | Uncharacterized protein | -7.49 | 2.23 | -30.44 | 8.37E-04 | 5.99 |
| Thecc1EG042221 | Phosphatase 2C family protein | 3.85 | 2.61 | 26.20 | 8.37E-04 | 5.92 |
| Thecc1EG006195 | Serine/threonine-protein kinase PBS1 | -5.48 | 1.69 | -28.66 | 8.37E-04 | 5.92 |
| Thecc1EG040754 | ABC-2 type transporter family protein | -3.08 | 5.80 | -26.36 | 8.37E-04 | 5.89 |
| Thecc1EG009468 | Indole-3-acetic acid inducible 34,putative | 9.32 | -1.33 | 36.99 | 8.37E-04 | 5.88 |
| Thecc1EG042400 | NAD(P)-binding Rossmann-fold superfamily protein | -5.19 | 3.03 | -26.06 | 8.37E-04 | 5.86 |
| Thecc1EG004338 | Transposase tnp2 | -9.55 | -1.08 | -35.60 | 8.37E-04 | 5.84 |
| Thecc1EG029210 | Uncharacterized protein | -9.04 | -1.34 | -35.07 | 8.37E-04 | 5.77 |
| Thecc1EG024923 | Ribosomal protein L6 family protein | -2.56 | 4.66 | -25.49 | 8.37E-04 | 5.77 |
| Thecc1EG026484 | NB-ARC domain-containing disease resistance protein,putative | -9.13 | -1.29 | -35.02 | 8.37E-04 | 5.77 |
| Thecc1EG004718 | Uncharacterized protein | -8.91 | -1.40 | -34.78 | 8.37E-04 | 5.75 |
| Thecc1EG042905 | Ankyrin repeat-containing protein,putative | -3.68 | 2.58 | -25.31 | 8.37E-04 | 5.72 |
| Thecc1EG020935 | Alcohol oxidase,putative | -1.68 | 6.40 | -25.71 | 8.37E-04 | 5.70 |
| Thecc1EG019362 | Uncharacterized protein | 4.80 | 2.21 | 25.51 | 8.37E-04 | 5.70 |
| Thecc1EG042906 | Ankyrin repeat-containing protein | -4.58 | 1.84 | -26.00 | 8.37E-04 | 5.67 |
| Thecc1EG000949 | Geraniol dehydrogenase 1 | 2.23 | 3.91 | 24.97 | 8.44E-04 | 5.61 |
| Thecc1EG019813 | Hydroxycinnamoyl CoA shikimate/quinate hydroxycinnamoyltransferase | 9.73 | 2.14 | 30.03 | 8.37E-04 | 5.59 |
| Thecc1EG005673 | Uncharacterized protein | 3.12 | 4.84 | 24.44 | 8.78E-04 | 5.56 |
| Thecc1EG031009 | Uncharacterized protein | 8.99 | -1.49 | 31.69 | 8.37E-04 | 5.56 |
| Thecc1EG032861 | NAD(P)-binding Rossmann-fold superfamily protein | -4.98 | 1.36 | -26.07 | 8.37E-04 | 5.56 |
| Thecc1EG005676 | Uncharacterized protein | 7.16 | 2.42 | 25.44 | 8.37E-04 | 5.55 |
| Thecc1EG014020 | Uncharacterized protein | 8.83 | -1.57 | 31.09 | 8.37E-04 | 5.52 |
| Thecc1EG006188 | Serine/threonine-protein kinase PBS1 | -3.87 | 4.28 | -23.94 | 9.10E-04 | 5.51 |
| Thecc1EG006194 | Embryogenesis-associated protein | -8.49 | -1.62 | -31.21 | 8.37E-04 | 5.49 |
| Thecc1EG021779 | Neutral/alkaline non-lysosomal ceramidase | -3.64 | 3.49 | -23.78 | 9.10E-04 | 5.49 |
| Thecc1EG038101 | Equilibrative nucleoside transporter | -4.44 | 2.18 | -24.17 | 8.98E-04 | 5.49 |
| Thecc1EG028623 | Galactose oxidase/kelch repeat superfamily protein,putative | 3.13 | 3.42 | 23.75 | 9.10E-04 | 5.48 |
| Thecc1EG034534 | Sas10/Utp3/C1D family | -1.67 | 6.19 | -24.67 | 8.72E-04 | 5.48 |
| Thecc1EG004336 | Uncharacterized protein | -8.35 | -1.68 | -30.80 | 8.37E-04 | 5.45 |
| Thecc1EG015788 | Bifunctional inhibitor/lipid-transfer protein/seed storage 2S albumin superfamily protein | 3.28 | 4.29 | 23.54 | 9.36E-04 | 5.40 |
| Thecc1EG018917 | Uncharacterized protein | 7.92 | -2.03 | 30.69 | 8.37E-04 | 5.39 |
| Thecc1EG004119 | Poly(ADP-ribose) polymerase 2,putative | -2.69 | 3.60 | -23.31 | 9.44E-04 | 5.39 |
| Thecc1EG042042 | RING/U-box superfamily protein | -3.21 | 4.07 | -23.20 | 9.44E-04 | 5.36 |
| Thecc1EG002064 | Pentatricopeptide repeat-containing,putative-like protein | 9.02 | -1.48 | 28.68 | 8.37E-04 | 5.35 |
| Thecc1EG010343 | Uncharacterized protein | -8.41 | -1.66 | -28.97 | 8.37E-04 | 5.32 |
| Thecc1EG001777 | Unknown | -7.93 | -1.89 | -29.61 | 8.37E-04 | 5.32 |
| Thecc1EG025177 | UDP-Glycosyltransferase superfamily protein,putative | -2.18 | 5.48 | -23.75 | 9.10E-04 | 5.32 |
| Thecc1EG019335 | Cc-nbs-lrr resistance protein,putative | -5.49 | 1.66 | -23.98 | 9.10E-04 | 5.28 |
| Thecc1EG026478 | NB-ARC domain-containing disease resistance protein,putative | -11.99 | 0.67 | -27.44 | 8.37E-04 | 5.28 |
| Thecc1EG013932 | Uncharacterized protein | 3.86 | 4.68 | 22.97 | 9.61E-04 | 5.28 |
| Thecc1EG026479 | Uncharacterized protein | -7.78 | -1.97 | -29.24 | 8.37E-04 | 5.26 |
| Thecc1EG042106 | Uncharacterized protein | 7.70 | -1.61 | 28.79 | 8.37E-04 | 5.24 |
| Thecc1EG029278 | RING/U-box superfamily protein | 9.18 | 1.55 | 26.58 | 8.37E-04 | 5.22 |
| Thecc1EG022248 | AMP-dependent synthetase and ligase family protein,putative | 6.69 | -0.04 | 26.84 | 8.37E-04 | 5.20 |
| Thecc1EG027222 | TMV resistance protein N | -6.47 | 1.97 | -23.47 | 9.36E-04 | 5.20 |
| Thecc1EG000969 | Uncharacterized protein | -7.70 | -2.01 | -28.40 | 8.37E-04 | 5.19 |
| Thecc1EG014825 | Leucine-rich repeat protein kinase family protein | -8.66 | -1.53 | -26.91 | 8.37E-04 | 5.18 |
| Thecc1EG020637 | Uncharacterized protein | -3.40 | 1.73 | -22.27 | 9.93E-04 | 5.16 |
| Thecc1EG022566 | Uncharacterized protein | 7.66 | -2.16 | 27.63 | 8.37E-04 | 5.14 |
| Thecc1EG002057 | Polyubiquitin 10 | -12.37 | 1.38 | -25.91 | 8.37E-04 | 5.13 |
| Thecc1EG001183 | FK506-binding protein 16-2 | 4.78 | 1.48 | 22.82 | 9.61E-04 | 5.12 |
| Thecc1EG029610 | Leucine-rich repeat transmembrane protein kinase,putative | 7.62 | -2.18 | 27.72 | 8.37E-04 | 5.12 |
| Thecc1EG010995 | Plant cadmium resistance 2 | 2.37 | 5.07 | 22.57 | 9.83E-04 | 5.09 |
| Thecc1EG037710 | S-adenosyl-L-methionine-dependent methyltransferases superfamily protein | 7.36 | 0.77 | 24.98 | 8.44E-04 | 5.06 |
| Thecc1EG032160 | Sulfotransferase 2A | -10.88 | 1.66 | -25.19 | 8.37E-04 | 5.05 |
| Thecc1EG037264 | Serine hydroxymethyltransferase 3 | 2.28 | 4.17 | 22.01 | 1.01E-03 | 5.04 |
| Thecc1EG009987 | Nitrate transporter 1.7,putative | -7.71 | -1.48 | -25.64 | 8.37E-04 | 5.04 |
| Thecc1EG011008 | Unknown | 7.95 | -2.02 | 26.06 | 8.37E-04 | 5.03 |
| Thecc1EG041996 | Cysteine-rich RLK (RECEPTOR-like protein kinase) 8 | -7.86 | -1.93 | -26.20 | 8.37E-04 | 5.01 |
| Thecc1EG002211 | Uncharacterized protein | -7.52 | -2.10 | -26.74 | 8.37E-04 | 5.00 |
| Thecc1EG042587 | S-adenosyl-L-methionine-dependent methyltransferases superfamily protein | 2.60 | 7.31 | 22.39 | 9.86E-04 | 4.99 |
| Thecc1EG046327 | Ribonuclease 2,putative | -8.06 | -1.83 | -25.69 | 8.37E-04 | 4.97 |
| Thecc1EG029416 | Chaperone DnaJ-domain-containing protein,putative | -2.03 | 4.26 | -21.70 | 1.03E-03 | 4.97 |
| Thecc1EG006050 | Uncharacterized protein | -11.06 | 0.20 | -24.32 | 8.85E-04 | 4.95 |
| Thecc1EG043925 | Uncharacterized protein | -7.23 | -2.25 | -26.51 | 8.37E-04 | 4.95 |
| Thecc1EG025058 | Uncharacterized protein | 7.21 | -2.38 | 26.27 | 8.37E-04 | 4.94 |
| Thecc1EG020371 | Uncharacterized protein | -7.90 | -1.91 | -25.25 | 8.37E-04 | 4.94 |
| Thecc1EG001717 | Unknown | -6.27 | -0.24 | -24.48 | 8.78E-04 | 4.93 |
| Thecc1EG006906 | Uncharacterized protein | 2.51 | 5.89 | 22.00 | 1.01E-03 | 4.92 |
| Thecc1EG043585 | Unknown | -7.18 | -2.27 | -26.23 | 8.37E-04 | 4.92 |
| Thecc1EG032047 | Leucine-rich repeat transmembrane protein kinase,putative | -5.23 | 1.33 | -21.45 | 1.03E-03 | 4.91 |
| Thecc1EG004271 | Indeterminate(ID)-domain 12 | -2.66 | 3.04 | -20.62 | 1.17E-03 | 4.81 |
| Thecc1EG004881 | Galactinol synthase 1 | -3.21 | 3.30 | -20.65 | 1.17E-03 | 4.80 |
| Thecc1EG018865 | Uncharacterized protein | -8.24 | -0.68 | -23.17 | 9.44E-04 | 4.80 |
| Thecc1EG011289 | Stem 28 kDa glycoprotein,putative | 3.08 | 6.77 | 21.53 | 1.03E-03 | 4.79 |
| Thecc1EG041903 | Uncharacterized protein | 9.11 | -0.90 | 22.79 | 9.61E-04 | 4.75 |
| Thecc1EG020844 | 2-oxoglutarate (2OG) and Fe(II)-dependent oxygenase superfamily protein | 3.07 | 2.89 | 20.33 | 1.18E-03 | 4.74 |
| Thecc1EG031025 | Leucine-rich repeat containing protein,putative | 6.83 | -2.57 | 24.63 | 8.72E-04 | 4.73 |
| Thecc1EG040638 | Uncharacterized protein | 6.89 | -1.23 | 22.84 | 9.61E-04 | 4.70 |
| Thecc1EG020721 | Zinc finger,CCHC-type | 8.00 | -1.99 | 22.93 | 9.61E-04 | 4.69 |
| Thecc1EG044767 | Sulfotransferase 2A,putative | -2.61 | 4.68 | -20.67 | 1.17E-03 | 4.67 |
| Thecc1EG005901 | Uncharacterized protein | 3.48 | 4.34 | 20.36 | 1.18E-03 | 4.66 |
| Thecc1EG020723 | Flavonol 3-sulfotransferase,putative | 10.41 | 2.00 | 21.96 | 1.01E-03 | 4.65 |
| Thecc1EG025301 | Unknown | -5.13 | 1.45 | -20.24 | 1.18E-03 | 4.63 |
| Thecc1EG002692 | Unknown | -5.71 | -0.36 | -22.25 | 9.93E-04 | 4.60 |
| Thecc1EG045413 | Uncharacterized protein | 9.48 | -0.19 | 21.60 | 1.03E-03 | 4.60 |
| Thecc1EG020854 | 2-oxoglutarate (2OG) and Fe(II)-dependent oxygenase superfamily protein,putative | 7.30 | -1.56 | 22.42 | 9.86E-04 | 4.59 |
| Thecc1EG022379 | Exostosin family protein | -1.53 | 5.26 | -20.72 | 1.17E-03 | 4.58 |
| Thecc1EG012111 | Ankyrin repeat family protein,putative | 5.11 | 0.72 | 20.47 | 1.17E-03 | 4.56 |
| Thecc1EG029151 | Uncharacterized protein | -11.29 | 0.72 | -21.35 | 1.05E-03 | 4.56 |
| Thecc1EG023478 | Zinc-binding dehydrogenase family protein | -1.68 | 5.12 | -20.49 | 1.17E-03 | 4.55 |
| Thecc1EG031631 | Disease resistance protein family | -6.85 | -2.44 | -23.28 | 9.44E-04 | 4.55 |
| Thecc1EG027225 | Histidine kinase | -7.76 | -0.92 | -21.49 | 1.03E-03 | 4.54 |
| Thecc1EG042107 | Uncharacterized protein | 8.18 | -1.90 | 21.81 | 1.02E-03 | 4.53 |
| Thecc1EG018347 | Unknown | 6.87 | -2.56 | 22.63 | 9.82E-04 | 4.52 |
| Thecc1EG012328 | Uncharacterized protein | -2.15 | 3.07 | -19.55 | 1.27E-03 | 4.51 |
| Thecc1EG000968 | UDP-Glycosyltransferase superfamily protein,putative | -7.07 | -2.33 | -22.43 | 9.86E-04 | 4.49 |
| Thecc1EG029266 | Triacylglycerol lipase,putative | 4.02 | 1.98 | 19.13 | 1.36E-03 | 4.46 |
| Thecc1EG014887 | Biotin F | 6.19 | 0.24 | 20.65 | 1.17E-03 | 4.45 |
| Thecc1EG020166 | Wall associated kinase-like 6,putative | -6.63 | -2.01 | -22.20 | 9.93E-04 | 4.44 |
| Thecc1EG042146 | Alpha-L-fucosidase 1 | -1.53 | 4.83 | -19.90 | 1.22E-03 | 4.42 |
| Thecc1EG018892 | Uncharacterized protein | -10.78 | 0.06 | -20.40 | 1.17E-03 | 4.41 |
| Thecc1EG013885 | Calcineurin-like metallo-phosphoesterase superfamily protein | 1.44 | 5.15 | 20.03 | 1.20E-03 | 4.40 |
| Thecc1EG046330 | Serine-threonine protein kinase,plant-type,putative | -2.01 | 3.71 | -19.24 | 1.35E-03 | 4.40 |
| Thecc1EG027013 | Uncharacterized protein | 7.56 | -0.63 | 20.49 | 1.17E-03 | 4.40 |
| Thecc1EG008805 | Cysteine-rich RLK (RECEPTOR-like protein kinase) 8 | -6.78 | -2.47 | -21.78 | 1.02E-03 | 4.40 |
| Thecc1EG031726 | Uncharacterized protein | -2.24 | 3.75 | -19.24 | 1.35E-03 | 4.37 |
| Thecc1EG027015 | Cysteine/Histidine-rich C1 domain family protein | 6.78 | 0.32 | 20.06 | 1.20E-03 | 4.36 |
| Thecc1EG001757 | Unknown | -6.79 | -2.47 | -21.47 | 1.03E-03 | 4.35 |
| Thecc1EG010241 | Unknown | 6.48 | -2.75 | 21.79 | 1.02E-03 | 4.35 |
| Thecc1EG004163 | Inhibitor of trypsin and hageman factor | -7.45 | -0.82 | -20.18 | 1.19E-03 | 4.34 |
| Thecc1EG025134 | Pleiotropic drug resistance 3,putative | 10.11 | -0.15 | 19.98 | 1.20E-03 | 4.34 |
| Thecc1EG013857 | Aldehyde dehydrogenase 12A1 | -1.86 | 5.93 | -19.83 | 1.23E-03 | 4.32 |
| Thecc1EG018843 | Malectin/receptor protein kinase family protein | 4.42 | 0.49 | 19.16 | 1.36E-03 | 4.31 |
| Thecc1EG033810 | Subtilisin-like serine endopeptidase family protein | -2.21 | 2.88 | -18.66 | 1.42E-03 | 4.30 |
| Thecc1EG042216 | UDP-Glycosyltransferase superfamily protein,putative | 2.12 | 3.71 | 18.84 | 1.39E-03 | 4.28 |
| Thecc1EG025526 | Leucine-rich repeat containing protein,putative | 7.21 | -1.85 | 20.23 | 1.18E-03 | 4.26 |
| Thecc1EG008400 | Uncharacterized protein | -6.79 | -2.46 | -20.54 | 1.17E-03 | 4.23 |
| Thecc1EG022768 | Iaa-amino acid hydrolase 4 | 6.86 | 0.95 | 18.96 | 1.39E-03 | 4.23 |
| Thecc1EG026976 | Uncharacterized protein | -2.65 | 2.62 | -18.23 | 1.47E-03 | 4.22 |
| Thecc1EG029279 | P-loop containing nucleoside triphosphate hydrolases superfamily protein | 2.37 | 4.01 | 18.60 | 1.42E-03 | 4.19 |
| Thecc1EG005451 | Subtilase family protein | -2.39 | 3.17 | -18.25 | 1.47E-03 | 4.18 |
| Thecc1EG007030 | Catalytic,putative | -7.40 | -0.44 | -19.10 | 1.36E-03 | 4.16 |
| Thecc1EG002884 | Uncharacterized protein | 6.65 | -2.12 | 20.09 | 1.20E-03 | 4.16 |
| Thecc1EG025536 | LRR and NB-ARC domains-containing disease resistance protein,putative | -6.43 | -2.64 | -20.49 | 1.17E-03 | 4.15 |
| Thecc1EG004307 | Uncharacterized protein | 7.04 | -2.48 | 19.73 | 1.24E-03 | 4.15 |
| Thecc1EG015177 | AMP-dependent synthetase and ligase family protein | -1.86 | 4.92 | -18.85 | 1.39E-03 | 4.14 |
| Thecc1EG004162 | Uncharacterized protein | -6.91 | -1.87 | -19.70 | 1.24E-03 | 4.13 |
| Thecc1EG026978 | Uncharacterized protein | 2.63 | 2.76 | 17.78 | 1.56E-03 | 4.10 |
| Thecc1EG041231 | Uncharacterized protein | 1.81 | 3.54 | 18.16 | 1.47E-03 | 4.10 |
| Thecc1EG007657 | E3 ubiquitin-protein ligase RNF25 | -2.23 | 4.44 | -18.56 | 1.42E-03 | 4.10 |
| Thecc1EG025259 | Kinase superfamily protein,putative | -6.01 | -1.91 | -19.18 | 1.36E-03 | 4.10 |
| Thecc1EG001682 | SAUR family protein | -3.94 | 1.50 | -17.71 | 1.56E-03 | 4.09 |
| Thecc1EG027619 | Uncharacterized protein | -8.84 | -0.66 | -18.64 | 1.42E-03 | 4.08 |
| Thecc1EG024706 | Uncharacterized protein | -7.51 | -1.57 | -18.85 | 1.39E-03 | 4.08 |
| Thecc1EG044420 | Uncharacterized protein | -6.26 | 0.14 | -18.63 | 1.42E-03 | 4.07 |
| Thecc1EG025302 | Phosphoprotein phosphatase | -5.22 | 0.57 | -18.19 | 1.47E-03 | 4.07 |
| Thecc1EG041240 | Uncharacterized protein | 9.41 | 0.02 | 18.55 | 1.42E-03 | 4.07 |
| Thecc1EG014433 | Signal peptide peptidase | 1.98 | 3.69 | 18.06 | 1.50E-03 | 4.07 |
| Thecc1EG030589 | Nucleotide/sugar transporter family protein | 2.25 | 4.70 | 18.47 | 1.43E-03 | 4.07 |
| Thecc1EG025750 | ATPP2-A2,putative | -6.73 | -2.50 | -19.69 | 1.24E-03 | 4.06 |
| Thecc1EG031675 | Uncharacterized protein | -2.08 | 3.30 | -17.91 | 1.54E-03 | 4.06 |
| Thecc1EG032589 | Uncharacterized protein | 7.81 | -0.52 | 18.50 | 1.43E-03 | 4.05 |
| Thecc1EG044789 | Uncharacterized protein | 4.62 | -0.02 | 18.29 | 1.47E-03 | 4.03 |
| Thecc1EG022565 | Unknown | 8.75 | -0.31 | 18.27 | 1.47E-03 | 4.02 |
| Thecc1EG031221 | S-locus lectin protein kinase family protein | -1.80 | 6.52 | -18.82 | 1.39E-03 | 4.02 |
| Thecc1EG020093 | Unknown | -7.39 | -2.17 | -19.01 | 1.38E-03 | 4.00 |
| Thecc1EG047012 | Nucleotide-sensitive chloride conductance regulator (ICln) family protein | 1.62 | 5.09 | 18.43 | 1.44E-03 | 3.97 |
| Thecc1EG025928 | Calcium-dependent phospholipid-binding Copine family protein | 3.55 | 4.14 | 17.70 | 1.56E-03 | 3.97 |
| Thecc1EG017218 | Unknown | 5.10 | 1.14 | 17.18 | 1.70E-03 | 3.93 |
| Thecc1EG000721 | Zinc finger protein 7,putative | -3.82 | 2.59 | -17.03 | 1.74E-03 | 3.91 |
| Thecc1EG001591 | Retrotransposon protein,unclassified,putative | -6.13 | -2.79 | -18.86 | 1.39E-03 | 3.89 |
| Thecc1EG026594 | Kinase superfamily protein | 3.02 | 1.44 | 16.90 | 1.79E-03 | 3.89 |
| Thecc1EG016539 | Trehalase 1,putative | -6.59 | -2.57 | -18.18 | 1.47E-03 | 3.84 |
| Thecc1EG001776 | Uncharacterized protein | -9.05 | -0.80 | -17.37 | 1.64E-03 | 3.83 |
| Thecc1EG026328 | Unknown | -5.43 | -1.07 | -17.73 | 1.56E-03 | 3.82 |
| Thecc1EG018953 | PLC-like phosphodiesterases superfamily protein | -3.39 | 0.58 | -16.76 | 1.80E-03 | 3.81 |
| Thecc1EG002427 | RPM1-interacting protein 4 (RIN4) family protein | -1.73 | 5.14 | -17.90 | 1.54E-03 | 3.81 |
| Thecc1EG029112 | Nitrilase/cyanide hydratase and apolipoprotein N-acyltransferase family protein | -1.83 | 5.16 | -17.85 | 1.54E-03 | 3.79 |
| Thecc1EG020211 | Selenium-binding protein 1 | -1.85 | 5.33 | -17.87 | 1.54E-03 | 3.79 |
| Thecc1EG011277 | Vacuolar iron transporter 1 | -3.61 | 0.72 | -16.73 | 1.80E-03 | 3.79 |
| Thecc1EG026940 | GTP binding | -1.36 | 7.89 | -17.89 | 1.54E-03 | 3.78 |
| Thecc1EG042679 | Disease resistance protein RPP8 | 5.80 | 0.15 | 17.06 | 1.74E-03 | 3.76 |
| Thecc1EG043468 | Uncharacterized protein | -6.20 | -1.70 | -17.43 | 1.62E-03 | 3.76 |
| Thecc1EG042130 | Uncharacterized protein | 1.99 | 3.00 | 16.71 | 1.80E-03 | 3.75 |
| Thecc1EG004508 | Urease,putative | -1.28 | 4.96 | -17.66 | 1.56E-03 | 3.75 |
| Thecc1EG010738 | HXXXD-type acyl-transferase family protein,putative | -2.08 | 4.98 | -17.66 | 1.56E-03 | 3.75 |
| Thecc1EG019787 | Uncharacterized protein | -2.64 | 2.21 | -16.36 | 1.92E-03 | 3.73 |
| Thecc1EG004118 | Uncharacterized protein | -1.65 | 5.46 | -17.70 | 1.56E-03 | 3.71 |
| Thecc1EG037613 | Uncharacterized protein | -4.32 | -0.01 | -16.84 | 1.80E-03 | 3.71 |
| Thecc1EG031000 | Unknown | 2.07 | 5.06 | 17.46 | 1.62E-03 | 3.70 |
| Thecc1EG016137 | D-isomer specific 2-hydroxyacid dehydrogenase family protein,putative | 1.89 | 2.96 | 16.37 | 1.92E-03 | 3.64 |
| Thecc1EG005766 | Uncharacterized protein | -2.80 | 1.15 | -16.03 | 2.01E-03 | 3.64 |
| Thecc1EG045197 | Uncharacterized protein | -5.21 | -0.77 | -16.73 | 1.80E-03 | 3.64 |
| Thecc1EG033788 | UDP-glucosyl transferase 73B5,putative | 3.35 | 1.17 | 15.99 | 2.01E-03 | 3.63 |
| Thecc1EG046064 | Cc-nbs-lrr resistance-like protein | -2.47 | 3.12 | -16.28 | 1.93E-03 | 3.62 |
| Thecc1EG001949 | Glutathione S-transferase PHI 9 | -2.20 | 6.56 | -17.43 | 1.62E-03 | 3.61 |
| Thecc1EG030935 | Uncharacterized protein | 1.39 | 6.10 | 17.44 | 1.62E-03 | 3.60 |
| Thecc1EG020163 | Wall associated kinase-like 6,putative | -3.13 | 2.07 | -15.93 | 2.02E-03 | 3.60 |
| Thecc1EG042967 | Plant protein 1589 of Uncharacterized protein function | 2.20 | 3.17 | 16.41 | 1.91E-03 | 3.59 |
| Thecc1EG014284 | Uncharacterized protein | 1.17 | 5.20 | 17.22 | 1.70E-03 | 3.57 |
| Thecc1EG005466 | D-isomer specific 2-hydroxyacid dehydrogenase family protein,putative | 2.56 | 4.66 | 16.74 | 1.80E-03 | 3.56 |
| Thecc1EG005767 | Uncharacterized protein | 9.03 | -0.94 | 16.19 | 1.95E-03 | 3.56 |
| Thecc1EG020161 | Uncharacterized protein | -7.05 | -2.35 | -16.69 | 1.80E-03 | 3.55 |
| Thecc1EG041656 | Uncharacterized protein | 1.54 | 4.15 | 16.77 | 1.80E-03 | 3.54 |
| Thecc1EG013244 | DNA/RNA polymerases superfamily protein,putative | -4.19 | 0.07 | -15.97 | 2.01E-03 | 3.52 |
| Thecc1EG025176 | UDP-glucosyltransferase,putative | -2.72 | 1.46 | -15.62 | 2.15E-03 | 3.52 |
| Thecc1EG015612 | UDP-Glycosyltransferase superfamily protein | -1.19 | 6.58 | -17.11 | 1.73E-03 | 3.51 |
| Thecc1EG029528 | Carbohydrate-binding X8 domain superfamily protein,putative | -3.08 | 0.92 | -15.59 | 2.15E-03 | 3.50 |
| Thecc1EG022732 | Alpha/beta-Hydrolases superfamily protein | 4.33 | 0.68 | 15.72 | 2.11E-03 | 3.50 |
| Thecc1EG025495 | MLP-like protein 423,putative | -2.82 | 0.99 | -15.56 | 2.15E-03 | 3.50 |
| Thecc1EG004617 | Tetratricopeptide repeat-containing protein | 1.40 | 4.19 | 16.51 | 1.88E-03 | 3.48 |
| Thecc1EG029703 | General regulatory factor 12,IOTA | 1.12 | 5.32 | 17.03 | 1.74E-03 | 3.48 |
| Thecc1EG025768 | MATE efflux family protein | 1.72 | 2.82 | 15.94 | 2.02E-03 | 3.45 |
| Thecc1EG045041 | Receptor like protein 15,putative | -2.52 | 2.35 | -15.58 | 2.15E-03 | 3.45 |
| Thecc1EG027577 | Unknown | -4.39 | -0.43 | -15.87 | 2.04E-03 | 3.45 |
| Thecc1EG027890 | Leucine-rich repeat family protein / protein kinase family protein | -2.33 | 3.88 | -16.19 | 1.95E-03 | 3.45 |
| Thecc1EG031841 | Uncharacterized protein | -3.23 | 1.81 | -15.41 | 2.18E-03 | 3.45 |
| Thecc1EG034136 | Walls Are Thin 1 | -1.68 | 5.33 | -16.69 | 1.80E-03 | 3.44 |
| Thecc1EG012403 | Kinase superfamily protein | -6.10 | -2.27 | -16.32 | 1.92E-03 | 3.43 |
| Thecc1EG007798 | 3R-linalool synthase | -4.22 | 6.92 | -16.61 | 1.83E-03 | 3.42 |
| Thecc1EG030053 | Uncharacterized protein | -5.23 | -0.88 | -15.99 | 2.01E-03 | 3.42 |
| Thecc1EG019799 | Trichome birefringence-like 43,putative | 3.33 | 0.39 | 15.33 | 2.21E-03 | 3.41 |
| Thecc1EG008823 | Uncharacterized protein | 6.46 | -1.69 | 15.80 | 2.07E-03 | 3.41 |
| Thecc1EG011906 | NADP-dependent oxidoreductase P1 | 1.18 | 6.54 | 16.77 | 1.80E-03 | 3.38 |
| Thecc1EG025218 | Senescence-related gene 1 | 6.59 | -0.45 | 15.44 | 2.18E-03 | 3.36 |
| Thecc1EG005344 | Seven transmembrane domain-containing tyrosine-protein kinase 1 | -1.79 | 3.26 | -15.50 | 2.18E-03 | 3.34 |
| Thecc1EG021086 | Pyruvate orthophosphate dikinase | -1.33 | 5.30 | -16.30 | 1.93E-03 | 3.31 |
| Thecc1EG016876 | Oxidoreductase,zinc-binding dehydrogenase family protein | -2.42 | 3.68 | -15.80 | 2.07E-03 | 3.31 |
| Thecc1EG027238 | Cc-nbs-lrr resistance protein,putative | -7.15 | -1.74 | -15.41 | 2.18E-03 | 3.30 |
| Thecc1EG005671 | Unknown | -2.74 | 3.35 | -15.48 | 2.18E-03 | 3.30 |
| Thecc1EG004291 | Bax inhibitor-1 family protein | 1.38 | 4.60 | 16.04 | 2.01E-03 | 3.29 |
| Thecc1EG004107 | Poly(ADP-ribose) polymerase 2 | 1.17 | 6.15 | 16.48 | 1.88E-03 | 3.28 |
| Thecc1EG027121 | Disease resistance protein,putative | 7.79 | 0.27 | 15.06 | 2.29E-03 | 3.27 |
| Thecc1EG004776 | Uncharacterized protein | 4.50 | -0.48 | 15.07 | 2.29E-03 | 3.23 |
| Thecc1EG046703 | Flower,cultured cell,putative | -1.27 | 6.46 | -16.35 | 1.92E-03 | 3.23 |
| Thecc1EG046742 | Cytochrome P450,family 706,subfamily A,polypeptide 4,putative | 3.48 | 3.62 | 15.21 | 2.24E-03 | 3.22 |
| Thecc1EG004591 | Glutathione S-transferase phi 8 | -1.47 | 5.50 | -16.15 | 1.96E-03 | 3.20 |
| Thecc1EG014537 | S-adenosyl-L-homocysteine hydrolase | -1.12 | 5.93 | -16.23 | 1.94E-03 | 3.20 |
| Thecc1EG031051 | Uncharacterized protein | 5.55 | -0.58 | 14.88 | 2.37E-03 | 3.20 |
| Thecc1EG046793 | Iron regulated 1 protein | 2.69 | 4.07 | 15.44 | 2.18E-03 | 3.19 |
| Thecc1EG020297 | Uncharacterized protein | -3.94 | 0.48 | -14.60 | 2.41E-03 | 3.18 |
| Thecc1EG019628 | Methylenetetrahydrofolate reductase family protein | -1.54 | 4.59 | -15.56 | 2.15E-03 | 3.18 |
| Thecc1EG046689 | P-loop containing nucleoside triphosphate hydrolases superfamily protein,putative | -1.19 | 5.10 | -15.98 | 2.01E-03 | 3.18 |
| Thecc1EG004117 | Uncharacterized protein | 5.19 | 0.64 | 14.55 | 2.41E-03 | 3.17 |
| Thecc1EG042129 | High affinity nitrate transporter 2.6 | -6.66 | -1.73 | -14.97 | 2.32E-03 | 3.17 |
| Thecc1EG004732 | Heat shock protein binding protein | 2.72 | 1.33 | 14.75 | 2.41E-03 | 3.17 |
| Thecc1EG045199 | Uncharacterized protein | -6.36 | 1.83 | -14.48 | 2.42E-03 | 3.17 |
| Thecc1EG029406 | Uncharacterized protein | -7.20 | -0.70 | -14.71 | 2.41E-03 | 3.16 |
| Thecc1EG015836 | Serine/threonine-protein kinase SAPK3 | -2.45 | 1.67 | -14.52 | 2.42E-03 | 3.16 |
| Thecc1EG024669 | Uncharacterized protein | 9.07 | 0.02 | 14.60 | 2.41E-03 | 3.14 |
| Thecc1EG025347 | Single-stranded nucleic acid binding R3H protein,putative | -1.63 | 4.13 | -15.49 | 2.18E-03 | 3.14 |
| Thecc1EG017618 | Glutathione S-transferase F11 | -7.54 | 1.21 | -14.49 | 2.42E-03 | 3.14 |
| Thecc1EG005401 | Uncharacterized protein | 2.10 | 3.77 | 15.11 | 2.29E-03 | 3.12 |
| Thecc1EG008065 | Regulator of Vps4 activity in the MVB pathway protein,putative | -2.43 | 1.50 | -14.32 | 2.49E-03 | 3.10 |
| Thecc1EG001755 | Uncharacterized protein | 6.07 | -1.88 | 14.61 | 2.41E-03 | 3.09 |
| Thecc1EG005389 | NAD(P)-binding Rossmann-fold superfamily protein,putative | -1.69 | 3.27 | -14.88 | 2.37E-03 | 3.08 |
| Thecc1EG008881 | Disease resistance RPS5,putative-like protein | 4.82 | 1.09 | 14.20 | 2.53E-03 | 3.08 |
| Thecc1EG041242 | Uncharacterized protein | 6.28 | -1.79 | 14.61 | 2.41E-03 | 3.07 |
| Thecc1EG021964 | Uncharacterized protein | 5.12 | -1.60 | 14.64 | 2.41E-03 | 3.07 |
| Thecc1EG038207 | Uncharacterized protein | 8.03 | -0.66 | 14.35 | 2.47E-03 | 3.07 |
| Thecc1EG045936 | Uncharacterized protein | -4.61 | 3.25 | -14.48 | 2.42E-03 | 3.06 |
| Thecc1EG005893 | Uncharacterized protein | -2.45 | 2.12 | -14.22 | 2.52E-03 | 3.04 |
| Thecc1EG006471 | Flavin-dependent monooxygenase 1 | 2.53 | 1.95 | 14.23 | 2.52E-03 | 3.04 |
| Thecc1EG004585 | Soybean gene regulated by cold-2-like protein | 1.84 | 3.35 | 14.67 | 2.41E-03 | 3.03 |
| Thecc1EG033249 | Unknown | -1.35 | 7.43 | -15.64 | 2.15E-03 | 3.03 |
| Thecc1EG025740 | Uncharacterized protein | -2.57 | 1.21 | -14.07 | 2.60E-03 | 3.03 |
| Thecc1EG018560 | Amino acid transporter | -4.28 | -0.32 | -14.37 | 2.47E-03 | 3.02 |
| Thecc1EG042672 | Uncharacterized protein | -1.63 | 3.47 | -14.74 | 2.41E-03 | 3.01 |
| Thecc1EG027259 | Uncharacterized protein | -6.08 | -2.29 | -14.39 | 2.46E-03 | 3.01 |
| Thecc1EG025625 | Cytochrome P450,family 87,subfamily A,polypeptide 6 | -1.58 | 4.62 | -15.29 | 2.21E-03 | 3.00 |
| Thecc1EG030590 | Uncharacterized protein | 3.89 | 6.25 | 15.30 | 2.21E-03 | 2.98 |
| Thecc1EG045380 | Retrotransposon protein,unclassified,putative | 6.23 | -2.33 | 14.24 | 2.52E-03 | 2.98 |
| Thecc1EG029251 | Pectin methylesterase 3 | 1.51 | 8.03 | 15.46 | 2.18E-03 | 2.97 |
| Thecc1EG020370 | Vesicle-associated membrane protein 721,VAMP7B | -3.06 | 0.54 | -13.90 | 2.66E-03 | 2.97 |
| Thecc1EG024797 | Peptidase S41 family protein | 1.45 | 3.42 | 14.67 | 2.41E-03 | 2.97 |
| Thecc1EG029714 | ARM repeat superfamily protein | 2.48 | 2.42 | 14.23 | 2.52E-03 | 2.97 |
| Thecc1EG031532 | Nbs-lrr resistance protein | -3.12 | 0.24 | -13.84 | 2.68E-03 | 2.95 |
| Thecc1EG034439 | DNA binding protein,putative | 1.46 | 4.88 | 15.04 | 2.29E-03 | 2.95 |
| Thecc1EG046698 | NAD(P)-binding Rossmann-fold superfamily protein,putative | -1.37 | 7.65 | -15.33 | 2.21E-03 | 2.94 |
| Thecc1EG021572 | NAD(P)-binding Rossmann-fold superfamily protein | 2.44 | 5.05 | 15.12 | 2.29E-03 | 2.93 |
| Thecc1EG031014 | Unknown | 5.06 | -0.38 | 13.93 | 2.65E-03 | 2.93 |
| Thecc1EG013887 | Calcineurin-like metallo-phosphoesterase superfamily protein | -1.05 | 5.20 | -15.31 | 2.21E-03 | 2.91 |
| Thecc1EG007906 | HXXXD-type acyl-transferase family protein,putative | -2.39 | 2.64 | -13.87 | 2.66E-03 | 2.90 |
| Thecc1EG016503 | Cellulose synthase like G3,putative | -3.78 | 4.17 | -14.45 | 2.42E-03 | 2.89 |
| Thecc1EG020900 | NAD(P)-linked oxidoreductase superfamily protein | 1.32 | 4.07 | 14.67 | 2.41E-03 | 2.89 |
| Thecc1EG039407 | Uncharacterized protein | -2.84 | 0.49 | -13.64 | 2.77E-03 | 2.89 |
| Thecc1EG038608 | BNR/Asp-box repeat family protein | 1.05 | 5.49 | 15.26 | 2.22E-03 | 2.89 |
| Thecc1EG031641 | CC-NBS-LRR class disease resistance protein | -7.08 | -1.53 | -13.87 | 2.66E-03 | 2.89 |
| Thecc1EG043390 | Non-symbiotic hemoglobin 2 | 2.18 | 1.54 | 13.74 | 2.74E-03 | 2.88 |
| Thecc1EG016811 | Uncharacterized protein | -1.33 | 4.58 | -14.99 | 2.32E-03 | 2.88 |
| Thecc1EG022044 | Aluminum sensitive 3 | 2.26 | 3.71 | 14.63 | 2.41E-03 | 2.87 |
| Thecc1EG024849 | Uncharacterized protein | 6.04 | -2.43 | 13.96 | 2.63E-03 | 2.87 |
| Thecc1EG028917 | Uncharacterized protein | -7.12 | -1.76 | -13.79 | 2.71E-03 | 2.87 |
| Thecc1EG005130 | Integrase-type DNA-binding superfamily protein | 3.13 | 1.49 | 13.62 | 2.77E-03 | 2.86 |
| Thecc1EG001080 | UDP-glucosyl transferase 85A2 | 1.98 | 6.07 | 15.06 | 2.29E-03 | 2.86 |
| Thecc1EG005585 | Endoplasmic reticulum-type calcium-transporting ATPase 3 | -1.38 | 4.21 | -14.58 | 2.41E-03 | 2.84 |
| Thecc1EG037317 | Uncharacterized protein | 1.16 | 6.09 | 15.21 | 2.24E-03 | 2.84 |
| Thecc1EG024673 | Cc-nbs-lrr resistance protein,putative | 2.97 | 4.98 | 14.68 | 2.41E-03 | 2.83 |
| Thecc1EG026502 | NB-ARC domain-containing disease resistance protein,putative | -1.18 | 5.29 | -15.07 | 2.29E-03 | 2.83 |
| Thecc1EG041365 | Unknown | -6.90 | -1.09 | -13.66 | 2.77E-03 | 2.83 |
| Thecc1EG042661 | RAB GTPase A2B | -7.13 | -1.75 | -13.64 | 2.77E-03 | 2.83 |
| Thecc1EG045236 | Ankyrin repeat family protein | -1.81 | 3.37 | -14.14 | 2.58E-03 | 2.82 |
| Thecc1EG025300 | Phosphoprotein phosphatase | -1.91 | 3.30 | -14.07 | 2.60E-03 | 2.81 |
| Thecc1EG020393 | Uncharacterized protein | -2.47 | 1.30 | -13.47 | 2.86E-03 | 2.80 |
| Thecc1EG022100 | Pre-mRNA splicing factor-related | -5.28 | -2.16 | -13.68 | 2.77E-03 | 2.80 |
| Thecc1EG007766 | Cyclin p2,1 | 1.55 | 4.99 | 14.84 | 2.38E-03 | 2.79 |
| Thecc1EG020874 | Uncharacterized protein | -1.44 | 10.32 | -14.54 | 2.41E-03 | 2.79 |
| Thecc1EG040374 | U-box domain-containing protein 42,putative | -8.03 | -0.89 | -13.43 | 2.88E-03 | 2.78 |
| Thecc1EG025454 | UDP-glycosyltransferase 73C5 | -1.45 | 3.04 | -14.10 | 2.59E-03 | 2.78 |
| Thecc1EG037326 | Uncharacterized protein | 1.02 | 5.43 | 14.96 | 2.32E-03 | 2.78 |
| Thecc1EG002058 | Uncharacterized protein | -7.45 | -2.16 | -13.50 | 2.84E-03 | 2.78 |
| Thecc1EG043459 | Phosphorylase superfamily protein | 1.54 | 4.28 | 14.46 | 2.42E-03 | 2.77 |
| Thecc1EG036608 | Laccase 14,putative | 1.23 | 10.18 | 14.45 | 2.42E-03 | 2.75 |
| Thecc1EG043228 | NBS type disease resistance protein,putative | -1.77 | 2.55 | -13.62 | 2.77E-03 | 2.75 |
| Thecc1EG021991 | Histone superfamily protein | 1.06 | 4.99 | 14.75 | 2.41E-03 | 2.75 |
| Thecc1EG025920 | Cellulose synthase like E1 | 1.20 | 5.27 | 14.75 | 2.41E-03 | 2.73 |
| Thecc1EG009578 | Uncharacterized protein | 6.93 | -1.03 | 13.26 | 2.99E-03 | 2.73 |
| Thecc1EG000947 | Geraniol dehydrogenase 1 | 2.14 | 1.78 | 13.66 | 2.77E-03 | 2.73 |
| Thecc1EG020851 | 2-oxoglutarate (2OG) and Fe(II)-dependent oxygenase superfamily protein,putative | -1.53 | 4.72 | -14.51 | 2.42E-03 | 2.71 |
| Thecc1EG002627 | Glutamate receptor 1.1,putative | -6.19 | -2.22 | -13.45 | 2.87E-03 | 2.71 |
| Thecc1EG030156 | Gibberellin 2-oxidase 8 | 1.73 | 2.55 | 13.64 | 2.77E-03 | 2.70 |
| Thecc1EG031842 | Receptor like protein 33 | -3.60 | -0.07 | -13.11 | 3.06E-03 | 2.70 |
| Thecc1EG010574 | GDSL-like Lipase/Acylhydrolase superfamily protein | 2.48 | 2.25 | 13.28 | 2.98E-03 | 2.69 |
| Thecc1EG041163 | Glycosyl hydrolase family protein | 0.99 | 5.70 | 14.62 | 2.41E-03 | 2.66 |
| Thecc1EG031531 | F8L10.3 protein | -5.54 | -2.54 | -13.39 | 2.91E-03 | 2.66 |
| Thecc1EG044848 | Leucine-rich repeat receptor-like protein kinase family protein,putative | -3.85 | -1.29 | -13.20 | 3.02E-03 | 2.65 |
| Thecc1EG005037 | Pyruvate dehydrogenase E1 alpha,ALPHA | 1.07 | 5.87 | 14.68 | 2.41E-03 | 2.64 |
| Thecc1EG045132 | Uncharacterized protein | -2.92 | 1.61 | -13.05 | 3.10E-03 | 2.64 |
| Thecc1EG033320 | Uncharacterized protein | -1.77 | 2.28 | -13.30 | 2.97E-03 | 2.64 |
| Thecc1EG033954 | Cytochrome P450,putative | 1.29 | 6.15 | 14.64 | 2.41E-03 | 2.63 |
| Thecc1EG033762 | N-terminal nucleophile aminohydrolases (Ntn hydrolases) superfamily protein | 1.86 | 7.60 | 14.55 | 2.41E-03 | 2.63 |
| Thecc1EG015210 | Divalent ion symporter | -4.27 | -1.32 | -13.08 | 3.08E-03 | 2.62 |
| Thecc1EG031523 | Nbs-lrr resistance-like protein | 5.61 | -2.64 | 13.14 | 3.05E-03 | 2.60 |
| Thecc1EG036843 | DNA binding protein,putative | 4.93 | -1.45 | 12.97 | 3.15E-03 | 2.59 |
| Thecc1EG010395 | Uncharacterized protein | 1.45 | 3.64 | 13.68 | 2.77E-03 | 2.58 |
| Thecc1EG037497 | Photosystem I light harvesting complex gene 6 | 1.71 | 4.42 | 13.88 | 2.66E-03 | 2.58 |
| Thecc1EG029276 | P-loop containing nucleoside triphosphate hydrolases superfamily protein | 3.95 | -0.27 | 12.77 | 3.27E-03 | 2.57 |
| Thecc1EG020109 | Unknown | -2.13 | 0.61 | -13.25 | 3.00E-03 | 2.56 |
| Thecc1EG041798 | ZRT/IRT-like protein 2 | -1.32 | 4.03 | -13.98 | 2.63E-03 | 2.55 |
| Thecc1EG022647 | Cell wall invertase 2 | 7.84 | -1.54 | 12.74 | 3.30E-03 | 2.55 |
| Thecc1EG000609 | Red chlorophyll catabolite reductase,putative | 1.21 | 4.36 | 14.01 | 2.63E-03 | 2.55 |
| Thecc1EG046760 | NAD(P)-linked oxidoreductase superfamily protein | -2.07 | 1.61 | -12.81 | 3.27E-03 | 2.54 |
| Thecc1EG019880 | Malectin/receptor protein kinase family protein | 4.49 | -0.51 | 12.72 | 3.30E-03 | 2.54 |
| Thecc1EG013430 | Uncharacterized protein | -3.62 | -1.12 | -12.79 | 3.27E-03 | 2.52 |
| Thecc1EG016526 | Fiber protein Fb17 | 2.37 | 3.47 | 13.54 | 2.83E-03 | 2.52 |
| Thecc1EG041436 | CER1 protein | -2.53 | 3.36 | -13.37 | 2.91E-03 | 2.52 |
| Thecc1EG031189 | Unknown | 3.83 | 0.43 | 12.57 | 3.35E-03 | 2.51 |
| Thecc1EG020633 | Late embryogenesis abundant protein D-34 | -2.99 | 0.90 | -12.58 | 3.34E-03 | 2.51 |
| Thecc1EG005668 | Uncharacterized protein | 2.56 | 1.46 | 12.69 | 3.31E-03 | 2.50 |
| Thecc1EG045126 | Cytochrome P450 | 3.23 | 0.19 | 12.56 | 3.35E-03 | 2.50 |
| Thecc1EG004713 | Pollen Ole e 1 allergen and extensin family protein | 3.62 | -0.31 | 12.52 | 3.38E-03 | 2.49 |
| Thecc1EG042630 | RING/FYVE/PHD zinc finger superfamily protein | -1.67 | 5.27 | -14.02 | 2.63E-03 | 2.48 |
| Thecc1EG031595 | Squamosa promoter binding protein-like 3 | 1.41 | 6.00 | 14.23 | 2.52E-03 | 2.48 |
| Thecc1EG004697 | Glycosyl hydrolase 9B13 | -1.26 | 4.39 | -13.83 | 2.68E-03 | 2.48 |
| Thecc1EG032491 | Uncharacterized protein | -7.82 | 0.14 | -12.49 | 3.39E-03 | 2.47 |
| Thecc1EG010240 | Unknown | 6.29 | -2.30 | 12.62 | 3.32E-03 | 2.47 |
| Thecc1EG026235 | Serine-threonine protein kinase,plant-type,putative | -4.66 | -0.05 | -12.45 | 3.40E-03 | 2.47 |
| Thecc1EG042580 | S-adenosyl-L-methionine-dependent methyltransferases superfamily protein | 2.60 | 8.22 | 13.96 | 2.63E-03 | 2.46 |
| Thecc1EG030693 | Multidrug resistance-associated protein 3 | -2.91 | 2.28 | -12.73 | 3.30E-03 | 2.46 |
| Thecc1EG015429 | Uncharacterized protein | -3.23 | 0.02 | -12.45 | 3.40E-03 | 2.46 |
| Thecc1EG005026 | Quercetin 3-O-methyltransferase 1,putative | 0.97 | 5.43 | 14.12 | 2.59E-03 | 2.46 |
| Thecc1EG037855 | Uncharacterized protein | 1.17 | 5.00 | 13.96 | 2.63E-03 | 2.44 |
| Thecc1EG042900 | Cysteine-rich RLK (RECEPTOR-like protein kinase) 8 | 3.09 | 0.75 | 12.43 | 3.42E-03 | 2.44 |
| Thecc1EG011068 | Uncharacterized protein | 2.15 | 2.60 | 12.80 | 3.27E-03 | 2.43 |
| Thecc1EG041034 | Beta glucosidase 11,putative | -1.31 | 6.11 | -14.11 | 2.59E-03 | 2.42 |
| Thecc1EG045575 | Uncharacterized protein | 1.80 | 3.14 | 12.95 | 3.15E-03 | 2.42 |
| Thecc1EG042971 | Uncharacterized protein | 1.46 | 3.18 | 13.04 | 3.10E-03 | 2.40 |
| Thecc1EG004971 | Peptidyl-prolyl cis-trans isomerase | 1.08 | 5.60 | 14.00 | 2.63E-03 | 2.40 |
| Thecc1EG016648 | Isoflavone reductase | 2.41 | 4.10 | 13.38 | 2.91E-03 | 2.39 |
| Thecc1EG019419 | GRAS domain protein,putative | 3.31 | 1.08 | 12.29 | 3.48E-03 | 2.38 |
| Thecc1EG032175 | BED finger-nbs-lrr resistance-like protein | -3.35 | 1.08 | -12.28 | 3.48E-03 | 2.38 |
| Thecc1EG027356 | Uncharacterized protein | 3.80 | -0.21 | 12.21 | 3.55E-03 | 2.38 |
| Thecc1EG004647 | Beta glucosidase 11 | -2.28 | 2.34 | -12.63 | 3.32E-03 | 2.37 |
| Thecc1EG011234 | Uncharacterized protein | 4.13 | -1.44 | 12.33 | 3.47E-03 | 2.37 |
| Thecc1EG032808 | Peroxidase superfamily protein,putative | 1.50 | 3.80 | 13.22 | 3.02E-03 | 2.37 |
| Thecc1EG042842 | F-box family protein,putative | 5.92 | 0.04 | 12.17 | 3.56E-03 | 2.36 |
| Thecc1EG008447 | Uncharacterized protein | 2.98 | 1.30 | 12.28 | 3.48E-03 | 2.36 |
| Thecc1EG019143 | Cytochrome P450,family 81,subfamily D,polypeptide 8,putative | -2.36 | 0.63 | -12.18 | 3.56E-03 | 2.35 |
| Thecc1EG014362 | Nucleic acid binding,ATP-dependent helicases,ATP binding,helicases,ATP-dependent helicases | -1.01 | 4.91 | -13.69 | 2.77E-03 | 2.34 |
| Thecc1EG042215 | UDP-Glycosyltransferase superfamily protein,putative | -1.15 | 5.83 | -13.89 | 2.66E-03 | 2.34 |
| Thecc1EG020686 | Iq-domain 31 | -1.95 | 1.97 | -12.35 | 3.46E-03 | 2.34 |
| Thecc1EG045093 | LRR and NB-ARC domains-containing disease resistance protein,putative | 4.39 | 1.17 | 12.16 | 3.56E-03 | 2.33 |
| Thecc1EG014844 | Oleosin 1 | -7.55 | 0.05 | -12.09 | 3.63E-03 | 2.33 |
| Thecc1EG020576 | Immunoglobulin G-binding protein H | 2.94 | 2.82 | 12.68 | 3.31E-03 | 2.32 |
| Thecc1EG027582 | S-adenosyl-L-methionine-dependent methyltransferases superfamily protein,putative | -3.13 | 0.48 | -12.06 | 3.65E-03 | 2.32 |
| Thecc1EG010330 | Uncharacterized protein | 1.09 | 4.64 | 13.37 | 2.91E-03 | 2.31 |
| Thecc1EG045393 | Cysteine/Histidine-rich C1 domain family protein,putative | 2.05 | 1.56 | 12.28 | 3.48E-03 | 2.31 |
| Thecc1EG021412 | Poly(A) polymerase 3,putative | -2.03 | 2.46 | -12.46 | 3.40E-03 | 2.31 |
| Thecc1EG028963 | Hydroxyproline-rich glycoprotein family protein,putative | -1.47 | 3.63 | -12.90 | 3.20E-03 | 2.31 |
| Thecc1EG012130 | Ankyrin repeat family protein | -3.17 | -0.41 | -11.96 | 3.74E-03 | 2.28 |
| Thecc1EG042584 | S-adenosyl-L-methionine-dependent methyltransferases superfamily protein | 2.36 | 8.01 | 13.52 | 2.83E-03 | 2.27 |
| Thecc1EG029153 | Uncharacterized protein | -1.16 | 4.23 | -13.20 | 3.02E-03 | 2.26 |
| Thecc1EG041976 | DNA-directed RNA polymerase | -2.05 | 2.06 | -12.16 | 3.56E-03 | 2.26 |
| Thecc1EG001569 | PAD4,putative | 1.64 | 2.85 | 12.53 | 3.38E-03 | 2.25 |
| Thecc1EG031405 | Nbs-lrr resistance protein | 2.07 | 1.39 | 12.08 | 3.63E-03 | 2.25 |
| Thecc1EG032141 | Uncharacterized protein | -3.69 | -0.42 | -11.90 | 3.78E-03 | 2.25 |
| Thecc1EG005048 | IQ-domain 17 | -3.88 | 0.76 | -11.91 | 3.77E-03 | 2.24 |
| Thecc1EG017683 | Uncharacterized protein | 6.97 | -1.44 | 11.86 | 3.80E-03 | 2.24 |
| Thecc1EG031963 | Uncharacterized protein | -1.64 | 2.46 | -12.32 | 3.47E-03 | 2.23 |
| Thecc1EG021880 | Ribulose bisphosphate carboxylase/oxygenase activase 1 | 6.49 | 0.40 | 11.83 | 3.80E-03 | 2.23 |
| Thecc1EG004587 | Transducin/WD40 repeat-like superfamily protein | 0.87 | 5.96 | 13.60 | 2.78E-03 | 2.22 |
| Thecc1EG030064 | 3S-linalool/(E)-nerolidol /(E,E)-geranyl linalool synthase | -6.62 | -2.01 | -11.83 | 3.80E-03 | 2.21 |
| Thecc1EG016655 | Acyl-CoA N-acyltransferases superfamily protein,putative | 1.04 | 5.74 | 13.54 | 2.83E-03 | 2.20 |
| Thecc1EG009629 | Uncharacterized protein | 2.82 | 0.04 | 11.73 | 3.86E-03 | 2.19 |
| Thecc1EG014712 | Translocase inner membrane subunit 8 | 1.30 | 5.71 | 13.48 | 2.85E-03 | 2.19 |
| Thecc1EG046028 | TMV resistance protein N | -3.83 | -1.33 | -11.84 | 3.80E-03 | 2.19 |
| Thecc1EG030469 | Seed imbibition 2 | 3.56 | -0.16 | 11.72 | 3.86E-03 | 2.19 |
| Thecc1EG004938 | ATP binding cassette subfamily B4 | -1.33 | 3.47 | -12.64 | 3.32E-03 | 2.19 |
| Thecc1EG005465 | D-isomer specific 2-hydroxyacid dehydrogenase family protein | 1.87 | 4.41 | 13.03 | 3.10E-03 | 2.18 |
| Thecc1EG031331 | Uncharacterized protein | -2.40 | 1.10 | -11.85 | 3.80E-03 | 2.16 |
| Thecc1EG021966 | Unknown | 1.06 | 4.56 | 13.18 | 3.03E-03 | 2.16 |
| Thecc1EG005240 | Uncharacterized protein | -2.00 | 1.43 | -11.85 | 3.80E-03 | 2.16 |
| Thecc1EG033946 | Cytochrome P450 | 4.53 | 2.07 | 11.95 | 3.75E-03 | 2.15 |
| Thecc1EG011437 | O-fucosyltransferase family protein | -5.16 | -0.81 | -11.61 | 3.97E-03 | 2.15 |
| Thecc1EG028887 | Phosphatase 2C family protein | 4.53 | -1.12 | 11.65 | 3.93E-03 | 2.14 |
| Thecc1EG021184 | Cullin 1,putative | -2.78 | -0.44 | -11.59 | 3.98E-03 | 2.14 |
| Thecc1EG020372 | Vesicle-associated membrane 721,VAMP7B-like protein | 2.13 | 3.58 | 12.48 | 3.39E-03 | 2.13 |
| Thecc1EG030825 | RNAse THREE-like protein 2 | -1.03 | 4.89 | -13.13 | 3.05E-03 | 2.12 |
| Thecc1EG044476 | No pollen germination related 2,putative | 6.00 | -2.46 | 11.58 | 3.99E-03 | 2.11 |
| Thecc1EG015656 | YELLOW STRIPE like 1 | 2.63 | 5.84 | 13.17 | 3.03E-03 | 2.11 |
| Thecc1EG015756 | Single hybrid motif superfamily protein | 1.76 | 4.67 | 12.87 | 3.21E-03 | 2.10 |
| Thecc1EG016495 | Uncharacterized protein | 1.89 | 5.97 | 13.20 | 3.02E-03 | 2.10 |
| Thecc1EG018553 | Uncharacterized protein | -1.64 | 4.52 | -12.59 | 3.34E-03 | 2.10 |
| Thecc1EG026926 | Glycosyl hydrolases family 31 protein | -2.94 | -0.86 | -11.47 | 4.07E-03 | 2.09 |
| Thecc1EG004494 | Alpha/beta-Hydrolases superfamily protein | -1.34 | 5.15 | -13.08 | 3.08E-03 | 2.08 |
| Thecc1EG006190 | Embryogenesis-associated protein | -3.26 | -1.12 | -11.46 | 4.07E-03 | 2.07 |
| Thecc1EG037301 | Glycosyl hydrolases family 32 protein | -1.93 | 1.86 | -11.74 | 3.86E-03 | 2.06 |
| Thecc1EG021241 | Uncharacterized protein | -3.90 | 2.60 | -11.78 | 3.84E-03 | 2.06 |
| Thecc1EG010294 | Nodulin MtN21 /EamA-like transporter family protein | 3.63 | -0.41 | 11.36 | 4.18E-03 | 2.05 |
| Thecc1EG022064 | Uncharacterized protein | 3.08 | 3.54 | 12.20 | 3.55E-03 | 2.05 |
| Thecc1EG008054 | O-methyltransferase | 3.40 | 5.19 | 12.64 | 3.32E-03 | 2.04 |
| Thecc1EG014411 | NB-ARC domain-containing disease resistance protein,putative | 5.62 | -1.85 | 11.37 | 4.17E-03 | 2.04 |
| Thecc1EG025918 | Cellulose synthase like E1 | 3.22 | -0.80 | 11.36 | 4.18E-03 | 2.04 |
| Thecc1EG041417 | Uncharacterized protein | 2.18 | 1.15 | 11.83 | 3.80E-03 | 2.04 |
| Thecc1EG038282 | CHY-type/CTCHY-type/RING-type Zinc finger protein | -1.02 | 9.03 | -12.91 | 3.19E-03 | 2.03 |
| Thecc1EG011280 | Caffeoyl-CoA 3-O-methyltransferase | -3.22 | 1.88 | -11.58 | 3.98E-03 | 2.03 |
| Thecc1EG016570 | Uncharacterized protein | 3.29 | -0.61 | 11.33 | 4.19E-03 | 2.02 |
| Thecc1EG022268 | Methionine adenosyltransferase 3 | -0.88 | 5.86 | -13.13 | 3.05E-03 | 2.02 |
| Thecc1EG037624 | Membrane bound O-acyl transferase (MBOAT) family protein | -1.39 | 5.70 | -13.03 | 3.10E-03 | 2.02 |
| Thecc1EG014501 | Cytochrome P450 71A1 | -1.12 | 4.03 | -12.58 | 3.34E-03 | 2.01 |
| Thecc1EG033772 | Unknown | -1.23 | 4.50 | -12.63 | 3.32E-03 | 2.00 |
| Thecc1EG033222 | Retrotransposon protein,putative | 3.91 | -1.50 | 11.27 | 4.23E-03 | 2.00 |
| Thecc1EG007859 | Uncharacterized protein | 5.10 | -0.04 | 11.21 | 4.26E-03 | 1.99 |
| Thecc1EG042891 | F-box family protein,putative | -2.72 | 0.96 | -11.25 | 4.25E-03 | 1.98 |
| Thecc1EG027016 | Cysteine/Histidine-rich C1 domain family protein | 3.89 | -0.68 | 11.20 | 4.26E-03 | 1.97 |
| Thecc1EG014308 | Uncharacterized protein | 3.18 | -0.79 | 11.20 | 4.26E-03 | 1.97 |
| Thecc1EG027224 | Cyclin F-box | -4.64 | 1.13 | -11.28 | 4.23E-03 | 1.97 |
| Thecc1EG000461 | Coiled-coil domain-containing protein 109A,putative | 1.14 | 5.80 | 13.00 | 3.12E-03 | 1.97 |
| Thecc1EG041775 | NAD(P)-binding Rossmann-fold superfamily protein | 1.75 | 2.47 | 11.60 | 3.98E-03 | 1.96 |
| Thecc1EG021944 | Actin 3 | 0.86 | 6.92 | 12.95 | 3.15E-03 | 1.96 |
| Thecc1EG014606 | Leucine-rich repeat protein kinase family protein | 1.04 | 4.86 | 12.75 | 3.29E-03 | 1.95 |
| Thecc1EG043340 | Uncharacterized protein | -1.91 | 1.85 | -11.44 | 4.08E-03 | 1.95 |
| Thecc1EG043000 | Uncharacterized protein | 3.15 | 1.19 | 11.27 | 4.23E-03 | 1.94 |
| Thecc1EG031314 | Unknown | -3.82 | -0.91 | -11.12 | 4.34E-03 | 1.94 |
| Thecc1EG021733 | Uncharacterized protein | -2.89 | 0.13 | -11.09 | 4.36E-03 | 1.93 |
| Thecc1EG043044 | Ribosomal protein L10 family protein | 1.76 | 5.72 | 12.78 | 3.27E-03 | 1.93 |
| Thecc1EG016718 | 60S ribosomal protein L13 | 1.97 | 6.02 | 12.78 | 3.27E-03 | 1.93 |
| Thecc1EG041571 | Cyclin-dependent kinases regulatory subunit 1 | 0.89 | 6.50 | 12.89 | 3.20E-03 | 1.92 |
| Thecc1EG010619 | Cytochrome P450,family 71,subfamily B,polypeptide 36,putative | 2.69 | 4.30 | 12.20 | 3.55E-03 | 1.92 |
| Thecc1EG000992 | K-box region and MADS-box transcription factor family protein | -2.34 | 2.96 | -11.74 | 3.86E-03 | 1.90 |
| Thecc1EG029620 | Uncharacterized protein | 2.78 | -0.13 | 11.00 | 4.43E-03 | 1.90 |
| Thecc1EG021260 | Pectin lyase-like superfamily protein,putative | 3.73 | -1.32 | 11.01 | 4.42E-03 | 1.89 |
| Thecc1EG036876 | EamA-like transporter family | 2.57 | 1.69 | 11.24 | 4.26E-03 | 1.89 |
| Thecc1EG014292 | Uncharacterized protein | -1.26 | 4.73 | -12.40 | 3.43E-03 | 1.89 |
| Thecc1EG029865 | Lipoxygenase,putative | -2.62 | -0.11 | -10.99 | 4.43E-03 | 1.89 |
| Thecc1EG025366 | Rubredoxin family protein | 1.33 | 4.38 | 12.48 | 3.39E-03 | 1.89 |
| Thecc1EG001633 | Leucine-rich repeat transmembrane protein kinase | -1.23 | 5.33 | -12.71 | 3.30E-03 | 1.89 |
| Thecc1EG010769 | Remorin family protein | 1.11 | 5.09 | 12.63 | 3.32E-03 | 1.88 |
| Thecc1EG033193 | Unknown | -2.23 | 2.75 | -11.72 | 3.86E-03 | 1.88 |
| Thecc1EG025511 | Disease resistance family protein / LRR family protein,putative | 2.16 | 1.11 | 11.12 | 4.34E-03 | 1.88 |
| Thecc1EG031396 | Leucine-rich repeat (LRR) family protein | 4.94 | -2.03 | 11.00 | 4.43E-03 | 1.87 |
| Thecc1EG028721 | Late embryogenesis abundant hydroxyproline-rich glycofamily protein | 0.89 | 5.51 | 12.69 | 3.31E-03 | 1.87 |
| Thecc1EG046061 | Phosphoprotein phosphatase | -5.91 | -2.36 | -10.97 | 4.43E-03 | 1.87 |
| Thecc1EG007656 | Uncharacterized protein | -2.17 | 2.85 | -11.72 | 3.86E-03 | 1.86 |
| Thecc1EG034069 | Isoflavone reductase-like protein 4 | 1.20 | 6.55 | 12.70 | 3.30E-03 | 1.85 |
| Thecc1EG016805 | Family of Uncharacterized protein function,putative | -1.50 | 2.73 | -11.52 | 4.02E-03 | 1.85 |
| Thecc1EG017180 | Galactose oxidase/kelch repeat superfamily protein,putative | -1.96 | 2.74 | -11.53 | 4.02E-03 | 1.85 |
| Thecc1EG046931 | Myosin-related | -1.31 | 4.80 | -12.40 | 3.43E-03 | 1.84 |
| Thecc1EG045382 | Senescence-associated E3 ubiquitin ligase 1 | -1.00 | 5.15 | -12.64 | 3.32E-03 | 1.84 |
| Thecc1EG006394 | SU(VAR)3-9-like protein | 4.41 | -0.40 | 10.84 | 4.54E-03 | 1.84 |
| Thecc1EG020894 | Glutathione S-transferase tau 7 | 1.52 | 4.67 | 12.29 | 3.48E-03 | 1.84 |
| Thecc1EG004913 | S-adenosylmethionine synthetase 2 | 1.09 | 9.77 | 12.27 | 3.48E-03 | 1.83 |
| Thecc1EG032364 | Glutathione S-transferase TAU 19 | -1.04 | 8.15 | -12.51 | 3.39E-03 | 1.82 |
| Thecc1EG019497 | AWPM-19-like family protein,putative | -3.24 | 0.13 | -10.82 | 4.56E-03 | 1.82 |
| Thecc1EG006309 | UDP-Glycosyltransferase superfamily protein | -1.93 | 2.60 | -11.35 | 4.18E-03 | 1.82 |
| Thecc1EG021273 | TOPLESS-related 2 | -0.94 | 4.76 | -12.41 | 3.43E-03 | 1.81 |
| Thecc1EG031330 | Uncharacterized protein | 1.59 | 2.32 | 11.26 | 4.24E-03 | 1.80 |
| Thecc1EG028924 | Leucine-rich repeat transmembrane protein kinase protein,putative | -3.08 | -0.59 | -10.76 | 4.65E-03 | 1.80 |
| Thecc1EG015424 | Glycogen/starch synthases | -3.72 | 0.77 | -10.81 | 4.57E-03 | 1.79 |
| Thecc1EG004447 | ERD (early-responsive to dehydration stress) family protein | 1.55 | 2.83 | 11.33 | 4.19E-03 | 1.79 |
| Thecc1EG045134 | LRR and NB-ARC domains-containing disease resistance protein,putative | -1.71 | 2.28 | -11.21 | 4.26E-03 | 1.78 |
| Thecc1EG020791 | Zinc knuckle family protein,putative | 1.66 | 2.10 | 11.29 | 4.22E-03 | 1.78 |
| Thecc1EG033836 | Thioredoxin superfamily protein | 3.12 | -0.10 | 10.72 | 4.70E-03 | 1.77 |
| Thecc1EG018724 | F-box family protein | 0.92 | 5.60 | 12.50 | 3.39E-03 | 1.77 |
| Thecc1EG008435 | Uncharacterized protein | 0.95 | 7.02 | 12.48 | 3.39E-03 | 1.76 |
| Thecc1EG002069 | Leucine-rich repeat family protein | -0.97 | 4.68 | -12.20 | 3.55E-03 | 1.76 |
| Thecc1EG010916 | Uncharacterized protein | 1.27 | 3.64 | 11.78 | 3.84E-03 | 1.76 |
| Thecc1EG039440 | Uncharacterized protein | 7.14 | -1.62 | 10.65 | 4.80E-03 | 1.76 |
| Thecc1EG029775 | RAC-like 2 | -1.24 | 3.31 | -11.52 | 4.02E-03 | 1.75 |
| Thecc1EG031958 | Uncharacterized protein | 3.18 | 0.99 | 10.87 | 4.53E-03 | 1.75 |
| Thecc1EG020436 | RING/FYVE/PHD zinc finger superfamily protein | 1.06 | 5.29 | 12.36 | 3.46E-03 | 1.74 |
| Thecc1EG037947 | Indoleacetic acid-induced protein 16,putative | -1.54 | 3.09 | -11.35 | 4.18E-03 | 1.73 |
| Thecc1EG005592 | Unknown | -2.98 | 0.95 | -10.72 | 4.69E-03 | 1.73 |
| Thecc1EG030622 | Uncharacterized protein | -3.03 | 1.79 | -10.88 | 4.52E-03 | 1.73 |
| Thecc1EG037647 | CRS1 / YhbY domain-containing protein,putative | -0.88 | 5.37 | -12.38 | 3.44E-03 | 1.72 |
| Thecc1EG014819 | N-acetyl-l-glutamate kinase,putative | 4.22 | -1.32 | 10.59 | 4.85E-03 | 1.72 |
| Thecc1EG029211 | Dormancy/auxin associated family protein,putative | -2.98 | 3.27 | -11.48 | 4.06E-03 | 1.72 |
| Thecc1EG022526 | GDSL-like Lipase/Acylhydrolase superfamily protein,putative | 1.25 | 7.06 | 12.38 | 3.44E-03 | 1.72 |
| Thecc1EG021407 | Uncharacterized protein | 0.93 | 5.52 | 12.35 | 3.46E-03 | 1.71 |
| Thecc1EG034165 | Transducin/WD40 repeat-like superfamily protein | -1.25 | 3.56 | -11.57 | 3.99E-03 | 1.71 |
| Thecc1EG026630 | Methyltransferase,putative | -2.81 | -0.58 | -10.53 | 4.93E-03 | 1.70 |
| Thecc1EG026471 | NB-ARC domain-containing disease resistance protein,putative | -1.73 | 4.33 | -11.91 | 3.77E-03 | 1.69 |
| Thecc1EG015529 | Heavy metal transport/detoxification domain-containing protein | 2.18 | 4.55 | 11.86 | 3.80E-03 | 1.69 |
| Thecc1EG016419 | Uncharacterized protein | 1.81 | 3.42 | 11.55 | 4.01E-03 | 1.67 |
| Thecc1EG005587 | Uncharacterized protein | -1.08 | 3.97 | -11.70 | 3.87E-03 | 1.67 |
| Thecc1EG026527 | Kinase superfamily protein,putative | 2.86 | -0.92 | 10.41 | 5.12E-03 | 1.65 |
| Thecc1EG020010 | Chlorsulfuron/imidazolinone resistant 1 | -1.18 | 9.33 | -11.94 | 3.75E-03 | 1.65 |
| Thecc1EG004368 | Uncharacterized protein | 4.64 | 0.62 | 10.51 | 4.96E-03 | 1.64 |
| Thecc1EG014626 | Cytochrome P450 94A2 | 3.10 | -0.45 | 10.40 | 5.13E-03 | 1.64 |
| Thecc1EG005113 | Pyridoxamine 5'-phosphate oxidase family protein | 0.92 | 4.83 | 12.03 | 3.68E-03 | 1.63 |
| Thecc1EG024082 | PQ-loop repeat family protein / transmembrane family protein,putative | 4.99 | -2.16 | 10.35 | 5.18E-03 | 1.62 |
| Thecc1EG045533 | Appr-1-p processing enzyme family protein | 1.35 | 4.23 | 11.61 | 3.97E-03 | 1.62 |
| Thecc1EG015705 | AIG2-like (avirulence induced gene) family protein,putative | 1.32 | 2.99 | 11.10 | 4.35E-03 | 1.62 |
| Thecc1EG016016 | Flavin-binding monooxygenase family protein | -1.15 | 5.47 | -12.16 | 3.56E-03 | 1.62 |
| Thecc1EG004630 | Alpha-L RNA-binding motif/Ribosomal protein S4 family protein | 4.22 | -0.61 | 10.32 | 5.19E-03 | 1.61 |
| Thecc1EG026957 | Cc-nbs-lrr resistance protein,putative | 2.63 | -0.20 | 10.33 | 5.19E-03 | 1.61 |
| Thecc1EG032245 | Cytochrome P450,family 72,subfamily A,polypeptide 15,putative | 1.12 | 5.79 | 12.16 | 3.56E-03 | 1.61 |
| Thecc1EG040687 | Aldehyde dehydrogenase family 3 member F1 | -4.81 | 0.44 | -10.35 | 5.18E-03 | 1.60 |
| Thecc1EG025555 | Zinc-binding alcohol dehydrogenase family protein,putative | -1.30 | 6.08 | -12.15 | 3.56E-03 | 1.60 |
| Thecc1EG010489 | Copper amine oxidase family protein | -1.73 | 5.25 | -11.98 | 3.74E-03 | 1.59 |
| Thecc1EG004665 | Magnesium/proton exchanger | -0.98 | 4.64 | -11.97 | 3.74E-03 | 1.59 |
| Thecc1EG012428 | Uncharacterized protein | 1.66 | 1.79 | 10.84 | 4.54E-03 | 1.59 |
| Thecc1EG010637 | Cellulase protein | 1.74 | 2.79 | 10.99 | 4.43E-03 | 1.58 |
| Thecc1EG021357 | Aldehyde dehydrogenase 22A1 | -0.76 | 5.89 | -12.12 | 3.59E-03 | 1.58 |
| Thecc1EG015731 | Uncharacterized protein | -2.22 | 0.58 | -10.34 | 5.18E-03 | 1.58 |
| Thecc1EG042105 | Uncharacterized protein | 4.16 | -1.08 | 10.25 | 5.28E-03 | 1.58 |
| Thecc1EG020951 | Unknown | -4.84 | -2.11 | -10.23 | 5.30E-03 | 1.57 |
| Thecc1EG015904 | Uncharacterized protein | 1.17 | 6.37 | 12.07 | 3.64E-03 | 1.56 |
| Thecc1EG031639 | CC-NBS-LRR class disease resistance protein,putative | -4.57 | -0.33 | -10.18 | 5.38E-03 | 1.55 |
| Thecc1EG017016 | S-adenosyl-L-methionine-dependent methyltransferases superfamily protein | 2.24 | 0.23 | 10.31 | 5.20E-03 | 1.55 |
| Thecc1EG002268 | Uncharacterized protein | -5.88 | -1.19 | -10.18 | 5.38E-03 | 1.55 |
| Thecc1EG021701 | Vacuolar cation/proton exchanger 3 | -2.54 | 3.09 | -11.02 | 4.41E-03 | 1.55 |
| Thecc1EG030425 | Heavy metal transport/detoxification superfamily protein,putative | -0.89 | 8.62 | -11.81 | 3.82E-03 | 1.55 |
| Thecc1EG001916 | NAD(P)-linked oxidoreductase superfamily protein | 1.51 | 3.18 | 11.32 | 4.19E-03 | 1.54 |
| Thecc1EG004207 | Alpha/beta-Hydrolases superfamily protein | -1.14 | 7.46 | -11.94 | 3.75E-03 | 1.54 |
| Thecc1EG021919 | Unknown | -2.61 | -0.42 | -10.17 | 5.38E-03 | 1.54 |
| Thecc1EG025437 | UDP-glucosyl transferase 78D2,putative | -2.89 | 2.70 | -10.87 | 4.53E-03 | 1.53 |
| Thecc1EG026407 | Sec23/Sec24 protein transport family protein | -1.91 | 3.22 | -10.99 | 4.43E-03 | 1.53 |
| Thecc1EG010355 | Quinone oxidoreductase-like protein | 1.20 | 3.52 | 11.10 | 4.35E-03 | 1.52 |
| Thecc1EG024550 | Esterase d,s-formylglutathione hydrolase | 0.82 | 5.75 | 11.98 | 3.74E-03 | 1.52 |
| Thecc1EG040278 | Uncharacterized protein | 1.30 | 3.35 | 11.10 | 4.35E-03 | 1.51 |
| Thecc1EG030113 | Cysteine-rich protein,putative | -3.21 | 0.53 | -10.25 | 5.28E-03 | 1.50 |
| Thecc1EG011949 | Metal ion binding protein,putative | -2.41 | 1.83 | -10.49 | 5.01E-03 | 1.50 |
| Thecc1EG020318 | NBS type disease resistance protein,putative | -4.46 | -1.26 | -10.06 | 5.54E-03 | 1.50 |
| Thecc1EG045119 | Amidohydrolase family,ISF2 | -1.48 | 3.56 | -11.09 | 4.35E-03 | 1.50 |
| Thecc1EG005065 | Rho GTPase activating protein with PAK-box/P21-Rho-binding domain | -1.99 | 1.74 | -10.37 | 5.17E-03 | 1.49 |
| Thecc1EG026501 | Cc-nbs-lrr resistance-like protein | 1.73 | 1.87 | 10.43 | 5.11E-03 | 1.49 |
| Thecc1EG026900 | SIT4 phosphatase-associated family protein | 0.84 | 6.51 | 11.90 | 3.78E-03 | 1.49 |
| Thecc1EG016230 | Leucine-rich repeat protein kinase family protein | 1.63 | 5.72 | 11.78 | 3.84E-03 | 1.48 |
| Thecc1EG007739 | Glycosyl hydrolase 9C1 | -2.05 | 0.64 | -10.21 | 5.32E-03 | 1.48 |
| Thecc1EG006157 | Uncharacterized protein | 1.23 | 3.39 | 11.03 | 4.41E-03 | 1.47 |
| Thecc1EG019728 | Kinase,putative | -1.19 | 5.48 | -11.83 | 3.80E-03 | 1.47 |
| Thecc1EG025135 | Glutamine-fructose-6-phosphate transaminase (isomerizing)s,sugar binding,transaminases | -0.91 | 5.35 | -11.80 | 3.82E-03 | 1.47 |
| Thecc1EG008802 | DNAse I-like superfamily protein | -2.84 | -0.62 | -9.98 | 5.61E-03 | 1.46 |
| Thecc1EG004378 | Uncharacterized protein | -5.75 | -1.67 | -9.98 | 5.61E-03 | 1.46 |
| Thecc1EG031520 | Proton pump interactor 1 | -0.86 | 5.98 | -11.83 | 3.80E-03 | 1.45 |
| Thecc1EG021411 | Cell wall / vacuolar inhibitor of fructosidase 1,putative | -2.43 | -0.42 | -9.96 | 5.62E-03 | 1.44 |
| Thecc1EG043895 | Pathogenesis-related protein P2 | 1.63 | 3.98 | 11.22 | 4.26E-03 | 1.43 |
| Thecc1EG022181 | Chromatin remodeling complex subunit-like protein | -1.27 | 5.81 | -11.77 | 3.84E-03 | 1.43 |
| Thecc1EG007908 | Uncharacterized protein | -4.63 | 1.23 | -10.08 | 5.51E-03 | 1.42 |
| Thecc1EG010361 | Uncharacterized protein | 4.41 | -1.42 | 9.87 | 5.72E-03 | 1.41 |
| Thecc1EG034610 | Amino acid permease 6 | -0.80 | 5.92 | -11.70 | 3.87E-03 | 1.40 |
| Thecc1EG032490 | Uncharacterized protein | -0.82 | 5.54 | -11.70 | 3.87E-03 | 1.40 |
| Thecc1EG042908 | Ankyrin repeat-containing protein,putative | -6.04 | 0.21 | -9.88 | 5.72E-03 | 1.40 |
| Thecc1EG041342 | Photosystem I subunit O | 2.13 | 4.27 | 11.16 | 4.31E-03 | 1.40 |
| Thecc1EG036193 | Uncharacterized protein | -1.24 | 3.81 | -10.96 | 4.43E-03 | 1.40 |
| Thecc1EG046832 | P-loop containing nucleoside triphosphate hydrolases superfamily protein,putative | -3.94 | 0.86 | -9.99 | 5.61E-03 | 1.39 |
| Thecc1EG014924 | Mitochondrion-localized small heat shock protein 23.6,putative | 0.89 | 5.04 | 11.57 | 3.99E-03 | 1.39 |
| Thecc1EG021987 | RNA-binding family protein | 1.36 | 3.76 | 10.92 | 4.48E-03 | 1.39 |
| Thecc1EG034346 | OPC-8:0 CoA ligase1 | -2.87 | 0.81 | -10.00 | 5.60E-03 | 1.38 |
| Thecc1EG011999 | Ubiquitin-protein ligase,putative-like protein | 2.66 | 0.25 | 9.84 | 5.78E-03 | 1.38 |
| Thecc1EG004933 | F20D23.27 protein,putative | 1.81 | 3.95 | 11.04 | 4.41E-03 | 1.38 |
| Thecc1EG033938 | Uncharacterized protein | 0.84 | 5.62 | 11.64 | 3.94E-03 | 1.37 |
| Thecc1EG020024 | Actin-like ATPase superfamily protein | -0.79 | 5.82 | -11.66 | 3.93E-03 | 1.37 |
| Thecc1EG020076 | Cell division control protein 48 C | -1.31 | 5.03 | -11.44 | 4.08E-03 | 1.36 |
| Thecc1EG020175 | DNA topoisomerase,type IA,core | 0.87 | 5.17 | 11.53 | 4.02E-03 | 1.36 |
| Thecc1EG005905 | Zinc finger A20 and AN1 domain-containing stress-associated protein 4,putative | -0.91 | 9.00 | -11.38 | 4.17E-03 | 1.36 |
| Thecc1EG030095 | Cysteine-rich RLK (RECEPTOR-like protein kinase) 29 | 4.27 | -1.02 | 9.78 | 5.88E-03 | 1.36 |
| Thecc1EG021808 | UDP-Glycosyltransferase superfamily protein | 3.68 | 3.81 | 10.83 | 4.54E-03 | 1.36 |
| Thecc1EG021035 | AWPM-19-like family protein | -1.17 | 3.03 | -10.75 | 4.66E-03 | 1.36 |
| Thecc1EG037912 | GNOM-like 2 | 1.59 | 2.51 | 10.40 | 5.13E-03 | 1.35 |
| Thecc1EG033771 | Unknown | -1.71 | 1.94 | -10.12 | 5.48E-03 | 1.34 |
| Thecc1EG037756 | Profilin 5 | 2.27 | 0.92 | 9.95 | 5.65E-03 | 1.34 |
| Thecc1EG006101 | NB-ARC domain-containing disease resistance protein | -0.78 | 5.64 | -11.56 | 3.99E-03 | 1.33 |
| Thecc1EG015774 | S-norcoclaurine synthase 1,putative | 1.28 | 4.42 | 11.28 | 4.23E-03 | 1.32 |
| Thecc1EG018810 | Uncharacterized protein | -1.10 | 5.69 | -11.48 | 4.06E-03 | 1.32 |
| Thecc1EG006136 | 12-oxophytodienoate reductase 1 | 2.40 | 0.41 | 9.78 | 5.88E-03 | 1.31 |
| Thecc1EG015982 | Small heat shock protein,putative | 0.82 | 5.30 | 11.49 | 4.05E-03 | 1.31 |
| Thecc1EG020574 | SEY1 | 2.11 | 3.96 | 10.92 | 4.47E-03 | 1.31 |
| Thecc1EG041771 | NAD(P)-binding Rossmann-fold superfamily protein | -1.99 | 1.13 | -9.97 | 5.61E-03 | 1.31 |
| Thecc1EG024664 | Uncharacterized protein | -6.41 | -1.08 | -9.59 | 6.18E-03 | 1.28 |
| Thecc1EG016334 | Monodehydroascorbate reductase 6 | -0.75 | 5.98 | -11.46 | 4.07E-03 | 1.27 |
| Thecc1EG037616 | NB-ARC domain-containing disease resistance protein,putative | -1.83 | 2.15 | -10.10 | 5.50E-03 | 1.26 |
| Thecc1EG011460 | Cytochrome P450 | -5.34 | -1.40 | -9.56 | 6.21E-03 | 1.26 |
| Thecc1EG027108 | BCL-2-associated athanogene 1,putative | 1.84 | 5.56 | 11.24 | 4.26E-03 | 1.26 |
| Thecc1EG016882 | GDSL-like Lipase/Acylhydrolase superfamily protein | -0.85 | 5.90 | -11.42 | 4.10E-03 | 1.26 |
| Thecc1EG046775 | 2-oxoglutarate (2OG) and Fe(II)-dependent oxygenase superfamily protein,putative | 2.96 | 2.00 | 10.05 | 5.56E-03 | 1.25 |
| Thecc1EG027060 | FAD-binding Berberine family protein | -1.60 | 2.00 | -10.02 | 5.58E-03 | 1.25 |
| Thecc1EG021961 | Heat shock cognate protein 70-1 | -0.95 | 11.17 | -10.91 | 4.49E-03 | 1.25 |
| Thecc1EG036126 | Leucine-rich receptor protein kinase family protein | 5.61 | -2.19 | 9.52 | 6.27E-03 | 1.25 |
| Thecc1EG030921 | Uncharacterized protein | -1.23 | 3.48 | -10.63 | 4.82E-03 | 1.24 |
| Thecc1EG028418 | Dehydroquinate dehydratase / shikimate dehydrogenas,putative | -1.51 | 1.80 | -9.97 | 5.61E-03 | 1.23 |
| Thecc1EG015906 | Nudix hydrolase | 0.97 | 4.86 | 11.19 | 4.27E-03 | 1.23 |
| Thecc1EG027002 | Cysteine/Histidine-rich C1 domain family protein | 0.98 | 6.79 | 11.33 | 4.19E-03 | 1.22 |
| Thecc1EG020095 | Cysteine/Histidine-rich C1 domain family protein,putative | -2.49 | 1.85 | -9.94 | 5.66E-03 | 1.22 |
| Thecc1EG020068 | Uncharacterized protein | 1.41 | 2.21 | 10.04 | 5.57E-03 | 1.22 |
| Thecc1EG014583 | Glyceraldehyde-3-phosphate dehydrogenase C subunit 1 | -1.61 | 4.68 | -10.97 | 4.43E-03 | 1.21 |
| Thecc1EG040775 | Uncharacterized protein | 1.41 | 2.86 | 10.28 | 5.25E-03 | 1.21 |
| Thecc1EG020285 | Gb:AAC32909.1,putative | -0.79 | 5.25 | -11.21 | 4.26E-03 | 1.21 |
| Thecc1EG007477 | UDP-glycosyltransferase 85A1 | 1.51 | 3.09 | 10.71 | 4.70E-03 | 1.21 |
| Thecc1EG034851 | Mitochondrial ribosomal protein L27 | -0.79 | 6.14 | -11.32 | 4.19E-03 | 1.20 |
| Thecc1EG016693 | SWIB/MDM2 domain superfamily protein | 2.33 | 2.31 | 9.95 | 5.65E-03 | 1.20 |
| Thecc1EG016543 | NAD(P)-binding Rossmann-fold superfamily protein | 3.48 | 0.65 | 9.59 | 6.18E-03 | 1.20 |
| Thecc1EG011492 | Major facilitator superfamily protein | 4.06 | 3.68 | 10.34 | 5.18E-03 | 1.19 |
| Thecc1EG005391 | NAD(P)-binding Rossmann-fold superfamily protein,putative | 4.05 | 4.08 | 10.51 | 4.97E-03 | 1.18 |
| Thecc1EG024477 | Unknown | 1.22 | 4.23 | 10.94 | 4.46E-03 | 1.18 |
| Thecc1EG028870 | Avr9/Cf-9 rapidly elicited protein 146,putative | -0.89 | 4.29 | -10.96 | 4.43E-03 | 1.18 |
| Thecc1EG043901 | Catalytic,putative | 1.58 | 2.96 | 10.26 | 5.27E-03 | 1.17 |
| Thecc1EG037611 | UDP-Glycosyltransferase superfamily protein | -0.77 | 6.80 | -11.22 | 4.26E-03 | 1.17 |
| Thecc1EG012291 | PLAC8 family protein | -2.13 | 1.25 | -9.69 | 6.02E-03 | 1.17 |
| Thecc1EG014418 | NB-ARC domain-containing disease resistance protein,putative | 1.14 | 3.90 | 10.58 | 4.85E-03 | 1.15 |
| Thecc1EG046803 | NAD(P)-binding Rossmann-fold superfamily protein | 0.98 | 5.94 | 11.20 | 4.26E-03 | 1.15 |
| Thecc1EG014931 | Glycosyl hydrolases family 32 protein | 1.50 | 2.72 | 10.32 | 5.19E-03 | 1.15 |
| Thecc1EG043180 | Adenosine kinase 2 | 1.20 | 5.98 | 11.18 | 4.28E-03 | 1.15 |
| Thecc1EG021408 | Plant invertase/pectin methylesterase inhibitor superfamily protein,putative | 0.95 | 4.63 | 11.05 | 4.41E-03 | 1.14 |
| Thecc1EG029586 | Nodulin MtN3 family protein,putative | 1.62 | 1.56 | 9.88 | 5.72E-03 | 1.13 |
| Thecc1EG007024 | RNA-binding protein 47A | -0.75 | 7.03 | -11.14 | 4.33E-03 | 1.13 |
| Thecc1EG021326 | Chitinase-like protein 2 | -1.22 | 2.94 | -10.08 | 5.51E-03 | 1.13 |
| Thecc1EG047072 | Serine carboxypeptidase-like 7,putative | 1.89 | 3.31 | 10.34 | 5.18E-03 | 1.12 |
| Thecc1EG016671 | Mechanosensitive channel of small conductance-like 10 | -1.61 | 1.84 | -9.83 | 5.79E-03 | 1.12 |
| Thecc1EG016858 | Aldehyde dehydrogenase 2B4 | 0.97 | 6.91 | 11.12 | 4.34E-03 | 1.12 |
| Thecc1EG005514 | Secretory 1A | 3.45 | -0.73 | 9.25 | 6.67E-03 | 1.12 |
| Thecc1EG043006 | Ankyrin repeat-containing,putative-like protein | 2.82 | 0.09 | 9.32 | 6.60E-03 | 1.12 |
| Thecc1EG043102 | Serine carboxypeptidase-like 27 | 0.80 | 5.57 | 11.03 | 4.41E-03 | 1.11 |
| Thecc1EG046726 | Cytochrome P450 | -0.86 | 6.08 | -11.14 | 4.33E-03 | 1.11 |
| Thecc1EG036692 | Sucrose-proton symporter 2 | -1.20 | 4.90 | -10.83 | 4.55E-03 | 1.11 |
| Thecc1EG021806 | UDP-glucosyl transferase 85A3,putative | -0.80 | 5.15 | -10.96 | 4.43E-03 | 1.10 |
| Thecc1EG022244 | ATP binding cassette subfamily B1 | 0.81 | 8.18 | 10.97 | 4.43E-03 | 1.10 |
| Thecc1EG011599 | UDP-glucosyl transferase 88A1,putative | 1.14 | 4.04 | 10.76 | 4.65E-03 | 1.10 |
| Thecc1EG025729 | Tubulin folding cofactor A (KIESEL) | 0.78 | 6.72 | 11.05 | 4.41E-03 | 1.08 |
| Thecc1EG020017 | PfkB-like carbohydrate kinase family protein | 0.77 | 6.84 | 11.02 | 4.41E-03 | 1.08 |
| Thecc1EG022022 | Ankyrin repeat family protein | 2.43 | -0.23 | 9.19 | 6.79E-03 | 1.07 |
| Thecc1EG016739 | Uncharacterized protein | -0.78 | 4.92 | -10.85 | 4.54E-03 | 1.07 |
| Thecc1EG027098 | Glucose-methanol-choline (GMC) oxidoreductase family protein | 1.68 | 1.71 | 9.61 | 6.16E-03 | 1.07 |
| Thecc1EG019366 | Uncharacterized protein | 1.67 | 1.67 | 9.72 | 5.98E-03 | 1.06 |
| Thecc1EG029233 | Subtilase family protein | 0.87 | 6.80 | 11.01 | 4.43E-03 | 1.06 |
| Thecc1EG034625 | Glycosyl hydrolase family 10 protein / carbohydrate-binding domain-containing protein | -0.88 | 4.53 | -10.67 | 4.77E-03 | 1.06 |
| Thecc1EG028725 | Late embryogenesis abundant hydroxyproline-rich glycofamily protein | 2.84 | -0.30 | 9.19 | 6.79E-03 | 1.05 |
| Thecc1EG029274 | RING/U-box superfamily protein,putative | -1.84 | 2.22 | -9.65 | 6.09E-03 | 1.05 |
| Thecc1EG043271 | Armadillo/beta-catenin repeat family protein / kinesin motor family protein | -0.94 | 6.45 | -10.98 | 4.43E-03 | 1.04 |
| Thecc1EG047018 | Oxidoreductase family protein | 1.82 | 2.80 | 9.97 | 5.61E-03 | 1.04 |
| Thecc1EG024708 | Uncharacterized protein | 0.84 | 7.33 | 10.89 | 4.51E-03 | 1.03 |
| Thecc1EG004215 | Primosome PriB/single-strand DNA-binding,putative | -0.76 | 5.18 | -10.87 | 4.53E-03 | 1.03 |
| Thecc1EG031271 | Uncharacterized protein | 1.50 | 2.53 | 9.80 | 5.85E-03 | 1.02 |
| Thecc1EG031840 | Uncharacterized protein | -3.79 | -1.22 | -9.05 | 7.00E-03 | 1.02 |
| Thecc1EG010648 | Transducin/WD40 repeat-like superfamily protein | 2.15 | 0.94 | 9.27 | 6.66E-03 | 1.01 |
| Thecc1EG030756 | Na+/H+ (sodium hydrogen) exchanger 3 | -1.90 | 2.99 | -9.89 | 5.72E-03 | 1.00 |
| Thecc1EG007919 | HXXXD-type acyl-transferase family protein,putative | -2.16 | 0.26 | -9.16 | 6.85E-03 | 1.00 |
| Thecc1EG010872 | Serine carboxypeptidase-like 50 | -0.86 | 6.61 | -10.86 | 4.53E-03 | 0.99 |
| Thecc1EG046921 | Mitochondrial-processing peptidase subunit beta,mitochondrial,putative | -0.70 | 5.77 | -10.84 | 4.54E-03 | 0.99 |
| Thecc1EG025243 | Uncharacterized protein | 1.58 | 2.28 | 9.65 | 6.09E-03 | 0.99 |
| Thecc1EG037843 | Transducin/WD40 repeat-like superfamily protein | -1.00 | 3.80 | -10.11 | 5.50E-03 | 0.98 |
| Thecc1EG031310 | Uncharacterized protein | -1.10 | 4.02 | -10.34 | 5.18E-03 | 0.98 |
| Thecc1EG037334 | Aminopeptidase M1 | -2.67 | -0.63 | -8.97 | 7.13E-03 | 0.96 |
| Thecc1EG026977 | Coatomer epsilon subunit | -1.53 | 3.30 | -10.02 | 5.58E-03 | 0.96 |
| Thecc1EG031319 | Uncharacterized protein | -1.12 | 5.44 | -10.74 | 4.67E-03 | 0.94 |
| Thecc1EG017093 | Uncharacterized protein | -3.33 | 0.93 | -9.13 | 6.90E-03 | 0.94 |
| Thecc1EG016075 | Glycine cleavage T-protein family | 1.34 | 3.24 | 9.93 | 5.66E-03 | 0.94 |
| Thecc1EG011145 | Indeterminate(ID)-domain 5,putative | -0.79 | 6.85 | -10.75 | 4.66E-03 | 0.93 |
| Thecc1EG017094 | Uncharacterized protein | -2.48 | -0.38 | -8.91 | 7.25E-03 | 0.91 |
| Thecc1EG012217 | 2-oxoglutarate (2OG) and Fe(II)-dependent oxygenase superfamily protein | 0.77 | 4.86 | 10.58 | 4.85E-03 | 0.91 |
| Thecc1EG041612 | Uncharacterized protein | -1.57 | 1.44 | -9.28 | 6.66E-03 | 0.91 |
| Thecc1EG046036 | Histidine kinase | -2.26 | 0.78 | -9.01 | 7.08E-03 | 0.90 |
| Thecc1EG046811 | Major facilitator superfamily protein,putative | 1.41 | 5.25 | 10.62 | 4.82E-03 | 0.90 |
| Thecc1EG024770 | Cysteine/Histidine-rich C1 domain family protein | 1.69 | 2.38 | 9.50 | 6.28E-03 | 0.90 |
| Thecc1EG021812 | Uncharacterized protein | 1.98 | 3.34 | 10.00 | 5.60E-03 | 0.89 |
| Thecc1EG004592 | Glutathione S-transferase 7,putative | 0.71 | 7.33 | 10.64 | 4.80E-03 | 0.89 |
| Thecc1EG011045 | Ankyrin repeat-containing protein | 1.33 | 4.15 | 10.22 | 5.32E-03 | 0.89 |
| Thecc1EG034041 | Chloroplast thylakoid membrane | 0.88 | 4.54 | 10.32 | 5.19E-03 | 0.89 |
| Thecc1EG018162 | ABC transporter family protein | -1.01 | 4.13 | -10.23 | 5.30E-03 | 0.89 |
| Thecc1EG046206 | 3R-linalool synthase | 2.28 | 2.74 | 9.62 | 6.14E-03 | 0.89 |
| Thecc1EG041335 | Uncharacterized protein | 0.93 | 7.96 | 10.54 | 4.92E-03 | 0.89 |
| Thecc1EG015015 | DEAD-box ATP-dependent RNA helicase 7 | -0.81 | 9.72 | -10.33 | 5.18E-03 | 0.88 |
| Thecc1EG021193 | RNA binding | 0.77 | 5.22 | 10.59 | 4.85E-03 | 0.88 |
| Thecc1EG041913 | DHBP synthase RibB-like alpha/beta domain,GTP cyclohydrolase II | 1.76 | 0.93 | 9.14 | 6.88E-03 | 0.87 |
| Thecc1EG023063 | MD-2-related lipid recognition domain-containing protein / ML domain-containing protein | 0.79 | 5.87 | 10.66 | 4.80E-03 | 0.87 |
| Thecc1EG020902 | XB3 ortholog 5 in,putative | -0.69 | 6.82 | -10.62 | 4.82E-03 | 0.86 |
| Thecc1EG021994 | Ras-related small GTP-binding family protein | -0.87 | 7.51 | -10.53 | 4.93E-03 | 0.86 |
| Thecc1EG036888 | Major facilitator superfamily protein | -2.67 | 0.48 | -8.97 | 7.13E-03 | 0.86 |
| Thecc1EG022603 | Thioredoxin family protein | -1.23 | 6.15 | -10.62 | 4.82E-03 | 0.86 |
| Thecc1EG020479 | Glutamine synthetase 2 | 1.28 | 6.61 | 10.59 | 4.85E-03 | 0.85 |
| Thecc1EG016789 | Uncharacterized protein | -2.04 | 0.02 | -8.84 | 7.35E-03 | 0.85 |
| Thecc1EG013603 | Tetratricopeptide repeat-containing protein | 1.84 | 2.53 | 9.43 | 6.40E-03 | 0.85 |
| Thecc1EG027247 | Ubiquitin system component Cue protein,putative | -1.38 | 6.84 | -10.58 | 4.85E-03 | 0.85 |
| Thecc1EG021252 | Acyl-CoA dehydrogenase-related | -0.74 | 6.12 | -10.62 | 4.82E-03 | 0.85 |
| Thecc1EG016037 | Beta vacuolar processing enzyme | -2.57 | 1.90 | -9.07 | 6.98E-03 | 0.84 |
| Thecc1EG046106 | Ankyrin repeat family protein,putative | 1.29 | 3.26 | 9.57 | 6.21E-03 | 0.83 |
| Thecc1EG020979 | Cyclopropane fatty acid synthase | -2.75 | -0.72 | -8.73 | 7.58E-03 | 0.83 |
| Thecc1EG044430 | Sucrose-6F-phosphate phosphohydrolase family protein | -1.36 | 3.70 | -9.91 | 5.70E-03 | 0.83 |
| Thecc1EG006035 | Uncharacterized protein | 1.34 | 5.27 | 10.46 | 5.06E-03 | 0.83 |
| Thecc1EG033971 | Ribosomal protein S9 | 0.83 | 5.05 | 10.39 | 5.15E-03 | 0.82 |
| Thecc1EG021107 | Deoxyxylulose-5-phosphate synthase | 1.13 | 7.89 | 10.44 | 5.09E-03 | 0.82 |
| Thecc1EG015560 | WAS/WASL-interacting protein family member 1 | 1.27 | 2.76 | 9.46 | 6.37E-03 | 0.82 |
| Thecc1EG030866 | Aconitase 3 | -2.69 | 0.73 | -8.90 | 7.25E-03 | 0.82 |
| Thecc1EG033112 | Uncharacterized protein | -1.60 | 0.77 | -9.10 | 6.94E-03 | 0.81 |
| Thecc1EG020572 | Serine-threonine protein kinase,plant-type,putative | -0.82 | 4.84 | -10.23 | 5.30E-03 | 0.80 |
| Thecc1EG029017 | PRA1 family protein | 1.09 | 3.64 | 9.76 | 5.91E-03 | 0.80 |
| Thecc1EG027424 | NRAMP metal ion transporter 6 | -1.68 | 2.49 | -9.40 | 6.45E-03 | 0.80 |
| Thecc1EG016859 | Serine-threonine protein kinase,putative | -2.15 | -0.26 | -8.74 | 7.58E-03 | 0.79 |
| Thecc1EG031336 | Unknown | 5.42 | 0.48 | 8.77 | 7.53E-03 | 0.79 |
| Thecc1EG029891 | DNA mismatch repair protein MutS | -1.13 | 3.26 | -9.69 | 6.02E-03 | 0.78 |
| Thecc1EG035170 | ATPase family associated with various cellular activities (AAA) | -1.11 | 2.99 | -9.49 | 6.30E-03 | 0.78 |
| Thecc1EG032675 | Uncharacterized protein | -1.73 | 1.04 | -9.02 | 7.05E-03 | 0.78 |
| Thecc1EG005805 | NB-ARC domain-containing disease resistance-like protein | -1.98 | 1.01 | -8.86 | 7.31E-03 | 0.77 |
| Thecc1EG031530 | Nbs-lrr resistance protein | -2.95 | -0.51 | -8.60 | 7.90E-03 | 0.77 |
| Thecc1EG003900 | Photosystem II reaction center PSB28 protein | 0.95 | 3.81 | 9.93 | 5.66E-03 | 0.76 |
| Thecc1EG006238 | Uncharacterized protein | -0.69 | 5.77 | -10.42 | 5.12E-03 | 0.75 |
| Thecc1EG015571 | Nodulin MtN21 /EamA-like transporter family protein | -1.45 | 1.46 | -9.03 | 7.03E-03 | 0.75 |
| Thecc1EG011610 | Uncharacterized protein | 0.78 | 6.38 | 10.42 | 5.12E-03 | 0.75 |
| Thecc1EG015490 | SAUR family protein | 1.05 | 4.99 | 10.26 | 5.27E-03 | 0.75 |
| Thecc1EG030905 | GDSL-like Lipase/Acylhydrolase superfamily protein,putative | 0.73 | 6.27 | 10.41 | 5.12E-03 | 0.74 |
| Thecc1EG020910 | Uncharacterized protein | 1.13 | 2.82 | 9.38 | 6.49E-03 | 0.74 |
| Thecc1EG005200 | Arabinogalactan protein 20 | 1.10 | 7.32 | 10.35 | 5.18E-03 | 0.74 |
| Thecc1EG022568 | Catalytic,putative | -1.62 | 3.55 | -9.57 | 6.21E-03 | 0.74 |
| Thecc1EG015662 | Uncharacterized protein | -0.79 | 5.17 | -10.30 | 5.21E-03 | 0.74 |
| Thecc1EG022536 | Solute carrier family 35 member F2 | 1.69 | 4.36 | 9.98 | 5.61E-03 | 0.73 |
| Thecc1EG017443 | Monooxygenase,putative | 2.40 | 0.66 | 8.74 | 7.58E-03 | 0.73 |
| Thecc1EG003170 | Eukaryotic release factor 1-3 | -0.69 | 7.57 | -10.28 | 5.25E-03 | 0.73 |
| Thecc1EG014649 | SecE/sec61-gamma protein transport protein | 0.66 | 6.36 | 10.37 | 5.17E-03 | 0.72 |
| Thecc1EG038283 | Unknown | -2.01 | 1.58 | -8.82 | 7.37E-03 | 0.72 |
| Thecc1EG008253 | Unknown | -1.27 | 2.92 | -9.48 | 6.33E-03 | 0.72 |
| Thecc1EG011591 | Nodulin-related protein 1,putative | -1.80 | 0.88 | -8.73 | 7.58E-03 | 0.72 |
| Thecc1EG037833 | Uncharacterized protein | 1.69 | 1.16 | 8.77 | 7.53E-03 | 0.72 |
| Thecc1EG005821 | Serine carboxypeptidase-like 40 | -1.95 | 1.30 | -8.87 | 7.31E-03 | 0.72 |
| Thecc1EG042222 | Uncharacterized protein | 0.82 | 4.10 | 9.92 | 5.69E-03 | 0.71 |
| Thecc1EG031125 | Histone H2A 10 | 0.76 | 7.63 | 10.25 | 5.28E-03 | 0.71 |
| Thecc1EG020050 | Unknown | -2.37 | -0.49 | -8.53 | 8.08E-03 | 0.71 |
| Thecc1EG025894 | C2 calcium/lipid-binding plant phosphoribosyltransferase family protein | -1.20 | 2.87 | -9.35 | 6.54E-03 | 0.71 |
| Thecc1EG000728 | RING finger protein B,putative | 1.01 | 3.08 | 9.52 | 6.27E-03 | 0.70 |
| Thecc1EG037044 | Leucine-rich repeat receptor-like protein kinase family protein | -1.64 | 3.76 | -9.76 | 5.90E-03 | 0.70 |
| Thecc1EG020311 | Endoplasmic reticulum retention defective 2B | 0.95 | 4.81 | 10.13 | 5.47E-03 | 0.70 |
| Thecc1EG015749 | NAC domain protein,IPR003441 | -1.27 | 2.09 | -9.09 | 6.95E-03 | 0.70 |
| Thecc1EG019821 | Uncharacterized protein | -2.62 | 3.63 | -9.58 | 6.18E-03 | 0.69 |
| Thecc1EG032049 | Uncharacterized protein | -1.33 | 2.36 | -9.15 | 6.86E-03 | 0.68 |
| Thecc1EG037073 | Uncharacterized protein | 2.82 | 0.48 | 8.69 | 7.67E-03 | 0.68 |
| Thecc1EG041166 | Structural maintenance of chromosomes (SMC) family protein | -0.92 | 4.42 | -9.88 | 5.72E-03 | 0.68 |
| Thecc1EG031898 | Flavonol 4'-sulfotransferase | -3.90 | 0.16 | -8.46 | 8.23E-03 | 0.68 |
| Thecc1EG029931 | NAC domain containing protein 25 | 1.97 | 1.33 | 8.80 | 7.44E-03 | 0.67 |
| Thecc1EG027287 | Copper binding protein 6,putative | 1.36 | 2.18 | 9.07 | 6.98E-03 | 0.67 |
| Thecc1EG006367 | Palmitoyltransferase TIP1,putative | -1.90 | 0.15 | -8.55 | 8.04E-03 | 0.67 |
| Thecc1EG012391 | Uncharacterized protein | -1.22 | 2.89 | -9.27 | 6.66E-03 | 0.67 |
| Thecc1EG005964 | Plasma-membrane associated cation-binding protein 1,putative | -3.42 | 0.43 | -8.49 | 8.17E-03 | 0.67 |
| Thecc1EG000256 | Ferulic acid 5-hydroxylase 1,putative | -1.26 | 2.55 | -9.19 | 6.79E-03 | 0.66 |
| Thecc1EG030666 | Uncharacterized protein | -1.76 | 1.99 | -8.90 | 7.25E-03 | 0.66 |
| Thecc1EG018363 | SPFH/Band 7/PHB domain-containing membrane-associated protein family | -1.55 | 2.17 | -8.98 | 7.11E-03 | 0.66 |
| Thecc1EG005967 | Uncharacterized protein | -2.27 | 0.18 | -8.54 | 8.05E-03 | 0.66 |
| Thecc1EG045396 | Geminivirus rep interacting kinase 1 | -1.22 | 2.67 | -9.25 | 6.68E-03 | 0.66 |
| Thecc1EG041578 | Fiber protein Fb17 | -2.54 | 2.02 | -8.89 | 7.28E-03 | 0.65 |
| Thecc1EG006866 | Uncharacterized protein | -0.93 | 7.76 | -10.07 | 5.53E-03 | 0.65 |
| Thecc1EG028859 | Mitotic phosphoprotein N' end (MPPN) family protein | 0.72 | 5.24 | 10.08 | 5.51E-03 | 0.64 |
| Thecc1EG024058 | Uncharacterized protein | 0.82 | 4.46 | 9.83 | 5.79E-03 | 0.64 |
| Thecc1EG021971 | Histone superfamily protein | 1.14 | 6.16 | 10.18 | 5.38E-03 | 0.64 |
| Thecc1EG015227 | Detoxifying efflux carrier 35 | 3.47 | 1.29 | 8.80 | 7.44E-03 | 0.63 |
| Thecc1EG005843 | NAD(P)-binding Rossmann-fold superfamily protein | -1.44 | 2.99 | -9.24 | 6.69E-03 | 0.63 |
| Thecc1EG024622 | Tetratricopeptide repeat (TPR)-like superfamily protein | -0.94 | 4.03 | -9.64 | 6.09E-03 | 0.63 |
| Thecc1EG030757 | Bifunctional inhibitor/lipid-transfer protein/seed storage 2S albumin superfamily protein,putative | 2.70 | 8.44 | 10.04 | 5.57E-03 | 0.62 |
| Thecc1EG032263 | LRR and NB-ARC domains-containing disease resistance protein,putative | -1.13 | 3.64 | -9.41 | 6.45E-03 | 0.62 |
| Thecc1EG044389 | Uncharacterized protein | -1.36 | 2.04 | -9.05 | 7.00E-03 | 0.62 |
| Thecc1EG040770 | Uncharacterized protein | -1.51 | 1.89 | -8.73 | 7.58E-03 | 0.62 |
| Thecc1EG002644 | Uncharacterized protein | 1.00 | 5.55 | 10.10 | 5.50E-03 | 0.62 |
| Thecc1EG014967 | Ribosomal protein S10p/S20e family protein | 0.85 | 8.26 | 9.99 | 5.61E-03 | 0.61 |
| Thecc1EG007163 | Iq-domain 14,putative | 2.03 | 0.89 | 9.03 | 7.03E-03 | 0.61 |
| Thecc1EG042486 | Transducin family protein / WD-40 repeat family protein,putative | 0.65 | 5.57 | 10.12 | 5.48E-03 | 0.61 |
| Thecc1EG041827 | Kinase APK1B,chloroplast,putative | -1.33 | 5.58 | -10.10 | 5.50E-03 | 0.61 |
| Thecc1EG026746 | Uncharacterized protein | 1.77 | 1.55 | 8.79 | 7.47E-03 | 0.60 |
| Thecc1EG042497 | Phospholipase C 2 | -0.84 | 7.99 | -10.02 | 5.58E-03 | 0.60 |
| Thecc1EG007837 | S-locus lectin protein kinase family protein,putative | 1.43 | 3.81 | 9.62 | 6.14E-03 | 0.60 |
| Thecc1EG006650 | Plasma-membrane choline transporter family protein | 0.69 | 5.32 | 10.06 | 5.54E-03 | 0.60 |
| Thecc1EG004843 | WRKY DNA-binding protein 23,putative | -1.58 | 1.36 | -8.69 | 7.68E-03 | 0.60 |
| Thecc1EG020840 | HR-like lesion-inducing protein-related | 0.65 | 6.45 | 10.13 | 5.47E-03 | 0.60 |
| Thecc1EG003190 | Xyloglucan endotransglucosylase/hydrolase 4 | -1.10 | 4.16 | -9.54 | 6.24E-03 | 0.60 |
| Thecc1EG018941 | Malectin/receptor protein kinase family protein,putative | -1.14 | 3.98 | -9.60 | 6.16E-03 | 0.60 |
| Thecc1EG026321 | Photosystem II reaction center W protein,chloroplastic,putative | 2.23 | 0.49 | 8.46 | 8.23E-03 | 0.59 |
| Thecc1EG012584 | Zinc finger (C3HC4-type RING finger) family protein | 0.85 | 4.90 | 9.84 | 5.78E-03 | 0.59 |
| Thecc1EG022598 | Aldolase-type TIM barrel family protein | -0.69 | 5.51 | -10.06 | 5.54E-03 | 0.59 |
| Thecc1EG022849 | Alpha/beta-Hydrolases superfamily protein | 0.65 | 5.59 | 10.08 | 5.51E-03 | 0.59 |
| Thecc1EG043325 | Uncharacterized protein | -1.21 | 2.93 | -9.11 | 6.93E-03 | 0.58 |
| Thecc1EG016838 | Nodulin MtN21 /EamA-like transporter family protein,putative | 1.34 | 3.88 | 9.34 | 6.56E-03 | 0.58 |
| Thecc1EG046374 | Unknown | 1.78 | 0.29 | 8.43 | 8.27E-03 | 0.58 |
| Thecc1EG021950 | Uncharacterized protein | -1.04 | 3.99 | -9.55 | 6.22E-03 | 0.58 |
| Thecc1EG000148 | Plasma membrane intrinsic protein 3 | 1.02 | 3.53 | 9.45 | 6.39E-03 | 0.58 |
| Thecc1EG046699 | NAD(P)-binding Rossmann-fold superfamily protein,putative | 0.98 | 7.80 | 9.94 | 5.66E-03 | 0.57 |
| Thecc1EG038056 | Mitochondrial substrate carrier family protein | -0.71 | 5.23 | -10.03 | 5.58E-03 | 0.57 |
| Thecc1EG034847 | Queuine tRNA-ribosyltransferase | -0.70 | 5.26 | -10.01 | 5.60E-03 | 0.57 |
| Thecc1EG018658 | Early-responsive to dehydration stress protein (ERD4),putative | -1.27 | 2.17 | -8.86 | 7.31E-03 | 0.57 |
| Thecc1EG029935 | P(E)-nerolidol/(E,E)-geranyl linalool synthase,putative | 2.56 | 0.18 | 8.52 | 8.08E-03 | 0.56 |
| Thecc1EG031358 | Uncharacterized protein | 1.13 | 2.94 | 9.15 | 6.86E-03 | 0.56 |
| Thecc1EG031185 | Uncharacterized protein | 2.75 | 2.11 | 8.87 | 7.31E-03 | 0.56 |
| Thecc1EG026054 | Myb domain protein 113,putative | 0.82 | 4.12 | 9.55 | 6.22E-03 | 0.56 |
| Thecc1EG033233 | Uncharacterized protein | -1.42 | 1.74 | -8.74 | 7.58E-03 | 0.55 |
| Thecc1EG019492 | Uncharacterized protein | 2.82 | -0.44 | 8.23 | 8.90E-03 | 0.55 |
| Thecc1EG005645 | Uncharacterized protein | -1.45 | 1.82 | -8.83 | 7.36E-03 | 0.54 |
| Thecc1EG027602 | Uncharacterized protein | 1.36 | 1.06 | 8.71 | 7.62E-03 | 0.54 |
| Thecc1EG037922 | Heavy metal transport/detoxification superfamily protein | 1.51 | 0.99 | 8.55 | 8.04E-03 | 0.54 |
| Thecc1EG001208 | RNA-binding (RRM/RBD/RNP motifs) family protein | 0.92 | 4.05 | 9.49 | 6.30E-03 | 0.54 |
| Thecc1EG026677 | Nodulin MtN3 family protein | 1.64 | 0.59 | 8.47 | 8.23E-03 | 0.53 |
| Thecc1EG030102 | Cysteine-rich RLK 29 | -0.96 | 3.51 | -9.27 | 6.66E-03 | 0.53 |
| Thecc1EG015847 | Ureidoglycine aminohydrolase | 0.91 | 4.64 | 9.69 | 6.02E-03 | 0.53 |
| Thecc1EG046162 | Uncharacterized protein | -2.17 | 4.20 | -9.57 | 6.21E-03 | 0.53 |
| Thecc1EG043348 | Unknown | -1.11 | 2.66 | -9.01 | 7.08E-03 | 0.53 |
| Thecc1EG016635 | Cytochrome P450,putative | 1.38 | 4.17 | 9.66 | 6.09E-03 | 0.53 |
| Thecc1EG021182 | 2-oxoglutarate (2OG) and Fe(II)-dependent oxygenase superfamily protein | -1.09 | 4.70 | -9.69 | 6.02E-03 | 0.53 |
| Thecc1EG030924 | Uncharacterized protein | 2.36 | 4.51 | 9.52 | 6.27E-03 | 0.51 |
| Thecc1EG012650 | GATA type zinc finger transcription factor family protein | -1.15 | 2.61 | -8.93 | 7.21E-03 | 0.51 |
| Thecc1EG021927 | Expansin A4,ALPHA 1.6,EXPA4 | -1.64 | 5.58 | -9.79 | 5.86E-03 | 0.50 |
| Thecc1EG004547 | CASP protein GSVIVT01010992001,putative | 3.92 | -0.38 | 8.14 | 9.10E-03 | 0.50 |
| Thecc1EG014157 | C6orf149,putative | 0.73 | 5.40 | 9.90 | 5.70E-03 | 0.50 |
| Thecc1EG028016 | Nucleotide-diphospho-sugar transferases superfamily protein | -0.75 | 4.57 | -9.63 | 6.12E-03 | 0.49 |
| Thecc1EG030428 | Pentatricopeptide (PPR) repeat-containing-like protein | 1.91 | 1.94 | 8.55 | 8.05E-03 | 0.48 |
| Thecc1EG035455 | T14N5.8 protein | -0.69 | 5.57 | -9.88 | 5.72E-03 | 0.48 |
| Thecc1EG031119 | Thioredoxin | -2.02 | 3.33 | -9.00 | 7.09E-03 | 0.48 |
| Thecc1EG014703 | Pseudouridine synthase family protein | 1.78 | 1.84 | 8.57 | 8.00E-03 | 0.48 |
| Thecc1EG046152 | Uncharacterized protein | 2.38 | -0.74 | 8.08 | 9.25E-03 | 0.48 |
| Thecc1EG005722 | Ras 5 | 0.70 | 7.33 | 9.83 | 5.79E-03 | 0.48 |
| Thecc1EG014624 | Ribosomal protein L22p/L17e family protein | 0.70 | 8.70 | 9.69 | 6.02E-03 | 0.47 |
| Thecc1EG024725 | Elongation factor family protein | -0.77 | 4.68 | -9.61 | 6.14E-03 | 0.47 |
| Thecc1EG025025 | SC35-like splicing factor 33 | 0.76 | 5.70 | 9.89 | 5.72E-03 | 0.47 |
| Thecc1EG035433 | Alcohol dehydrogenase 1 | -0.85 | 5.12 | -9.75 | 5.93E-03 | 0.47 |
| Thecc1EG037260 | Uncharacterized protein | 0.67 | 5.96 | 9.90 | 5.71E-03 | 0.47 |
| Thecc1EG046018 | Uncharacterized protein | -1.06 | 2.84 | -8.94 | 7.20E-03 | 0.47 |
| Thecc1EG025733 | TRNA methyltransferase | -1.11 | 3.11 | -9.03 | 7.03E-03 | 0.47 |
| Thecc1EG046945 | NBS type disease resistance protein,putative | -1.50 | 1.42 | -8.51 | 8.10E-03 | 0.46 |
| Thecc1EG004882 | Cysteine-rich RLK (RECEPTOR-like protein kinase) 8,putative | -1.51 | 1.46 | -8.55 | 8.04E-03 | 0.46 |
| Thecc1EG024615 | 6-phosphogluconate dehydrogenase family protein | 1.15 | 2.95 | 8.94 | 7.19E-03 | 0.46 |
| Thecc1EG008737 | Histidine kinase 1 | -0.66 | 5.65 | -9.87 | 5.72E-03 | 0.46 |
| Thecc1EG006452 | Pentatricopeptide repeat (PPR) superfamily protein | -1.50 | 1.59 | -8.43 | 8.27E-03 | 0.46 |
| Thecc1EG029942 | Glucose-fructose oxidoreductase domain-containing protein 2,putative | 2.88 | 1.30 | 8.43 | 8.27E-03 | 0.46 |
| Thecc1EG031037 | Unknown | 3.42 | -1.43 | 7.98 | 9.53E-03 | 0.45 |
| Thecc1EG011899 | Uncharacterized protein | -1.08 | 3.25 | -9.11 | 6.93E-03 | 0.45 |
| Thecc1EG037297 | Alpha/beta-Hydrolases superfamily protein | -1.03 | 3.83 | -9.29 | 6.66E-03 | 0.45 |
| Thecc1EG046982 | Glutathione peroxidase 4 | -0.79 | 5.68 | -9.86 | 5.74E-03 | 0.45 |
| Thecc1EG020955 | Cyclopropane-fatty-acyl-phospholipid synthase | 0.73 | 5.10 | 9.73 | 5.95E-03 | 0.45 |
| Thecc1EG025959 | Leucine-rich repeat containing protein | 2.73 | -0.89 | 8.01 | 9.46E-03 | 0.44 |
| Thecc1EG005410 | Shaggy-like protein kinase 32 | 0.66 | 5.72 | 9.79 | 5.86E-03 | 0.44 |
| Thecc1EG041769 | NAD(P)-binding Rossmann-fold superfamily protein,putative | -3.30 | 2.02 | -8.62 | 7.84E-03 | 0.43 |
| Thecc1EG031168 | Unknown | -0.76 | 7.14 | -9.75 | 5.93E-03 | 0.43 |
| Thecc1EG042523 | Malectin/receptor protein kinase family protein | 1.13 | 5.09 | 9.69 | 6.02E-03 | 0.42 |
| Thecc1EG008073 | Alpha/beta-Hydrolases superfamily protein | -1.15 | 6.42 | -9.77 | 5.88E-03 | 0.42 |
| Thecc1EG004748 | F5K24.2-like protein | -1.20 | 2.00 | -8.63 | 7.82E-03 | 0.42 |
| Thecc1EG025825 | Sulfotransferase 2A,putative | -4.13 | 6.31 | -9.36 | 6.53E-03 | 0.41 |
| Thecc1EG035110 | Androgen induced inhibitor of proliferation (As3) / pds5,putative | -1.51 | 3.82 | -9.12 | 6.90E-03 | 0.40 |
| Thecc1EG028881 | Pleiotropic drug resistance 1 | -1.74 | 2.19 | -8.57 | 8.01E-03 | 0.40 |
| Thecc1EG015485 | Uncharacterized protein | -2.42 | 0.47 | -8.18 | 9.01E-03 | 0.40 |
| Thecc1EG040413 | Uncharacterized protein | -1.49 | 3.20 | -8.90 | 7.25E-03 | 0.39 |
| Thecc1EG004751 | Uncharacterized protein | -3.63 | 2.65 | -8.52 | 8.09E-03 | 0.39 |
| Thecc1EG035192 | Amino acid permease | -1.85 | 5.18 | -9.34 | 6.56E-03 | 0.39 |
| Thecc1EG010525 | Uncharacterized protein | -3.43 | 1.41 | -8.23 | 8.91E-03 | 0.39 |
| Thecc1EG015766 | Pyruvate kinase isozyme G,chloroplastic | 1.28 | 5.84 | 9.70 | 6.02E-03 | 0.39 |
| Thecc1EG019822 | Cc-nbs-lrr resistance-like protein | -3.73 | 0.15 | -8.07 | 9.30E-03 | 0.38 |
| Thecc1EG004504 | Gamma histone variant H2AX | 0.85 | 4.74 | 9.45 | 6.37E-03 | 0.37 |
| Thecc1EG005580 | Uncharacterized protein | -4.12 | -1.49 | -7.84 | 9.93E-03 | 0.37 |
| Thecc1EG020884 | Phosphoenolpyruvate carboxylase kinase 1,putative | 1.84 | 3.44 | 8.98 | 7.11E-03 | 0.37 |
| Thecc1EG017179 | Ribosomal protein S21 family protein | 0.93 | 4.63 | 9.42 | 6.42E-03 | 0.37 |
| Thecc1EG006421 | Ribosomal protein L10 family protein | 0.79 | 6.35 | 9.71 | 6.02E-03 | 0.37 |
| Thecc1EG005930 | Thioredoxin superfamily protein | -0.99 | 5.01 | -9.56 | 6.21E-03 | 0.36 |
| Thecc1EG005477 | Cytochrome P450 family protein | -0.93 | 4.33 | -9.36 | 6.53E-03 | 0.36 |
| Thecc1EG000295 | Peroxidase superfamily protein | 1.58 | 3.88 | 9.20 | 6.79E-03 | 0.36 |
| Thecc1EG011432 | Unknown | -3.21 | 0.52 | -8.14 | 9.10E-03 | 0.36 |
| Thecc1EG002018 | Uncharacterized protein | 1.00 | 4.57 | 9.39 | 6.49E-03 | 0.35 |
| Thecc1EG016905 | Pectin lyase-like superfamily protein | 1.06 | 2.43 | 8.68 | 7.70E-03 | 0.35 |
| Thecc1EG004097 | 2-oxoglutarate and Fe(II)-dependent oxygenase superfamily protein | -1.63 | 4.60 | -9.07 | 6.98E-03 | 0.35 |
| Thecc1EG042099 | Uncharacterized protein | 2.42 | 0.72 | 8.16 | 9.06E-03 | 0.34 |
| Thecc1EG020042 | Calcium-dependent lipid-binding (CaLB domain) plant phosphoribosyltransferase family protein | 1.50 | 2.36 | 8.52 | 8.08E-03 | 0.34 |
| Thecc1EG024594 | UDP-glucosyltransferase,putative | -1.68 | 4.40 | -9.23 | 6.69E-03 | 0.34 |
| Thecc1EG038275 | BEL1-like homeodomain protein 8,putative | 0.88 | 4.03 | 9.17 | 6.82E-03 | 0.34 |
| Thecc1EG024084 | Uncharacterized protein | 1.71 | 3.28 | 8.75 | 7.55E-03 | 0.34 |
| Thecc1EG006615 | Uncharacterized protein | 1.99 | -0.28 | 7.91 | 9.76E-03 | 0.34 |
| Thecc1EG005902 | Uncharacterized protein | 0.94 | 4.25 | 9.25 | 6.67E-03 | 0.34 |
| Thecc1EG005258 | Proline iminopeptidase,putative | -0.67 | 6.66 | -9.65 | 6.09E-03 | 0.33 |
| Thecc1EG035331 | Peroxisomal membrane 22 kDa (Mpv17/PMP22) family protein,putative | 0.65 | 5.28 | 9.59 | 6.18E-03 | 0.33 |
| Thecc1EG036933 | Integrase-type DNA-binding superfamily protein | 0.87 | 3.98 | 8.98 | 7.11E-03 | 0.33 |
| Thecc1EG005751 | Uncharacterized protein | 2.39 | 0.26 | 8.14 | 9.10E-03 | 0.32 |
| Thecc1EG033433 | Short-chain dehydrogenase-reductase B | 0.89 | 3.37 | 9.03 | 7.03E-03 | 0.32 |
| Thecc1EG033901 | Uncharacterized protein | -0.94 | 6.39 | -9.63 | 6.12E-03 | 0.32 |
| Thecc1EG032078 | Trichome birefringence-like 42 | 1.76 | 1.34 | 8.36 | 8.49E-03 | 0.32 |
| Thecc1EG030910 | GDSL-like Lipase/Acylhydrolase superfamily protein,putative | 2.18 | -0.10 | 7.95 | 9.64E-03 | 0.32 |
| Thecc1EG012165 | Adenine nucleotide alpha hydrolases-like superfamily protein,putative | -1.18 | 4.49 | -9.34 | 6.56E-03 | 0.31 |
| Thecc1EG035474 | 15-cis-zeta-carotene isomerase | -0.75 | 4.90 | -9.42 | 6.42E-03 | 0.31 |
| Thecc1EG030997 | Unknown | 1.24 | 3.85 | 9.15 | 6.86E-03 | 0.31 |
| Thecc1EG037455 | Transcription factor bHLH69,putative | 0.73 | 4.95 | 9.41 | 6.45E-03 | 0.31 |
| Thecc1EG040572 | Cysteine-rich RLK (RECEPTOR-like protein kinase) 8 | -1.49 | 0.97 | -8.11 | 9.14E-03 | 0.31 |
| Thecc1EG028702 | AT hook motif-containing protein,putative | 0.74 | 4.52 | 9.33 | 6.58E-03 | 0.31 |
| Thecc1EG016894 | Proteasome assembly chaperone 3 | 0.77 | 4.93 | 9.46 | 6.37E-03 | 0.30 |
| Thecc1EG005000 | Serine-type endopeptidase inhibitor | 0.93 | 4.44 | 9.27 | 6.66E-03 | 0.30 |
| Thecc1EG020198 | Xyloglucan endotransglucosylase/hydrolase 16 | 1.62 | 6.86 | 9.54 | 6.25E-03 | 0.29 |
| Thecc1EG037612 | UDP-Glycosyltransferase superfamily protein | -1.79 | 5.18 | -9.43 | 6.40E-03 | 0.29 |
| Thecc1EG046679 | UDP-glucosyl transferase 85A2 | 1.63 | 8.54 | 9.31 | 6.62E-03 | 0.29 |
| Thecc1EG006173 | Unknown | -1.75 | 1.37 | -8.18 | 9.02E-03 | 0.29 |
| Thecc1EG004891 | HCO3- transporter family | -2.48 | 2.95 | -8.86 | 7.31E-03 | 0.29 |
| Thecc1EG005643 | Leucine-rich repeat receptor-like protein kinase family protein,putative | -1.51 | 0.71 | -8.14 | 9.10E-03 | 0.29 |
| Thecc1EG004521 | NAD(P)-binding Rossmann-fold superfamily protein | -1.36 | 5.52 | -9.51 | 6.27E-03 | 0.29 |
| Thecc1EG029199 | Synaptobrevin family protein | 0.88 | 5.47 | 9.51 | 6.27E-03 | 0.28 |
| Thecc1EG006799 | MATE efflux family protein | -0.96 | 4.21 | -9.03 | 7.03E-03 | 0.28 |
| Thecc1EG025244 | Uncharacterized protein | 1.68 | 0.90 | 8.06 | 9.32E-03 | 0.28 |
| Thecc1EG033470 | Unknown | -2.12 | 2.09 | -8.35 | 8.50E-03 | 0.28 |
| Thecc1EG021047 | Uncharacterized protein | 2.69 | -0.13 | 7.83 | 9.94E-03 | 0.28 |
| Thecc1EG010882 | Sulfate transporter 91 | -1.72 | 0.58 | -7.99 | 9.53E-03 | 0.27 |
| Thecc1EG012973 | Glutamine synthase clone R1,1,ATGLN1,1 | 0.67 | 6.18 | 9.53 | 6.25E-03 | 0.27 |
| Thecc1EG022162 | Unknown | 1.80 | -0.21 | 7.85 | 9.89E-03 | 0.27 |
| Thecc1EG034983 | AGD2-like defense response protein 1 | 1.70 | 0.80 | 7.94 | 9.67E-03 | 0.27 |
| Thecc1EG000877 | Cytochrome c oxidase-related | 0.63 | 6.78 | 9.51 | 6.27E-03 | 0.27 |
| Thecc1EG039126 | Plasma membrane ATPase 4 | -1.51 | 1.48 | -8.13 | 9.11E-03 | 0.26 |
| Thecc1EG003024 | Uncharacterized protein | 1.26 | 4.15 | 9.12 | 6.90E-03 | 0.26 |
| Thecc1EG025991 | Uncharacterized protein | -1.45 | 1.41 | -8.09 | 9.22E-03 | 0.26 |
| Thecc1EG037136 | BURP domain-containing protein | 1.32 | 3.30 | 8.85 | 7.33E-03 | 0.26 |
| Thecc1EG006099 | Exostosin family protein | -0.88 | 3.99 | -9.09 | 6.95E-03 | 0.25 |
| Thecc1EG038478 | AFG1-like ATPase family protein | -0.94 | 6.30 | -9.49 | 6.30E-03 | 0.25 |
| Thecc1EG041603 | Fatty acid desaturase 8 | -1.52 | 0.92 | -8.04 | 9.38E-03 | 0.25 |
| Thecc1EG009956 | Uncharacterized protein | 0.96 | 3.57 | 8.79 | 7.47E-03 | 0.24 |
| Thecc1EG018959 | Reversibly glycosylated polypeptide 1 | -0.71 | 5.26 | -9.38 | 6.49E-03 | 0.24 |
| Thecc1EG041420 | F-box/RNI-like superfamily protein | -0.76 | 4.20 | -9.12 | 6.90E-03 | 0.24 |
| Thecc1EG019574 | Receptor like protein 6,putative | -2.02 | -0.08 | -7.83 | 9.94E-03 | 0.24 |
| Thecc1EG027120 | ATP-dependent Clp protease | 1.19 | 5.75 | 9.43 | 6.40E-03 | 0.24 |
| Thecc1EG032195 | Uncharacterized protein | -2.47 | 0.14 | -7.84 | 9.94E-03 | 0.23 |
| Thecc1EG046199 | Malectin/receptor protein kinase family protein | 1.50 | 1.83 | 8.28 | 8.73E-03 | 0.23 |
| Thecc1EG014680 | HVA22 D | -1.09 | 3.71 | -8.84 | 7.35E-03 | 0.23 |
| Thecc1EG011741 | Galactose oxidase/kelch repeat superfamily protein | -0.61 | 5.56 | -9.44 | 6.39E-03 | 0.23 |
| Thecc1EG036225 | Rieske domain-containing protein | 0.98 | 4.46 | 9.24 | 6.69E-03 | 0.23 |
| Thecc1EG004056 | Ethylene response factor 1 | 2.20 | 0.46 | 7.83 | 9.95E-03 | 0.21 |
| Thecc1EG011929 | Peptidase S24/S26A/S26B/S26C family protein | -0.65 | 7.06 | -9.38 | 6.50E-03 | 0.20 |
| Thecc1EG007920 | HXXXD-type acyl-transferase family protein,putative | -1.52 | 1.50 | -8.34 | 8.54E-03 | 0.20 |
| Thecc1EG028960 | Leucine-rich repeat protein kinase family protein,putative | -1.36 | 2.44 | -8.40 | 8.37E-03 | 0.19 |
| Thecc1EG010858 | Chaperone DnaJ-domain superfamily protein,putative | 3.14 | 0.84 | 7.88 | 9.83E-03 | 0.19 |
| Thecc1EG007249 | Uncharacterized protein | 1.04 | 6.79 | 9.37 | 6.52E-03 | 0.19 |
| Thecc1EG010389 | Galactinol synthase 4 | -0.67 | 4.92 | -9.26 | 6.67E-03 | 0.18 |
| Thecc1EG033758 | Uncharacterized protein | -1.58 | 0.80 | -7.92 | 9.74E-03 | 0.18 |
| Thecc1EG005375 | Acetyl Co-enzyme a carboxylase carboxyltransferase alpha subunit | -1.22 | 4.89 | -9.04 | 7.03E-03 | 0.18 |
| Thecc1EG006178 | Bile acid sodium symporter/ transporter | 0.68 | 6.52 | 9.37 | 6.52E-03 | 0.17 |
| Thecc1EG007840 | S-locus lectin protein kinase family protein,putative | 1.53 | 5.42 | 9.30 | 6.65E-03 | 0.17 |
| Thecc1EG016233 | Tubulin beta 8 | 0.66 | 5.33 | 9.22 | 6.72E-03 | 0.17 |
| Thecc1EG032041 | Leucine-rich repeat transmembrane protein kinase | -1.54 | 4.99 | -9.18 | 6.80E-03 | 0.17 |
| Thecc1EG004688 | Ribosomal protein L18 | 0.64 | 6.72 | 9.33 | 6.56E-03 | 0.16 |
| Thecc1EG016647 | Homeodomain-like superfamily protein,putative | 1.14 | 2.90 | 8.46 | 8.23E-03 | 0.16 |
| Thecc1EG042591 | Uncharacterized protein | 1.10 | 3.31 | 8.61 | 7.88E-03 | 0.16 |
| Thecc1EG005795 | Ferredoxin-related | 1.15 | 3.10 | 8.45 | 8.25E-03 | 0.16 |
| Thecc1EG013384 | Glutamate decarboxylase 5 | -0.73 | 9.40 | -9.07 | 6.98E-03 | 0.16 |
| Thecc1EG037562 | Acyl-CoA-binding 6-like protein | 2.10 | 2.49 | 8.12 | 9.13E-03 | 0.15 |
| Thecc1EG014615 | Uridine kinase/uracil phosphoribosyltransferase 1 | 2.64 | 1.37 | 7.89 | 9.80E-03 | 0.15 |
| Thecc1EG025717 | Uncharacterized protein | -0.99 | 7.14 | -9.28 | 6.66E-03 | 0.14 |
| Thecc1EG020919 | COP1-interacting protein-related,putative | -0.77 | 6.84 | -9.28 | 6.66E-03 | 0.14 |
| Thecc1EG026425 | Unknown | 1.42 | 1.35 | 8.05 | 9.35E-03 | 0.14 |
| Thecc1EG014724 | ARM repeat superfamily protein | 0.71 | 5.57 | 9.27 | 6.66E-03 | 0.13 |
| Thecc1EG015180 | RNA polymerase III subunit RPC82 family protein,putative | -0.76 | 5.39 | -9.26 | 6.67E-03 | 0.13 |
| Thecc1EG022891 | NDR1/HIN1-like 1 | -1.45 | 6.46 | -9.27 | 6.66E-03 | 0.13 |
| Thecc1EG002152 | PHD finger family protein / bromo-adjacent (BAH) domain-containing protein | -0.91 | 5.57 | -9.23 | 6.69E-03 | 0.12 |
| Thecc1EG006175 | Uncharacterized protein | 1.47 | 1.59 | 8.05 | 9.37E-03 | 0.12 |
| Thecc1EG006375 | Carbohydrate transporter/ sugar porter/ transporter | 1.10 | 4.94 | 9.07 | 6.98E-03 | 0.12 |
| Thecc1EG028130 | DNA repair protein recA | -0.93 | 3.29 | -8.46 | 8.23E-03 | 0.11 |
| Thecc1EG037984 | Histone H2A protein 9 | 0.60 | 5.93 | 9.27 | 6.66E-03 | 0.11 |
| Thecc1EG000904 | Phosphoenolpyruvate carboxykinase 1 | -2.85 | 6.10 | -9.10 | 6.93E-03 | 0.11 |
| Thecc1EG015432 | Uncharacterized protein | 0.79 | 5.38 | 9.17 | 6.82E-03 | 0.09 |
| Thecc1EG031514 | Cytochrome C oxidase copper chaperone | 1.51 | 3.43 | 8.45 | 8.23E-03 | 0.09 |
| Thecc1EG037776 | Hexokinase 1 | -0.82 | 5.41 | -9.15 | 6.86E-03 | 0.08 |
| Thecc1EG008493 | Senescence-associated gene 29 | -2.31 | 1.49 | -7.85 | 9.89E-03 | 0.08 |
| Thecc1EG020016 | Uncharacterized protein | -0.74 | 4.75 | -8.98 | 7.12E-03 | 0.08 |
| Thecc1EG028840 | Methyl esterase 12 | -0.88 | 5.35 | -9.14 | 6.88E-03 | 0.07 |
| Thecc1EG015835 | Calcineurin B-like protein 1 | -0.78 | 4.58 | -8.98 | 7.11E-03 | 0.07 |
| Thecc1EG042404 | Tumour suppressor protein Gltscr2 | -0.67 | 6.53 | -9.19 | 6.79E-03 | 0.07 |
| Thecc1EG017226 | Ribosomal protein large subunit 27 | 0.67 | 6.20 | 9.19 | 6.79E-03 | 0.07 |
| Thecc1EG005755 | Uncharacterized protein | 2.31 | 7.28 | 9.06 | 6.98E-03 | 0.06 |
| Thecc1EG007964 | Transferases,folic acid binding | 1.03 | 2.82 | 8.22 | 8.92E-03 | 0.05 |
| Thecc1EG019790 | Uncharacterized protein | -1.39 | 5.09 | -8.96 | 7.16E-03 | 0.05 |
| Thecc1EG016682 | Uncharacterized protein | -0.70 | 5.18 | -9.06 | 6.98E-03 | 0.05 |
| Thecc1EG012311 | Cytochrome P450 superfamily protein | 1.92 | 4.43 | 8.84 | 7.35E-03 | 0.04 |
| Thecc1EG027158 | Phospholipase D alpha 2 | 0.74 | 8.88 | 8.93 | 7.21E-03 | 0.04 |
| Thecc1EG014097 | RING/U-box superfamily protein | 0.97 | 3.17 | 8.31 | 8.63E-03 | 0.03 |
| Thecc1EG044487 | Glutathione S-transferase 2 | -0.73 | 7.82 | -9.01 | 7.08E-03 | 0.03 |
| Thecc1EG034040 | Leucine carboxyl methyltransferase | 1.28 | 2.48 | 8.13 | 9.11E-03 | 0.02 |
| Thecc1EG011824 | 2-oxoacid dehydrogenases acyltransferase family protein | 0.60 | 5.44 | 9.06 | 6.99E-03 | 0.02 |
| Thecc1EG004303 | Kinase superfamily protein | -0.63 | 4.69 | -8.95 | 7.16E-03 | 0.02 |
| Thecc1EG037706 | Phospholipase A2 family protein | 1.20 | 2.80 | 8.25 | 8.84E-03 | 0.01 |
| Thecc1EG006713 | CC-NBS-LRR class disease resistance protein,putative | 1.63 | 3.10 | 8.36 | 8.49E-03 | 0.01 |
| Thecc1EG025664 | Benzoyl coenzyme A: Benzyl alcohol benzoyl transferase | 2.33 | -0.01 | 7.87 | 9.84E-03 | 0.01 |
| Thecc1EG045394 | Uncharacterized protein | -0.86 | 3.55 | -8.40 | 8.36E-03 | 0.01 |
| Thecc1EG021491 | Fcf2 pre-rRNA processing protein | 0.68 | 5.73 | 9.08 | 6.97E-03 | 0.01 |
| Thecc1EG045585 | RING/U-box superfamily protein | -1.23 | 3.37 | -8.19 | 8.98E-03 | 0.01 |
| Thecc1EG014112 | Uncharacterized protein | -0.76 | 4.65 | -8.91 | 7.25E-03 | 0.00 |
| Thecc1EG037748 | Phosphotyrosine protein phosphatases superfamily protein | -1.04 | 6.17 | -9.07 | 6.98E-03 | 0.00 |
| Thecc1EG025624 | Ribosomal protein L17 family protein | 0.71 | 4.85 | 8.87 | 7.31E-03 | -0.01 |
| Thecc1EG021266 | Ribosomal protein L19e family protein | 0.67 | 8.56 | 8.88 | 7.29E-03 | -0.01 |
| Thecc1EG026055 | Uncharacterized protein | -1.14 | 4.70 | -8.78 | 7.49E-03 | -0.01 |
| Thecc1EG012254 | Transmembrane Fragile-X-F-associated protein | -0.67 | 4.88 | -8.89 | 7.28E-03 | -0.03 |
| Thecc1EG005613 | GATA transcription factor 2,putative | 1.16 | 4.66 | 8.63 | 7.83E-03 | -0.03 |
| Thecc1EG006184 | Alpha/beta-Hydrolases superfamily protein | -0.70 | 5.21 | -8.97 | 7.13E-03 | -0.04 |
| Thecc1EG016484 | Glycine-rich protein family | 0.63 | 4.72 | 8.83 | 7.37E-03 | -0.04 |
| Thecc1EG015678 | Serine/threonine-protein kinase WNK-related,putative | -0.82 | 3.59 | -8.36 | 8.49E-03 | -0.04 |
| Thecc1EG027875 | Leucine-rich repeat protein kinase family protein | -1.23 | 3.13 | -8.21 | 8.93E-03 | -0.05 |
| Thecc1EG035094 | Uncharacterized protein | -0.66 | 6.50 | -9.00 | 7.08E-03 | -0.05 |
| Thecc1EG011333 | Ras-related small GTP-binding family protein,putative | -1.04 | 3.95 | -8.45 | 8.25E-03 | -0.05 |
| Thecc1EG031202 | S-locus lectin protein kinase family protein | -1.05 | 5.29 | -8.93 | 7.21E-03 | -0.05 |
| Thecc1EG020203 | Indole-3-acetate beta-D-glucosyltransferase,putative | -0.58 | 5.83 | -8.98 | 7.11E-03 | -0.06 |
| Thecc1EG027357 | Uncharacterized protein | 1.09 | 2.75 | 8.12 | 9.13E-03 | -0.07 |
| Thecc1EG004533 | Kinase interacting family protein,putative | -1.42 | 5.80 | -8.91 | 7.25E-03 | -0.07 |
| Thecc1EG016421 | UDP-Glycosyltransferase superfamily protein | 0.89 | 5.43 | 8.90 | 7.25E-03 | -0.07 |
| Thecc1EG015420 | Gamma-irradiation and mitomycin c induced 1,putative | -0.80 | 7.10 | -8.92 | 7.23E-03 | -0.07 |
| Thecc1EG005452 | Subtilase family protein | -1.83 | 6.66 | -8.89 | 7.28E-03 | -0.08 |
| Thecc1EG034394 | Hedgehog receptor,putative | -1.24 | 3.12 | -8.12 | 9.14E-03 | -0.08 |
| Thecc1EG011779 | NAD(P)-linked oxidoreductase superfamily protein | 1.93 | 4.22 | 8.51 | 8.10E-03 | -0.08 |
| Thecc1EG005123 | Uncharacterized protein | 0.75 | 7.47 | 8.84 | 7.35E-03 | -0.08 |
| Thecc1EG014935 | Haloacid dehalogenase-like hydrolase superfamily protein | -0.82 | 4.29 | -8.57 | 8.01E-03 | -0.08 |
| Thecc1EG026986 | Uncharacterized protein | -1.10 | 2.83 | -7.94 | 9.69E-03 | -0.09 |
| Thecc1EG016391 | Galactose mutarotase-like superfamily protein | -1.27 | 4.20 | -8.44 | 8.25E-03 | -0.10 |
| Thecc1EG033316 | Uncharacterized protein | 0.77 | 4.56 | 8.75 | 7.55E-03 | -0.11 |
| Thecc1EG034939 | AP2 4,putative | -0.58 | 7.53 | -8.83 | 7.36E-03 | -0.12 |
| Thecc1EG010189 | Uncharacterized protein | 3.22 | 3.12 | 7.98 | 9.53E-03 | -0.13 |
| Thecc1EG022082 | Leucine-rich repeat protein kinase family protein | 0.82 | 6.33 | 8.86 | 7.31E-03 | -0.13 |
| Thecc1EG014660 | Uncharacterized protein | -1.13 | 4.96 | -8.67 | 7.71E-03 | -0.13 |
| Thecc1EG029035 | Transducin family protein / WD-40 repeat family protein | 1.51 | 3.23 | 8.06 | 9.30E-03 | -0.13 |
| Thecc1EG017002 | Staphylococcal nuclease | -1.07 | 4.54 | -8.66 | 7.74E-03 | -0.13 |
| Thecc1EG046695 | RAD-like 6 | -1.02 | 3.65 | -8.23 | 8.91E-03 | -0.13 |
| Thecc1EG046759 | NAD(P)-linked oxidoreductase superfamily protein | 1.38 | 8.09 | 8.73 | 7.58E-03 | -0.14 |
| Thecc1EG004791 | Uncharacterized protein | 0.70 | 5.34 | 8.77 | 7.53E-03 | -0.15 |
| Thecc1EG004503 | NAD(P)-linked oxidoreductase superfamily protein | -0.93 | 3.48 | -8.12 | 9.14E-03 | -0.15 |
| Thecc1EG034424 | Glyceraldehyde-3-phosphate dehydrogenase B subunit | 1.78 | 3.50 | 8.39 | 8.38E-03 | -0.16 |
| Thecc1EG004925 | Endonuclease or glycosyl hydrolase with C2H2-type zinc finger domain,putative | -0.91 | 4.44 | -8.46 | 8.23E-03 | -0.16 |
| Thecc1EG000268 | Aldehyde dehydrogenase 3F1 | 1.85 | 3.80 | 8.33 | 8.55E-03 | -0.16 |
| Thecc1EG011009 | Plant cadmium resistance 2,putative | 1.07 | 3.27 | 8.10 | 9.18E-03 | -0.16 |
| Thecc1EG001196 | Uncharacterized protein | 1.05 | 3.09 | 7.96 | 9.62E-03 | -0.16 |
| Thecc1EG005104 | No lysine kinase 6,putative | -1.02 | 3.12 | -7.96 | 9.62E-03 | -0.18 |
| Thecc1EG007027 | Alpha/beta-Hydrolases superfamily protein,putative | 1.13 | 5.73 | 8.76 | 7.53E-03 | -0.18 |
| Thecc1EG046680 | UDP-glucosyl transferase 85A2 | 0.81 | 7.43 | 8.68 | 7.69E-03 | -0.18 |
| Thecc1EG008810 | Cell wall integrity and stress response component 1 | 0.74 | 5.93 | 8.77 | 7.53E-03 | -0.19 |
| Thecc1EG021199 | Uveal autoantigen with coiled-coil domains and ankyrin repeats | 0.65 | 4.89 | 8.54 | 8.05E-03 | -0.19 |
| Thecc1EG010013 | Glycine-rich protein,putative | 0.59 | 6.05 | 8.76 | 7.53E-03 | -0.20 |
| Thecc1EG002307 | Thymidine kinase | 0.85 | 3.52 | 8.01 | 9.46E-03 | -0.20 |
| Thecc1EG016336 | Heavy metal transport/detoxification superfamily protein | -0.82 | 3.34 | -8.12 | 9.13E-03 | -0.20 |
| Thecc1EG024183 | F5I14.9 protein | 1.06 | 5.44 | 8.69 | 7.67E-03 | -0.21 |
| Thecc1EG004667 | Photolyase/blue-light receptor 2 | -0.65 | 6.22 | -8.73 | 7.58E-03 | -0.21 |
| Thecc1EG016883 | L-aspartate oxidase | -0.81 | 5.47 | -8.71 | 7.63E-03 | -0.21 |
| Thecc1EG015786 | Uncharacterized protein | 0.75 | 3.82 | 8.25 | 8.84E-03 | -0.21 |
| Thecc1EG004657 | NAD(P)-binding Rossmann-fold superfamily protein | -1.24 | 6.40 | -8.71 | 7.62E-03 | -0.21 |
| Thecc1EG023045 | Uncharacterized protein | 0.58 | 6.34 | 8.72 | 7.58E-03 | -0.22 |
| Thecc1EG038829 | Zinc finger protein CONSTANS-LIKE 5,putative | -0.63 | 6.70 | -8.60 | 7.89E-03 | -0.22 |
| Thecc1EG005593 | Unknown | -1.55 | 3.09 | -7.85 | 9.89E-03 | -0.22 |
| Thecc1EG030099 | Cysteine-rich RLK 29,putative | -1.45 | 6.47 | -8.68 | 7.68E-03 | -0.22 |
| Thecc1EG029725 | 3R-hydroxymyristoyl- dehydratase-hydroxymyristoyl ACP dehydrase | 0.65 | 4.73 | 8.46 | 8.23E-03 | -0.23 |
| Thecc1EG046841 | Aldolase-type TIM barrel family protein | -0.74 | 4.81 | -8.52 | 8.09E-03 | -0.23 |
| Thecc1EG004444 | Circadian clock coupling factor ZGT | -0.81 | 5.93 | -8.67 | 7.71E-03 | -0.23 |
| Thecc1EG005434 | Uncharacterized protein | -1.08 | 5.66 | -8.66 | 7.72E-03 | -0.23 |
| Thecc1EG030499 | Peroxin 5 | -0.86 | 6.10 | -8.68 | 7.68E-03 | -0.24 |
| Thecc1EG008137 | Switch 2 | -0.67 | 4.99 | -8.54 | 8.06E-03 | -0.25 |
| Thecc1EG022061 | Run and tbc1 domain containing 3,plant,putative | -0.73 | 4.47 | -8.37 | 8.45E-03 | -0.25 |
| Thecc1EG018651 | Nucleus-like protein | 0.70 | 8.69 | 8.47 | 8.23E-03 | -0.25 |
| Thecc1EG025852 | Leucine-rich repeat protein kinase family protein | -0.72 | 5.93 | -8.65 | 7.75E-03 | -0.25 |
| Thecc1EG042726 | Pathogenesis-related thaumatin superfamily protein | 0.62 | 6.09 | 8.65 | 7.74E-03 | -0.26 |
| Thecc1EG016536 | Uncharacterized protein | 0.68 | 4.47 | 8.36 | 8.49E-03 | -0.26 |
| Thecc1EG027831 | To encode a PR protein,Belongs to the plant thionin family with the following members:,putative | -0.90 | 7.22 | -8.59 | 7.92E-03 | -0.26 |
| Thecc1EG020831 | Uncharacterized protein | 0.97 | 4.77 | 8.49 | 8.17E-03 | -0.27 |
| Thecc1EG007505 | CAMP-regulated phosphoprotein 19-related protein | 0.58 | 7.85 | 8.51 | 8.10E-03 | -0.28 |
| Thecc1EG020925 | Cysteine-rich RLK 29,putative | 1.82 | 3.42 | 8.06 | 9.30E-03 | -0.28 |
| Thecc1EG037697 | N-MYC downregulated-like 1 | -0.57 | 6.16 | -8.62 | 7.83E-03 | -0.28 |
| Thecc1EG042017 | Uncharacterized protein | -0.75 | 5.40 | -8.53 | 8.08E-03 | -0.29 |
| Thecc1EG034043 | Ribosomal protein L27 family protein | 0.57 | 5.27 | 8.55 | 8.04E-03 | -0.29 |
| Thecc1EG039452 | Chloroplast import apparatus 2,putative | 0.61 | 4.92 | 8.44 | 8.25E-03 | -0.29 |
| Thecc1EG012750 | Pathogenesis-related thaumatin superfamily protein | 0.82 | 7.87 | 8.46 | 8.23E-03 | -0.31 |
| Thecc1EG000979 | Uncharacterized protein | 0.96 | 3.21 | 7.85 | 9.89E-03 | -0.31 |
| Thecc1EG010460 | Pyruvate phosphate dikinase | -0.57 | 7.32 | -8.54 | 8.05E-03 | -0.31 |
| Thecc1EG028563 | Patellin 1,putative | 0.74 | 4.30 | 8.19 | 8.98E-03 | -0.31 |
| Thecc1EG025661 | Benzoyl coenzyme A: Benzyl alcohol benzoyl transferase | 2.34 | 3.99 | 8.16 | 9.06E-03 | -0.31 |
| Thecc1EG031538 | Dynamin related protein 5A | 0.92 | 3.79 | 7.91 | 9.76E-03 | -0.31 |
| Thecc1EG028612 | BRCT domain-containing DNA repair protein,putative | -0.60 | 4.90 | -8.41 | 8.32E-03 | -0.32 |
| Thecc1EG019500 | Zinc-finger domain of monoamine-oxidase A repressor R1 protein,putative | 0.75 | 4.14 | 8.15 | 9.06E-03 | -0.32 |
| Thecc1EG043022 | Histone superfamily protein | 0.74 | 4.17 | 8.11 | 9.14E-03 | -0.33 |
| Thecc1EG038089 | Ribosomal protein L36,putative | 0.67 | 4.66 | 8.31 | 8.63E-03 | -0.34 |
| Thecc1EG003621 | Sodium/calcium exchanger family protein / calcium-binding EF hand family protein | -1.41 | 4.68 | -8.41 | 8.32E-03 | -0.34 |
| Thecc1EG029496 | Prefoldin 6 | 0.62 | 5.07 | 8.44 | 8.25E-03 | -0.34 |
| Thecc1EG041887 | Histone superfamily protein | 0.57 | 7.79 | 8.42 | 8.28E-03 | -0.35 |
| Thecc1EG037077 | LJRHL1-like 1 | -0.77 | 4.41 | -8.22 | 8.91E-03 | -0.35 |
| Thecc1EG004210 | F-box/LRR-repeat protein 17 | 1.80 | 4.71 | 8.19 | 8.98E-03 | -0.36 |
| Thecc1EG037427 | F-box family protein | 0.82 | 4.37 | 8.19 | 8.98E-03 | -0.36 |
| Thecc1EG037802 | Uncharacterized protein | 0.76 | 4.05 | 8.07 | 9.30E-03 | -0.36 |
| Thecc1EG021327 | Calmodulin binding,transcription regulators,putative | -1.23 | 4.89 | -8.31 | 8.63E-03 | -0.36 |
| Thecc1EG005273 | Ribosomal L5P family protein | 0.55 | 5.39 | 8.43 | 8.27E-03 | -0.37 |
| Thecc1EG037279 | Calcineurin B-like protein 10 | -0.63 | 5.93 | -8.47 | 8.23E-03 | -0.38 |
| Thecc1EG011550 | Alpha/beta-Hydrolases superfamily protein | 0.61 | 4.83 | 8.33 | 8.55E-03 | -0.39 |
| Thecc1EG035316 | F-box/RNI-like superfamily protein | -0.64 | 5.22 | -8.36 | 8.49E-03 | -0.40 |
| Thecc1EG037295 | Translation initiation factor SUI1 family protein | 0.63 | 5.73 | 8.42 | 8.28E-03 | -0.40 |
| Thecc1EG043156 | Ubiquitin-like protein 5 | 0.57 | 7.32 | 8.35 | 8.52E-03 | -0.41 |
| Thecc1EG042682 | Disease resistance protein RPP8 | -0.89 | 4.32 | -7.99 | 9.53E-03 | -0.41 |
| Thecc1EG031003 | Disease resistance protein | -0.81 | 5.04 | -8.31 | 8.63E-03 | -0.41 |
| Thecc1EG036833 | Centromere-associated protein E,putative | -0.68 | 4.13 | -7.97 | 9.58E-03 | -0.42 |
| Thecc1EG009828 | C2H2 and C2HC zinc fingers superfamily protein | -1.35 | 4.51 | -8.07 | 9.30E-03 | -0.44 |
| Thecc1EG037259 | Phosphatase 2C family protein | -0.90 | 3.87 | -7.89 | 9.81E-03 | -0.44 |
| Thecc1EG027034 | Toprim domain-containing protein | 0.61 | 4.80 | 8.15 | 9.06E-03 | -0.46 |
| Thecc1EG035293 | Uncharacterized protein | -0.55 | 5.65 | -8.33 | 8.55E-03 | -0.46 |
| Thecc1EG000302 | P-loop containing nucleoside triphosphate hydrolases superfamily protein | -1.19 | 4.12 | -7.91 | 9.76E-03 | -0.46 |
| Thecc1EG029805 | Homeodomain-like transcriptional regulator,putative | -0.52 | 6.07 | -8.34 | 8.55E-03 | -0.47 |
| Thecc1EG045276 | Acidic endochitinase | 1.91 | 5.84 | 8.25 | 8.84E-03 | -0.47 |
| Thecc1EG022629 | GATA zinc finger domain-containing protein C1393.08 | -0.69 | 5.43 | -8.26 | 8.80E-03 | -0.47 |
| Thecc1EG006681 | Amidase family protein | -0.62 | 4.59 | -8.11 | 9.16E-03 | -0.48 |
| Thecc1EG032050 | Leucine-rich repeat transmembrane protein kinase,putative | -0.59 | 7.52 | -8.22 | 8.91E-03 | -0.48 |
| Thecc1EG017829 | Beta-hydroxyisobutyryl-CoA hydrolase 1 | -0.57 | 5.33 | -8.26 | 8.83E-03 | -0.48 |
| Thecc1EG042397 | Plant calmodulin-binding protein-related,putative | -0.66 | 7.05 | -8.27 | 8.77E-03 | -0.49 |
| Thecc1EG022034 | Endomembrane-type CA-ATPase 4 | -0.65 | 5.65 | -8.27 | 8.77E-03 | -0.49 |
| Thecc1EG022931 | UDP-Glycosyltransferase superfamily protein | -0.72 | 6.64 | -8.25 | 8.84E-03 | -0.51 |
| Thecc1EG000503 | Synechocystis YCF37 | 0.69 | 4.51 | 8.03 | 9.40E-03 | -0.51 |
| Thecc1EG000809 | N-acetyl-l-glutamate synthase 1 | 0.59 | 5.15 | 8.17 | 9.02E-03 | -0.51 |
| Thecc1EG031367 | Beta-glucosidase 45,putative | -0.96 | 5.45 | -8.21 | 8.93E-03 | -0.52 |
| Thecc1EG002792 | Uncharacterized protein | 0.61 | 4.90 | 8.11 | 9.16E-03 | -0.52 |
| Thecc1EG029204 | 2-oxoglutarate-dependent dioxygenase family protein,putative | -0.74 | 5.11 | -8.14 | 9.10E-03 | -0.52 |
| Thecc1EG040312 | Peroxidase superfamily protein,putative | 1.02 | 6.75 | 8.21 | 8.93E-03 | -0.53 |
| Thecc1EG046933 | Lycopene cyclase | 0.79 | 3.93 | 7.85 | 9.89E-03 | -0.53 |
| Thecc1EG028818 | Isochorismatase family protein | 0.56 | 6.36 | 8.23 | 8.91E-03 | -0.53 |
| Thecc1EG004556 | Uncharacterized protein | 0.60 | 4.43 | 7.97 | 9.59E-03 | -0.53 |
| Thecc1EG025833 | Amidase family protein | 0.83 | 6.31 | 8.21 | 8.92E-03 | -0.53 |
| Thecc1EG015438 | Plastid transcriptionally active7 | 0.68 | 4.81 | 8.03 | 9.41E-03 | -0.54 |
| Thecc1EG020571 | ARM repeat protein interacting with ABF2 | -0.56 | 6.52 | -8.22 | 8.91E-03 | -0.54 |
| Thecc1EG006833 | Bifunctional phosphatase IMPL2,chloroplastic | -0.62 | 5.60 | -8.16 | 9.06E-03 | -0.55 |
| Thecc1EG006708 | Calcium-binding EF-hand family protein | -0.86 | 7.09 | -8.16 | 9.06E-03 | -0.55 |
| Thecc1EG016556 | Trehalose phosphatase/synthase 5 | 0.69 | 5.97 | 8.19 | 8.98E-03 | -0.55 |
| Thecc1EG004474 | Endoplasmic reticulum auxin binding protein 1 | 1.66 | 4.52 | 7.88 | 9.83E-03 | -0.56 |
| Thecc1EG030081 | C-terminal cysteine residue is changed to a serine 1 | -0.97 | 5.39 | -8.03 | 9.38E-03 | -0.56 |
| Thecc1EG026457 | ARM repeat superfamily protein | 0.75 | 5.25 | 8.02 | 9.42E-03 | -0.58 |
| Thecc1EG039191 | U4/U6.U5 small nuclear ribonucleoprotein 27 kDa protein | 0.66 | 6.36 | 8.15 | 9.06E-03 | -0.58 |
| Thecc1EG021414 | Importin alpha,putative | -0.69 | 5.89 | -8.15 | 9.06E-03 | -0.58 |
| Thecc1EG021914 | Haloacid dehalogenase-like hydrolase (HAD) superfamily protein | 1.21 | 8.20 | 7.97 | 9.59E-03 | -0.58 |
| Thecc1EG026938 | 6-phosphogluconate dehydrogenase family protein | 0.71 | 4.99 | 8.01 | 9.46E-03 | -0.59 |
| Thecc1EG017203 | Ferritin/ribonucleotide reductase-like family protein | 1.22 | 5.30 | 7.93 | 9.70E-03 | -0.60 |
| Thecc1EG024898 | NAD(P)-binding Rossmann-fold superfamily protein | 0.64 | 4.88 | 7.95 | 9.64E-03 | -0.61 |
| Thecc1EG010741 | Serine/threonine protein phosphatase 2A 59 kDa regulatory subunit B' eta | -0.58 | 5.45 | -8.04 | 9.38E-03 | -0.62 |
| Thecc1EG031328 | Uncharacterized protein | 0.51 | 5.59 | 8.08 | 9.25E-03 | -0.62 |
| Thecc1EG037519 | RING-box 1 | 0.65 | 7.55 | 7.99 | 9.53E-03 | -0.63 |
| Thecc1EG036596 | Oxidoreductase family protein | -0.67 | 5.46 | -8.02 | 9.45E-03 | -0.64 |
| Thecc1EG014443 | Porphyromonas-type peptidyl-arginine deiminase family protein | 0.55 | 5.72 | 8.04 | 9.38E-03 | -0.64 |
| Thecc1EG026910 | Ferredoxin--NADP reductase,root-type isozyme,chloroplastic | 0.55 | 5.65 | 8.04 | 9.38E-03 | -0.65 |
| Thecc1EG000756 | Uncharacterized protein family protein SERF | -0.56 | 7.86 | -7.92 | 9.73E-03 | -0.66 |
| Thecc1EG006781 | ATP binding microtubule motor family protein,putative | -0.58 | 5.12 | -7.95 | 9.63E-03 | -0.67 |
| Thecc1EG014776 | Uncharacterized protein | 0.59 | 5.59 | 8.00 | 9.50E-03 | -0.68 |
| Thecc1EG030596 | UPF0172 protein | 0.50 | 6.06 | 8.01 | 9.46E-03 | -0.68 |
| Thecc1EG001074 | F-box with wd-40 2 | 0.53 | 7.76 | 7.90 | 9.76E-03 | -0.68 |
| Thecc1EG034955 | Uncharacterized protein | -1.00 | 6.03 | -7.99 | 9.53E-03 | -0.68 |
| Thecc1EG014729 | Basic helix-loop-helix DNA-binding superfamily protein,putative | -0.81 | 5.18 | -7.91 | 9.76E-03 | -0.69 |
| Thecc1EG000214 | Transducin/WD40 repeat-like superfamily protein | -0.57 | 5.25 | -7.90 | 9.76E-03 | -0.70 |
| Thecc1EG022671 | Uncharacterized protein | 0.67 | 4.82 | 7.83 | 9.95E-03 | -0.70 |
| Thecc1EG005199 | Gamma-tubulin | 0.60 | 5.61 | 7.94 | 9.69E-03 | -0.70 |
| Thecc1EG037614 | UDP-Glycosyltransferase superfamily protein | 0.81 | 7.48 | 7.91 | 9.76E-03 | -0.70 |
| Thecc1EG042492 | Double Clp-N motif-containing P-loop nucleoside triphosphate hydrolases superfamily protein,putative | -0.57 | 5.27 | -7.87 | 9.83E-03 | -0.71 |
| Thecc1EG018525 | Ribosomal protein L34e superfamily protein | 0.55 | 6.75 | 7.92 | 9.73E-03 | -0.72 |
| Thecc1EG008389 | BED zinc finger,hAT family dimerization domain | 0.58 | 5.50 | 7.92 | 9.74E-03 | -0.72 |
| Thecc1EG024602 | Autoinhibited Ca2+-ATPase 11 | -0.57 | 5.11 | -7.87 | 9.83E-03 | -0.72 |
| Thecc1EG024896 | Uncharacterized protein | 0.79 | 5.65 | 7.89 | 9.81E-03 | -0.72 |
| Thecc1EG015109 | IQ-domain 24,putative | -0.69 | 7.18 | -7.89 | 9.80E-03 | -0.73 |
| Thecc1EG019630 | Plant adhesion molecule 1 | -0.54 | 5.76 | -7.93 | 9.73E-03 | -0.73 |
| Thecc1EG017113 | Short-chain dehydrogenase,putative | 0.71 | 5.19 | 7.83 | 9.94E-03 | -0.74 |
| Thecc1EG030423 | Uncharacterized protein | 0.61 | 6.83 | 7.88 | 9.83E-03 | -0.74 |
| Thecc1EG035571 | Duplicated homeodomain-like superfamily protein,putative | -0.58 | 6.07 | -7.91 | 9.76E-03 | -0.74 |
| Thecc1EG008908 | Glucosidase 1 | -0.52 | 5.50 | -7.88 | 9.83E-03 | -0.75 |
| Thecc1EG031194 | S-locus lectin protein kinase family protein | -1.05 | 5.45 | -7.86 | 9.87E-03 | -0.75 |
| Thecc1EG028363 | Cytochrome c1-1,heme protein,mitochondrial | -0.51 | 5.69 | -7.87 | 9.83E-03 | -0.77 |
| Thecc1EG034038 | S-adenosyl-L-methionine-dependent methyltransferases superfamily protein | -0.58 | 6.01 | -7.85 | 9.89E-03 | -0.78 |
| Thecc1EG034048 | 3-ketoacyl-CoA synthase 4 | -0.54 | 6.18 | -7.85 | 9.89E-03 | -0.78 |

**Supplementary table 6.** Analysis of Variance output for a linear model fitting phenotype BLUPs as response variable and the estimated ancestry relative to the cacao populations as predictors

Analysis of Variance Table

Response: yield

Df Sum Sq Mean Sq F value Pr(>F)

Marañon 1 7318 7318 0.1564 0.6931476

Nacional 1 50187 50187 1.0722 0.3022491

Amelonado 1 158611 158611 3.3887 0.0677906 .

Criollo 1 589438 589438 12.5934 0.0005296 ***

Contamana 1 493509 493509 10.5439 0.0014635 **

Nanay 1 528381 528381 11.2890 0.0010085 **

Guiana 1 14374 14374 0.3071 0.5803669

Curaray 1 6096 6096 0.1303 0.7187225

Iquitos 1 11722 11722 0.2504 0.6175618

Residuals 138 6459111 46805

Response: Healthy Pod Count

Df Sum Sq Mean Sq F value Pr(>F)

Marañon 1 27.5 27.54 1.1977 0.27569

Nacional 1 0.2 0.20 0.0085 0.92674

Amelonado 1 11.7 11.70 0.5088 0.47684

Criollo 1 409.3 409.31 17.8029 4.414e-05 ***

Contamana 1 94.4 94.43 4.1072 0.04463 *

Nanay 1 154.5 154.53 6.7213 0.01055 *

Guiana 1 7.9 7.90 0.3437 0.55866

Curaray 1 0.0 0.00 0.0000 0.99748

Iquitos 1 0.0 0.00 0.0000 0.99533

Residuals 138 3172.8 22.99

Response: Proportion frosty pod infected

Df Sum Sq Mean Sq F value Pr(>F)

Marañon 1 0.05998 0.059975 11.2876 0.001009 **

Nacional 1 0.11648 0.116478 21.9218 6.715e-06 ***

Amelonado 1 0.00407 0.004067 0.7654 0.383166

Criollo 1 0.01275 0.012753 2.4002 0.123611

Contamana 1 0.00402 0.004015 0.7556 0.386204

Nanay 1 0.01308 0.013079 2.4615 0.118958

Guiana 1 0.02124 0.021243 3.9980 0.047517 *

Curaray 1 0.02638 0.026381 4.9650 0.027483 *

Iquitos 1 0.01270 0.012697 2.3896 0.124438

Residuals 138 0.73324 0.005313

Response: Proportion black pod infected

Df Sum Sq Mean Sq F value Pr(>F)

Marañon 1 0.0038639 0.0038639 18.5213 3.163e-05 ***

Nacional 1 0.0024634 0.0024634 11.8082 0.0007795 ***

Amelonado 1 0.0003980 0.0003980 1.9077 0.1694500

Criollo 1 0.0000261 0.0000261 0.1250 0.7242310

Contamana 1 0.0001761 0.0001761 0.8441 0.3598203

Nanay 1 0.0003127 0.0003127 1.4991 0.2229023

Guiana 1 0.0000006 0.0000006 0.0029 0.9573551

Curaray 1 0.0000480 0.0000480 0.2299 0.6323286

Iquitos 1 0.0001956 0.0001956 0.9375 0.3346100

Residuals 138 0.0287894 0.0002086

Response: Pod index

Df Sum Sq Mean Sq F value Pr(>F)

Marañon 1 2.6 2.64 0.0255 0.87336

Nacional 1 438.0 438.04 4.2350 0.04148 *

Amelonado 1 313.0 313.00 3.0261 0.08417 .

Criollo 1 65.7 65.70 0.6352 0.42683

Contamana 1 237.5 237.47 2.2958 0.13201

Nanay 1 179.0 179.05 1.7310 0.19046

Guiana 1 35.3 35.31 0.3414 0.55998

Curaray 1 1.6 1.64 0.0159 0.89983

Iquitos 1 0.9 0.94 0.0091 0.92412

Residuals 138 14273.9 103.43

---

Signif. codes: 0 ‘***’ 0.001 ‘**’ 0.01 ‘*’ 0.05 ‘.’ 0.1 ‘ ’ 1

## Supplementary Figures

**Supplementary figure 1**. Minor Allele Frequency (MAF) distribution of all SNP markers. Compared to large diverse population, where genetic variation is generally highest at low MAF, for this panel most SNPs have MAF > 0.05, consistent with recent admixture.

**Supplementary figure 2**. Neighbor-joining tree derived from a genetic distance matrix of all clonal varieties. Full-sib families are shown in blue colors, and inconsistencies between pedigree and tree are highlighted as red branches.

**Supplementary figure 3**. Correlation between Nacional ancestry and proportions of a) frosty and b) black pod infection.

1. **b)**

**Supplementary figure 4**. Correlation between Marañon ancestry and proportions of a) black and b) frosty pod infection.

1. **b)**

**Supplementary figure 5**. Correlation between Nanay ancestry and yield.

**Supplementary figure 6**. Correlation between Contamana ancestry and yield.

**Supplementary figure 7**. Correlation between Criollo ancestry and a) yield and b) healthy pod count.

1. **b)**

**Supplementary figure 8**

Distributions of the mean proportion of black and frosty infected pods per clone across all evaluated years. The distribution of the proportion of black pod infection has a very low mean of 0.018, compared to a mean value per clone of 0.13 for frosty pod.

**Supplementary figure 9**: Distribution of the sum of mapped read counts on the 45,600 gene models per sample. The read counts vary from 2,630,962 to 37,694,247. Between 20,217 and 25,927 genes had no reads mapped to them in a sample. The three smaller samples have very different sequencing depths; therefore, careful scaling is needed for the read counts of the samples to be comparable.

**Supplementary figure 10**: Differential expression score distribution from RoDEO for UF273 between technical replicates (blue), biological replicates (green), and 8 h vs. 48 h time-points for two different trees (orange, yellow). The highest DE score between technical replicates is 7, and that between biological replicates is 10. From these data, significant differential expression was defined as a DE score > 10.
